# Chronological reassessment of the Middle to Upper Paleolithic transition and Early Upper Paleolithic cultures in Cantabrian Spain

**DOI:** 10.1371/journal.pone.0194708

**Published:** 2018-04-18

**Authors:** Ana B. Marín-Arroyo, Joseba Rios-Garaizar, Lawrence G. Straus, Jennifer R. Jones, Marco de la Rasilla, Manuel R. González Morales, Michael Richards, Jesús Altuna, Koro Mariezkurrena, David Ocio

**Affiliations:** 1 Instituto Internacional de Investigaciones Prehistóricas de Cantabria, (Universidad de Cantabria, Santander, Gobierno de Cantabria), Santander, Spain; 2 Leverhulme Centre for Evolutionary Studies, Department of Archaeology, University of Cambridge, Cambridge, United Kingdom; 3 Centro Nacional de Investigación de la Evolución Humana, Burgos, Spain; 4 Department of Anthropology, MSC01 1040, University of New Mexico, Albuquerque, New Mexico, United States of America; 5 Departamento de Historia, Universidad de Oviedo, Oviedo, Spain; 6 Department of Archaeology, Simon Fraser University, Burnaby, British Columbia, Canada; 7 Centro de Conservación e Investigación de los Materiales Arqueológicos y Paleontológicos de Gipuzkoa, Donostia/San Sebastián, Spain; 8 Department of Civil and Environmental Engineering, Imperial College London, London, United Kingdom; New York State Museum, UNITED STATES

## Abstract

Methodological advances in dating the Middle to Upper Paleolithic transition provide a better understanding of the replacement of local Neanderthal populations by Anatomically Modern Humans. Today we know that this replacement was not a single, pan-European event, but rather it took place at different times in different regions. Thus, local conditions could have played a role. Iberia represents a significant macro-region to study this process. Northern Atlantic Spain contains evidence of both Mousterian and Early Upper Paleolithic occupations, although most of them are not properly dated, thus hindering the chances of an adequate interpretation. Here we present 46 new radiocarbon dates conducted using ultrafiltration pre-treatment method of anthropogenically manipulated bones from 13 sites in the Cantabrian region containing Mousterian, Aurignacian and Gravettian levels, of which 30 are considered relevant. These dates, alongside previously reported ones, were integrated into a Bayesian age model to reconstruct an absolute timescale for the transitional period. According to it, the Mousterian disappeared in the region by 47.9–45.1ka cal BP, while the Châtelperronian lasted between 42.6k and 41.5ka cal BP. The Mousterian and Châtelperronian did not overlap, indicating that the latter might be either intrusive or an offshoot of the Mousterian. The new chronology also suggests that the Aurignacian appears between 43.3–40.5ka cal BP overlapping with the Châtelperronian, and ended around 34.6–33.1ka cal BP, after the Gravettian had already been established in the region. This evidence indicates that Neanderthals and AMH co-existed <1,000 years, with the caveat that no diagnostic human remains have been found with the latest Mousterian, Châtelperronian or earliest Aurignacian in Cantabrian Spain.

## Introduction

The Middle to Upper Paleolithic transition is generally associated with the replacement of local Neanderthal populations by Anatomically Modern Humans (AMH) in western Eurasia [1]. One of the most debated issues is the precise chronology of the replacement of the population and its potential relationship with major biological, environmental and cultural events. Due to recent revisions of archaeological, chronological and geographical data, the spatio-temporal delimitation of the Middle to Upper Paleolithic transition in central and western Europe has improved [1–5]. Methodological developments in radiocarbon dating that remove young carbon contamination from old organic samples [6–7] have been crucial for establishing accurate chronologies, especially at the radiocarbon limit around ~50,000 years ago, when the Middle to Upper Paleolithic transition took place. Thus, the re-dating of several key sites in Europe has produced older ages than previously thought [1]. The timing of Neanderthal disappearance in northwestern Iberia (~48-46ka uncal BP) and Italy (~44-42ka cal BP) and the age of so-called “transitional industries” (Châtelperronian and Uluzzian, both 44 to 40ka cal BP) have been recently delimited [1,3]. While the attribution to Neanderthals of the Châtelperronian in northern Iberia and southwestern France is seldom contested (but see [8]), the question of the makers of the Uluzzian is still debated. Human deciduous teeth found in Southern Italy (Grotta del Cavallo) suggest that AMH were the makers of the Uluzzian [9], whereas other researchers continue to argue for Neanderthal authorship [10]. Those “Transitional techno-complexes” (especially the Châtelperronian), have been interpreted as an evidence of a short period of coexistence between local and immigrant human populations [1, 11], with the early phases of the Aurignacian complex (Proto-Aurignacian and early Aurignacian) being attributed to the first modern humans in Europe, as early as 42 ka cal BP, during GI10. In Iberia, the end of the Mousterian has been shown to have been earlier in the North than once thought, while the Early Upper Paleolithic in the South (Gravettian) is older than was previously believed [12]. Thus, the hypothesis of a very early appearance of Aurignacian in northern Iberia is no longer supported [2,4].

Here, we focus on the Cantabrian region in northern Iberia, where the archaeology, stratigraphy and chronology of several key sites—El Castillo, Labeko Koba, La Viña, Esquilleu and Morín—have been recently reviewed [2,5,13], providing an initial framework for the Middle to Upper Paleolithic transition. However, further research at more representative sites in this region with Mousterian, Châtelperronian, Aurignacian and Gravettian materials, that either lacked radiocarbon dates or had dates obtained in the late 20th century before the development of rigorous pre-treatment methods, is needed to securely establish the chronology by including more archaeological sites. As part of a wider project to reconstruct the climate and local and regional environmental conditions that late Neanderthals and early AMH faced in the region during Marine Isotopic Stage 3 (MIS3), using stable isotopic analyses (δ^13^C, δ^15^N, δ^18^O and δ^34^S), first we performed a complete review of the chronological data from 13 regional sites that included 28 Middle and Early Upper Paleolithic archaeological levels. Out of 51 samples that were processed for radiocarbon dating, 5 failed and the remaining 46 received an AMS measurement using new ultrafiltration method that is more rigoruous in removing possible bone collagen contamination, from contemporaneous sites which either lacked dates or had previously produced problematic dates.

The new ^14^C radiocarbon dates obtained in this project, in combination with other available ultrafiltered dates from the region, have allowed the development of an independent, radiometric chronology of previously undated sites in the Cantabrian region, which had until now been attributed only through stratigraphic position and/or material culture characteristics to the Mousterian, Châtelperronian, Aurignacian or Gravettian techno-complexes. Re-dating of sequences that had previously been dated using conventional or early AMS ^14^C methods was undertaken, producing significant changes in previous interpretations of relevant sites, something that has also been verified by reviewing the lithic technocomplexes. The results provide greater temporal precision for the Middle to Upper Paleolithic transition in the Cantabrian Region by building a Bayesian model using samples treated with a robust pre-treatment methodology. The model includes new Mousterian modelled dates within the range of the calibration curve and defines the boundaries for the start and end of each of the Early Upper Paleolithic techno-complexes.

This research presents new possibilities for addressing the timing of the critical processes of cultural change, local extinctions and population replacement that happened during the Middle-Upper Paleolithic transition in northern Spain and more generally in Europe. The dating results reported here will also serve to discuss the stratigraphic integrity and cultural attribution of the sites analysed.

## Material

**Fig 1:**
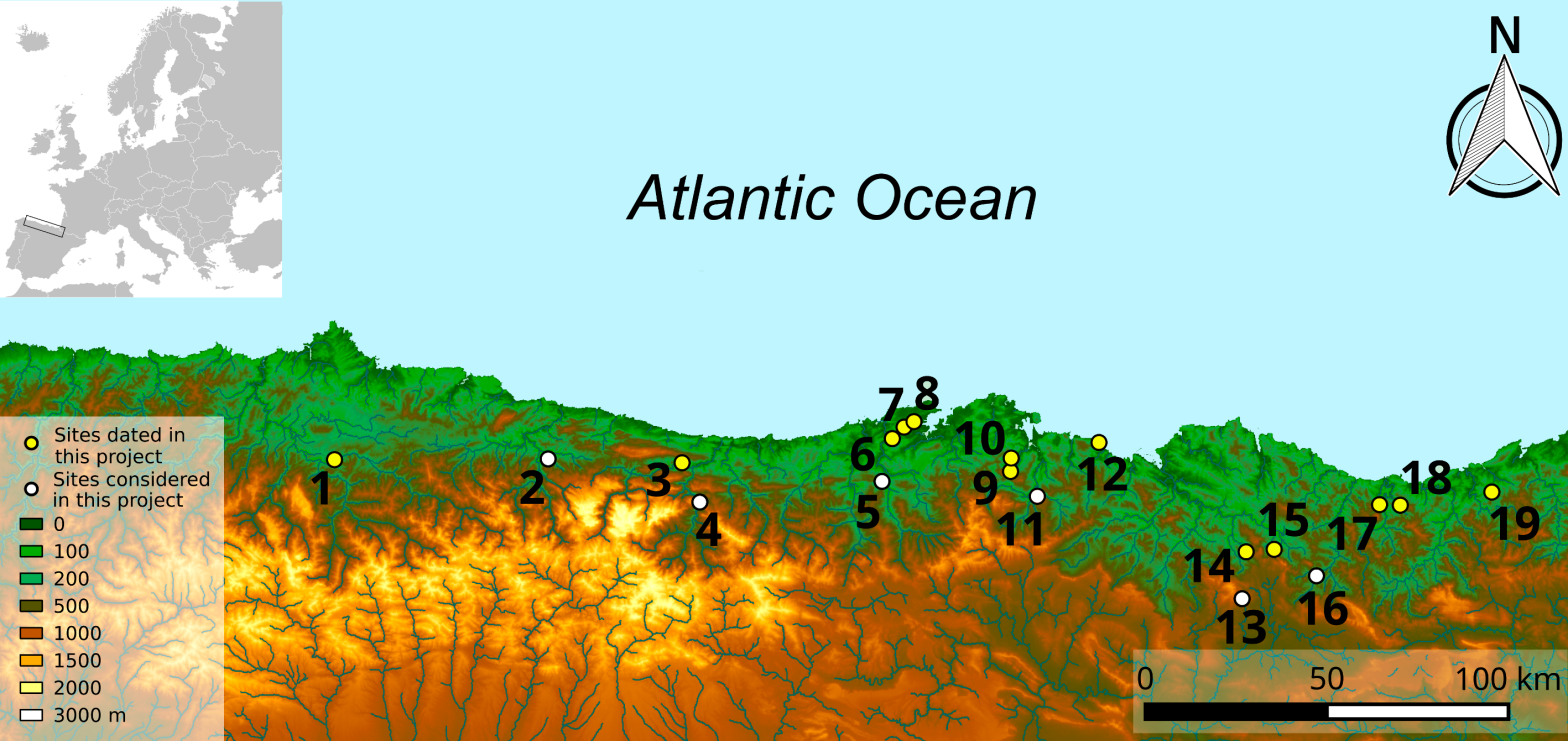
Location of sites in the Cantabrian region, northern Spain. 1: La Viña, 2: La Güelga, 3: Llonín, 4: Esquilleu, 5: El Castillo, 6: Covalejos, 7: Ruso I, 8: Morín, 9: Cobrante, 10: El Otero, 11: Mirón, 12: El Cuco, 13: Arrillor, 14: Axlor, 15: Bolinkoba, 16: Labeko Koba, 17: Ekain, 18: Amalda, 19: Aitzbitarte Cave III.

Thirteen sites were dated as part of this project, including a total of three Middle Paleolithic and twenty-five early Upper Paleolithic archaeological levels. These sites are located in the modern-day regions of Asturias in the west, Cantabria in the center and the Basque Country in the east of the Cantabrian Region (Fig 1). These archaeological levels were selected because of their attribution to the Mousterian (n = 3), Châtelperronian (n = 1), Aurignacian (n = 11) and Gravettian (N = 13) techno-complexes, previously determined based on their stratigraphic position, material culture, available dating or a combination of these factors. The Mousterian levels dated were Amalda VII [14], Axlor IV [15] and Llonín VI [16–20], while the only Châtelperronian level was Ekain Xa [21]. The Aurignacian levels were Aitzbitarte (Cave III-entrance area) Vb center [22], Ekain IXb, Cobrante V and VI [23], El Ruso Cave I IVb [24], El Otero IV, V and VI [25], Morin 7c [26], Covalejos B (2) and C (3) [27]. Finally, the Gravettian levels were Aitzbitarte (Cave III-entrance area) IV, Va and Vb upper, Amalda V and VI, Bolinkoba VI/F [28–29], El Cuco III and Vb [30–31], La Viña VII, VIII, IX and X [32–36] and Llonín V. Other regional sites with contemporaneous cultural attributions, but unclear stratigraphy, insufficient archaeological information, or those that are currently still under study were not included here. We also took into consideration the radiocarbon dates recently published for the regional sites of La Viña, Esquilleu, La Güelga, El Castillo, Morín, El Mirón, Arrillor and Labeko Koba [2–5,37–38] that have been achieved using the same ultrafiltration method as this study, making them comparable and appropriate to include in the Bayesian models. We have initially accepted the culture-stratigraphic designations for individual levels proposed by the excavators/analysts of the various sites. This brings with it the consequence of incorporating differing classificatory criteria (including differing approaches to “lumping” versus “splitting” in such designations) among the many different researchers who studied the various artifact assemblages, some of which contain more definitive temporally/culturally bounded and thus diagnostic artifacts than others. However, the Bayesian approach applied here has the capacity to challenge these prior attributions, prompting a reinterpretation of the lithic assemblages and the stratigraphy. Where mismatches were identified, sites were revisited, and if necessary, attributions changed according to our reviews. In case of doubt, dates were discarded to avoid any bias in the conclusions.

51 samples were processed for radiocarbon dating on bones with evidence of human manipulation such as anthropogenic fractures and/or cut marks. Sample information for each specimen, including site, archaeological level, context, sample number, animal species and anatomical element is included in S1 Table. The location of the archaeological material in regional museums and institutions is indicated in the description of each site in S1 File. This research implies a remarkable increase in the number of reliable radiometric dates in the region and period of study, which currently comprises 61 determinations [4,5,12–13] [S3 Table]. The selection of the fauna samples in museum collections was based on the following criteria: 1) stratigraphic position of the remains at the site; 2) selection of bones preferably directly labelled to assure their provenance within the site; 3) selection of animal bones taxonomically and anatomically identifiable and 4) selection of anatomical elements with clear anthropogenic marks.

## Methods

### Radiocarbon dating

Prior to radiocarbon dating, a subsample of all specimens was analysed to obtain δ^13^C and δ^15^N values, which also established the state of collagen preservation. Of the bones previously analysed for stable isotopic analyses, specimens yielding >1% collagen were selected for dating to maximise the likelihood of achieving a successful radiocarbon date. All samples met the quality assurance criteria as shown in S1 Table. Collagen extraction and analysis for radiocarbon dating were undertaken at the Oxford Radiocarbon Accelerator Unit (ORAU). Collagen was obtained following the method detailed by Brock et al [39], which involves the demineralisation of the mineral component (and any exogenous carbonates) of drilled bone power using 0.5M HCL at 5°C overnight, before removal of organics and humic acid using 0.1M NaOH solution for 30 minutes at room temperate (RT), and a final wash in 0.5M HCL for 1 hour at RT. The collagen was gelatinised in a 0.001M HCL solution for 20 hours at 70°C. EZEE^™^ biological filters (45–90 μm) were used to remove smaller soluble components, before ultrafiltration using cleaned 30 kDa MWCO Vivaspin^™^ 15 ultrafilters. Combustion of the collagen using an elemental analyser (ANCA-GSL), linked to an isotope ratio-mass spectrometer (Sercon 20–20) produced Carbon and Nitrogen stable isotope data, and samples were dated using an Accelerator Mass Spectrometer following conversion of excess CO_2_ into graphite using an iron catalyst [40].

### Bayesian modelling

Individual Bayesian age models could be built for La Viña (S1 Fig), Covalejos (S2 Fig), El Cuco (S3 Fig), Ekain (S4 Fig) Amalda sites (S5 Fig) and Aitzibitarte (Cave III) (S6 Fig) using OxCal4.2 software [41] and the INTCAL13 calibration curve [42]. No chronological models were built for the other sites, because of the limited number of dates. The Bayesian model enables to modify the calibrated Probability Distribution Function (PDF) of individual dates based on the existing relative stratigraphic and other relative age information. Both new ultrafiltration dates and previous ones recently published for the period of study in northern Spain were considered for completeness (see S2 Table). The presence of problematic determinations that do not agree with the prior framework was considered by adopting a t-type outlier model with an initial 5% probability for each determination to be an outlier. Likewise, a s-type outlier model was used to test the coherence of two radiocarbon dates obtained from the same bone remain [43]. A resolution of 20 years was assumed, being a reasonable balance between required accuracy and computational costs, and a sensitivity test on the outlier model and prior boundary test was conducted to ensure the robustness of the results. A discussion of each archaeological site and cultural attribution according to the dates are discussed individually in S1 File. CQL codes are also reported there (S1 and S2 Codes). An Order function in the OxCal was used to calculate the probability that one PDF predated another, providing information to assess synchronicity and temporal overlap of individual archaeological levels and cultural phases in each of the six individual sites modelled. In all cases, convergence was greater than 95% and the model agreement index close to 100% except in the case of La Viña, where it is still clearly above the 60% validity threshold.

Once models for individual sites were conducted, modelled dates from each industry (posterior PDFs) were grouped within a single cultural phase (inserted as priors) without assuming any order. This was achieved by placing them within a single Phase with start and end boundaries. Individually calibrated dates coming from sites were no individual site model could be conducted (such as Cobrante Level VI, Llonín V, Bolinkoba VI/F and Morin 4), as well as other existing reliable modelled dates in the region (from Labeko Koba [4], El Castillo [5] and La Viña [13]), were included. These new unordered Bayesian age models enabled determining a regional PDF of the temporal boundaries between Mousterian, Châtelperronian, Aurignacian and Gravettian, as well as an easier comparison between sites. The advantage of this analysis in comparison with individual models is that it enables further constraining the uncertainty of the boundaries by assuming that the onset and disappearance of cultures were regional processes rather than localised phenomena. This is reasonable given the known level of regional interconnection during Late Pleistocene in northern Iberia. Likewise, it reduces the potential bias at one particular site, as pooling together several sites in one region serves to increase the reliability of the results, which can be interpreted regionally with more confidence (see S11 Figure in [3] and see Figure 8 in [5]). The difference between the PDFs of the start and end boundaries was also calculated to estimate the likely duration of the phase. The results were compared with the Greenland Oxygen Isotopic record (NGRIP) [44,45] as a global climatic record to define the different cultural periods in the Cantabrian region.

All Bayesian models were run 4–5 times and results compared to check consistency. They disclosed acceptable levels of reproducibility when compared, although key boundary parameters were usually within 50–100 years of one another with repetition of the model. This is the usual accuracy expected when using this approach, and consequently, all dates reported here have been rounded to the nearest century. Finally, the youngest and oldest dates for each cultural period were removed and Bayesian models were rerun to test the dependence of the conclusions on them.

## Results and discussion

Out of the 51 dates processed for radiocarbon dating, 46 produced successful AMS measurements as they had enough collagen to provide viable results. Four samples failed due to low collagen yields and one due to a high C:N value. Collagen was generally well preserved and only one sample from El Cuco yielded less than 1% collagen, but it had a C:N ratio of 3.39 was within the acceptable range for *in vivo* collagen [46]. Of the 46 dates, 3 dates were beyond the radiocarbon limit and 13 showed mismatches with their defined cultural attribution providing a chronology younger than initially attributed either by cultural artefacts found within that level or previous dating (S1 Table). Consequently, in this study, a substantial dataset of 30 valid new dates was obtained and only with those dates Bayesian models were generated.

The 13 dates that show discrepancies between the cultural attributions determined by the new dates obtained and those based on traditional analyses of the archaeological materials correspond to 7 of the 13 studied sites (11 of the 28 levels). This might be caused by one or several reasons such as excavation methods (e.g., failure to see or identify stratigraphic differences, attributions based on very small, not very diagnostic artifact collections from limited excavations or test pits) or curatorial issues with the faunal material, as several of the regional museums have been moved repeatedly to different locations over several decades. For each site, possible reasons for those inconsistencies are discussed in S1 File.

Individual Bayesian models were created for sites where samples belonged to more than one layer, such as La Viña (S1 Fig), Covalejos (S2 Fig), El Cuco (S3 Fig), Amalda (S5 Fig) and Aitzbitarte III (S6 Fig). For Ekain two dates for Level IXb were also modelled (S4 Fig). CQL codes are presented in S1 and S2 Codes. This allowed, considering the stratigraphy of the sites as prior information for the dates, leading to more robust results. The robustness of the results has also been verified by means of a sensitivity test (see S4 Table), which resulted in marginal changes to the relevant boundaries between cultures.

In several archaeological levels, typological chronologies have been challenged by the new dating evidence. Thus, in Asturias, the site of Llonín Level V, attributed to the Gravettian [16–20], yielded two very different results: one coherent with an Early Gravettian attribution (~28k uncal BP) and another date of 20k uncal BP, which could correspond to an early Solutrean or a very late phase of the Gravettian, younger than Morín Level 4 [47] and similar to the AMS dates of Level III from Aitzbitarte III [48]. These results suggest either a much longer formation for Level V (more than 8,000 years for only 9 cm of stratigraphy), although we cannot dismiss the possibility of some disturbance or admixture with overlying Solutrean Level IV which remains undated. In the Autonomous Community of Cantabria, the new chronology of El Otero, which was previously undated, indicates a Magdalenian chronology (~15–10 ka uncal BP, and hereafter) instead of an Aurignacian one as traditionally proposed [25]. Despite the presence of apparently characteristic artifacts [25,49–50], this result urges caution in the dating of levels solely using artifacts that are not temporally diagnostic. In Level V of Cobrante, identified as Aurignacian, despite its lack of characteristic material culture [51] the obtained dates correspond to the Solutrean instead (~18k uncal BP). The apparent absence of characteristic Aurignacian lithics, and the presence of at least one piece with partial invasive (Solutrean) retouch [51], support this attribution. Cobrante Level VI was interpreted as Archaic Aurignacian because of the presence of characteristic lithic tools, such as Dufour bladelets, large “Aurignacian” blades and carinated end-scrapers, which appeared alongside Mousterian-looking tools [51]. New two dates show an incoherence between them: one with a date beyond the radiocarbon limit and another with a contemporaneous regional Early Aurignacian date. Curational or stratigraphical issues could be the reason for this incoherence as explained in S1 File. In El Cuco, the Levels III and Vb previously defined [30] both as Gravettian provided Aurignacian (~35k uncal BP) and Mousterian (~49k uncal BP) dates, respectively. The lower levels of this sequence (VII-XIII) have been recently reassessed through new 14C AMS dating on shells and a technological lithic study. These new analyses confirm a Mousterian attribution to levels previously defined as Aurignacian and an Aurignacian chronology to levels defined as Gravettian [31]. In El Ruso, Level IVb originally defined as Evolved Aurignacian [52], provided a Gravettian date (~28k uncal BP). Although the small lithic assemblage is not very diagnostic, the presence of a flat-nosed end-scraper and a double carinated end-scraper was mentioned.

In Bizkaia, the Gravettian Level VI of Bolinkoba provided a contemporaneous date for this regional period, while a second one is much younger (~10k uncal BP), attributable to the regional Azilian, suggesting significant mixing or curational problem as no diagnostic Azilian lithic material has been recovered or identified in Level VI assemblage [53]. In Amalda (Gipuzkoa), three dates provided younger dates than expected [54]. One sample from Mousterian Level VII provided a date within a Gravettian time range (~28k uncal BP), possibly reflecting a limited admixture between Levels VI and VII [55]. Another sample from the Gravettian Level VI gave a medieval date. Its location close to the cave entrance, where the upper levels were exposed, points towards the possibility that materials from historic times were inserted into Last Glacial sediments. A further sample from the Gravettian Level V provided a Magdalenian age (~14k uncal BP), maybe deriving from unknown problems during excavation or curation. In Aitzbitarte cave III Level Vb (center) yielded a single date that is older than the normative attribution (likely Early Aurignacian rather than Evolved Aurignacian) [56], evidencing a possible presence of an Early Aurignacian at the base of the level. In Ekain (Gipuzkoa), Level Xa, undated, was interpreted as a Châtelperronian hunting camp, with a small lithic collection, including a single typical Châtelperronian point, and three other backed blades and bladelets [57]. The new date for Level Xa is 34 ka uncal BP, which is far too young for the regional Châtelperronian, dated at 37-38k uncal BP in nearby Labeko Koba [4]. The location of the bone sampled at the site and the significant presence of cave bears do not rule out the possibility of admixture caused either by those carnivores or by other post-depositional processes not identified during the excavation of Ekain.

These discordances are particularly relevant for sites profusely used in the past to investigate the cultural adaptations during the Cantabrian Upper Paleolithic and reflect the complexities of attributing archaeological levels based solely on lithic typology or technology. It also reveals the problems caused by the attributions based on single dates without taking into account the characteristics of the archaeological assemblage.

**Fig 2:**
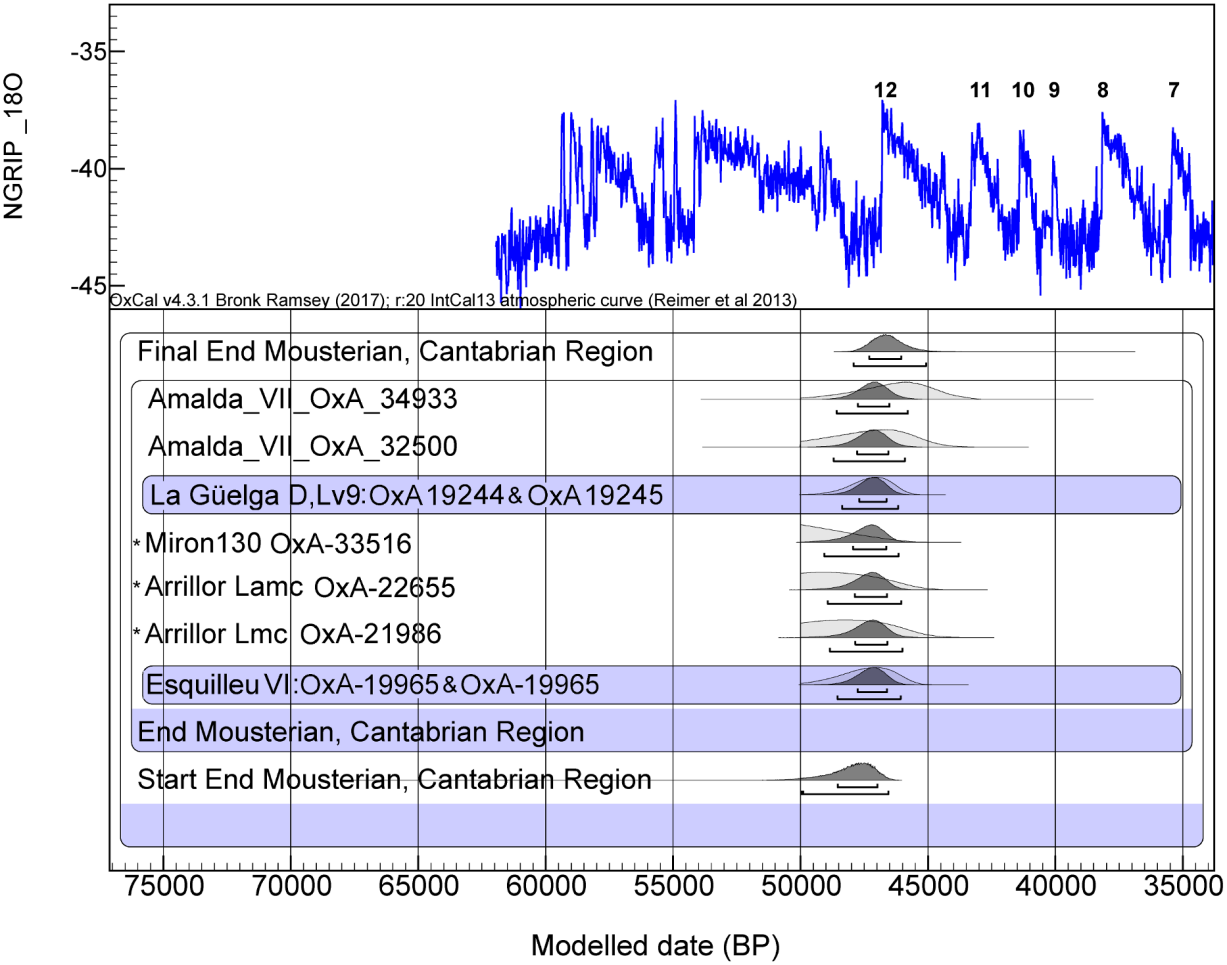
Radiocarbon dates for the uppermost dates of the Mousterian assemblages from the Cantabrian region (Asturias, Cantabria and the Basque Country) calibrated against IntCal13 [42] in OxCal v.4.2 [41] assuming each sample has a 5% prior probability of being and outlier within the general t-type outlier model [43]. All dates have been obtained using the ultrafiltration protocol [40]. References are given in S1 Table. A_model_ = 99.8. *denotes previously unmodelled dates introduced as R-dates while the others are modelled dates at individual sites introduced as Prior PDFs.

**Fig 3:**
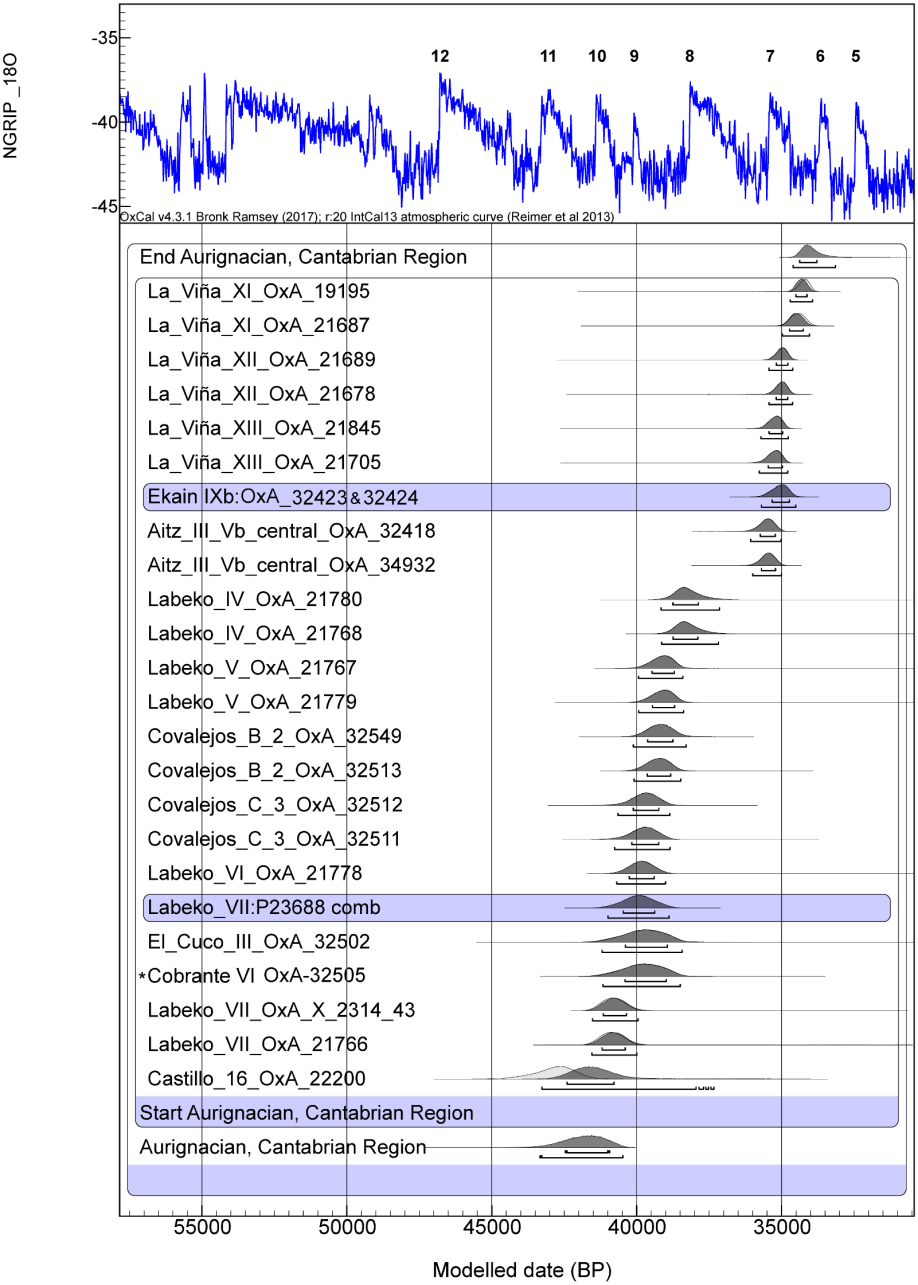
Radiocarbon dates for the Aurignacian assemblages from the Cantabrian region (Asturias, Cantabria and the Basque Country) calibrated against IntCal13 [42] in OxCal v.4.2 [41] assuming each sample has a 5% prior probability of being and outlier within the general t-type outlier model [43]. All dates have been obtained using the ultrafiltration protocol [40]. References are given in S1 Table. A_model_ = 84.2. *denotes previously unmodelled dates introduced as R-dates while the others are modelled dates at individual sites introduced as Prior PDFs.

**Fig 4:**
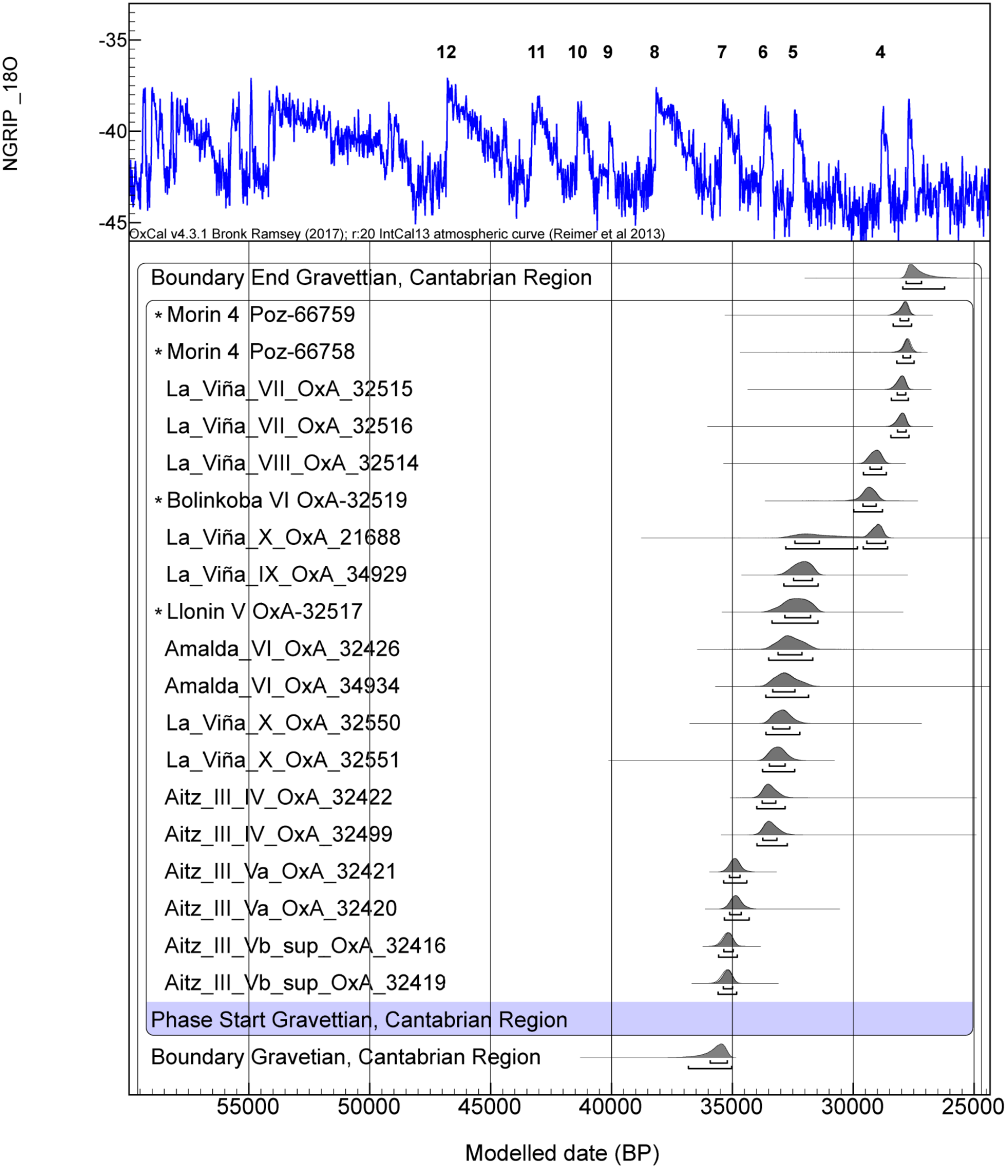
Radiocarbon dates for the Gravettian assemblages from the Cantabrian region (Asturias, Cantabria and the Basque Country) calibrated against IntCal13 [42] in OxCal v.4.2 [41] assuming each sample has a 5% prior probability of being and outlier within the general t-type outlier model [43]. All dates have been obtained using the ultrafiltration protocol [40]. References are given in S1 Table. A_model_ = 104.4. *denotes previously unmodelled dates introduced as R-dates while the others are modelled dates at individual sites introduced as Prior PDFs.

The regional chronological framework for each cultural period as obtained from the Bayesian age model is presented in Figs 2–4. No regional model for the Châtelperronian is included as only ultrafiltered dates are available for Level IX lower of Labeko Koba and this site has been modelled elsewhere (see Figure S20 in [3]). Regarding the chronology of the Late Mousterian in the Cantabrian region, the dates from Axlor go beyond the radiocarbon limit and samples from Llonín Level VI failed. However, two new dates, both from Level VII in Amalda, add important new evidence to the existing record. The updated Bayesian model for the Upper to Middle Paleolithic transition shows that the end boundary PDF for the Mousterian in the region is 47.9–45.1ka cal BP (all probability ranges are expressed at 95,4% hereafter), confirming that it occurred earlier in the Atlantic zone than in the northeastern Iberia and in western and northern Europe [3]. The apparent end of the Mousterian came immediately before and during GI12 (Fig 2). No Châtelperronian dates were obtained in this project, despite Level Xa of Ekain having originally been defined as Châtelperronian based on lithic typology. However, the date obtained from Ekain was too young for the regional range of the Châtelperronian, which is found in the Cantabrian Region in Morín and Labeko Koba, but is only reliably dated in the latter at 42.6–41.4ka cal BP (see SI pp.47 in [3]).

The earlier disappearance of those Mousterian groups in the Cantabrian region (northern Atlantic Iberia), while they still survived in Catalonia (north-eastern Mediterranean Iberia) for a few more millennia until just before 42 cal BP [2,58], as dated in Romani and L`Arbreda, might have been caused by as yet unknown factors and requires further research.

The age model data reveals that there was an interval of 2,700 to 5,800k (95.4% probability), with a median of 4,400 years between the end of the Mousterian and the start of the Châtelperronian in the Cantabrian Region (Table 1). The Châtelperronian appears to start several millennia after the Mousterian completely disappeared in the region, while in France this “transitional” technocomplex appears before the end of the Mousterian [2]. If Neanderthals were responsible for both Mousterian and Châtelperronian technocomplexes in the Cantabrian region, this would suggest a Châtelperronian intrusion from southwestern France to the eastern (in Labeko Koba) and central (in Morín) sectors of Cantabrian Spain, after Mousterian populations had already disappeared. This could be due to pressure from expanding populations of modern humans, but any hypothesis of this sort requires further exploration.

**Table 1:** Results of the 68% and 95% PDF range of the boundaries, duration of technocomplexes and temporal lapse between archaeological phases.

	Modelled ranged (cal BP)				
	(68.2% prob.)		(95.4% prob.)		
	From	To	From	To	
**Boundaries**					
End Mousterian	47,298	46,048	47,914	45,078	
Start Châtelperronian	42,508	41,930	42,868	41,686	
End Châtelperronian	42,142	41,632	42,406	41,370	
Start Aurignacian	42,406	41,000	43,270	40,478	
End Aurignacian	34,376	33,784	34,604	33,140	
Start Gravettian	35,914	35,206	36,818	35,030	
**Duration of industry**					
Châtelperronian	0	720	0	1,160	
Aurignacian	7,152	8,788	6,500	9,840	
**Time between industries**					**Median**
End Mousterian/Start Châtelperronian:	3,704	5,100	2,662	5,752	4,354
Start Aurignacian / Start Châtelperronian:	-482	1,122	-1,404	1,768	274
End Châtelperronian/Start Aurignacian:	-798	740	-1,750	1,400	-90
End Aurignacian/Start Gravettian:	-2,172	-1,080	-3,258	-756	-1,702
End Mousterian/End Châtelperronian:	4,050	5,436	3,028	6,116	4,714
End Mousterian/ Start Aurignacian:	3,674	5,646	2,340	6,516	4,588

The Bayesian modelled results suggest that the end boundary for the Châtelperronian was 42.4–41.4ka cal BP (S1 Table). According to the dating results, the Châtelperronian phase was relatively short (with the caveat that only one level is dated by ultrafiltration within the whole region) and partially overlaps with the start of the Aurignacian. Châtelperronian dates range between 42.8 and 41.4ka cal BP which coincides in time with its final boundary due to the short duration. The interval between the end of the Châtelperronian and the start of the Aurignacian is 1,400 years (Table 1). If Neanderthals were responsible for the Châtelperronian and AMH for the Aurignacian, the implication is that there was a short duration of overlap between the Châtelperronian and Aurignacian, indicating a brief period of coexistence between both human species with a quick replacement of the Neanderthals in this region. However, there is a lack of diagnostic human remains in the region in association with any of these techno-complexes and “authorship” of these will only be fully demonstrated with the discovery of skeletal evidence.

The start boundary for the Aurignacian techno-complex in the region falls between 43.3–40.5ka cal BP and the end at 34.6–33.1ka cal BP (95.4% probability) (Fig 3 and S2 Table). This chronology is consistent with Zilhao’s suggestion [59] that the occurrence of the Aurignacian in northern Iberia was before GI10, around c.42ka cal BP. The Proto-Aurignacian at El Castillo appears during GS11, which is earlier than in Labeko Koba, although the OxA-22200 date in Level 16 of 38,600 ± 1,000 is found to have a 15% likelihood of being an outlier. Cobrante, which contains diagnostic lithic elements corresponding to a Proto-Aurignacian technocomplex, is situated between the Proto-Aurignacian dates of Labeko Koba Level VII. The high-precision chronometric dates show a distinction between Proto and Early Aurignacian assemblages and Evolved Aurignacian ones in this region. The exception to this is El Castillo, where the Proto and Early Aurignacian occurred during GI10 and GI9, and before H4. After the Proto-Aurignacian, the Early Aurignacian appears to spread rapidly after GI9 through GI8. In Covalejos, a quick replacement between Proto (Level C/3) and Early Aurignacian (Level B/2) is also apparent. The most recent phase of the Aurignacian techno-complex, the Evolved Aurignacian, occurred in the region during or immediately after GI8, starting first in the east at the sites of Aitzbitarte III and Ekain and spreading west as far as La Viña (Fig 3). Despite the proposal that the combination of Heinrich Event 4 with the Campanian Ignimbrite eruption (CI) and the Bond cooling trend might have destabilised the ecological niches and the distribution of biotic resources during MIS 3, acting as a possible triggering factor for the demise of the Neanderthals [60–62], this might not be true for the Cantabrian region, as the Neanderthals had apparently already disappeared some time earlier. However, those climatic oscillations may have affected the movement of modern humans from eastern and central Europe through southern France and into northern Iberia, as they expanded into new, resource-rich territories such as the Cantabrian and Catalonian regions, where Neanderthal populations may already have been either sparse or non-existent.

By comparing the start and end boundary PDFs for both Gravettian and Aurignacian, the Gravettian appears to have started before the end of the Aurignacian around 36.8-35ka cal BP, during GI7, immediately after the first Late Aurignacian assemblages appeared in the region (Table 1, S1 and S2 Tables). The interval between the end of Aurignacian and the start of the Gravettian ranges from -3300 to -800 years, might indicate a long overlap between the two techno-complexes. By looking at the spatio-temporal distribution of the Gravettian in the region, the earliest occurrences appeared in the Basque Country at Aitzbitarte III, which might indicate, first, an early arrival of Gravettian artifacts from western Pyrenean France into Vasco-Cantabria or a local origin within the eastermost part of the region (including Bizkaia, Gipuzkoa and French Basque country) and, second, a dispersal of the technocomplex from east to west. In fact, the earliest Gravettian dates are in Aitzbitarte III at the eastern end of the Basque province of Gipuzkoa (35.6–34.8ka cal BP), whilst a much later start is seen in the westernmost site, La Viña, in Asturias (33.7–32.4ka cal BP) (Fig 4). These Gravettian dates would indicate the oldest directly dated evidence of this technocomplex in Western Europe.

The conclusions given above are not significantly altered when the oldest and youngest dates are removed from each cultural phase (see S5 Table), apart from the estimated overlap between the Châtelperronian and the Aurignacian. The start of the Aurignacian is dependent on the validity of the date obtained from Level 16 of El Castillo. Without that date, the Aurignacian would be pushed forward around 1,200 years, reducing drastically the probabilities of a coexistence of both cultures in the region. The cultural attribution of that level to Proto-Aurignacian is clear [63] and the date was obtained with ultrafiltration methodology with no problems arising from its analysis [5]. However, more dates would be desirable to confirm the potential overlap between the Châtelperronian and the Aurignacian in the region.

## Conclusion

**Fig 5:**
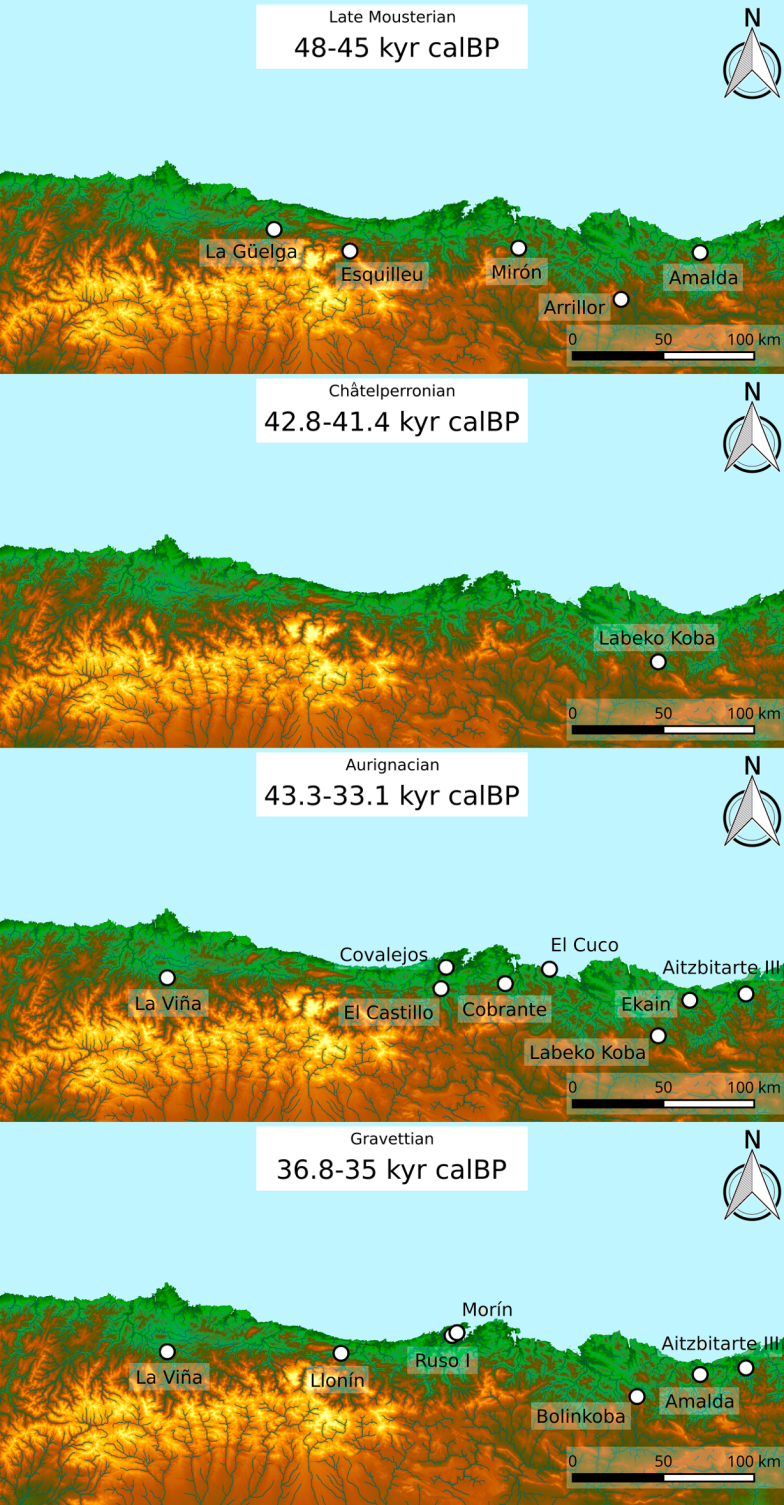
A spatio-temporal comparison of the late Mousterian, Châtelperronian, Aurignacian and Gravettian assemblages from the Cantabrian region (Asturias, Cantabria and the Basque Country).

The new radiocarbon dates and the subsequently derived Bayesian models that include both new and previously run high-quality dates, provide high-precision chronological resolution for reconstructing the spatio-temporal evolution of the Middle to Upper Palaeolithic transition in the Cantabrian Region. This research has generated a substantial dataset of 30 valid new dates, adding to the pre-existing 61 dates achieved using the ultrafiltration protocol and adopted as truthful. A total of 91 high-quality radiocarbon dates (S3 Table) for the regional Mousterian, Châtelperronian, Aurignacian and Gravettian periods are now available (of the 128 attempted), enabling the construction of a much more precise chronological framework and spatio-temporal understanding of the transition and subsequent development of early Upper Paleolithic cultures in northern Iberia (Fig 5).

Detailed assessment of the sites that had previously been attributed until now to the Middle to Upper Paleolithic transition has found that not all of these can continue to be considered as belonging to this period. In addition, the dates show that for some of the sites, further work (including the acquisition of larger collections through future and controlled excavation) is required to review the industries and sedimentological units in order to arrive at definitive cultural attributions. Curatorial work may also be required to solve apparent problems of provenience among certain collections. Although this might challenge previous interpretations such as those concerning subsistence strategies during this period of bio-cultural transition in Northern Spain [64], it will contribute in the long term to a better understanding of this key era in a very important region for the study of the European Paleolithic.

The Bayesian model for the Mousterian in the Cantabrian Region shows that the dates obtained in this project are consistent with other, previously dated late Mousterian assemblages in Esquilleu, La Güelga, Morín, El Mirón and Arrillor [2,3,5,38] (S3 Table). The results allow us to confirm that the end of the Mousterian occurred between ~48-45ka cal BP (at 95.4% probability). This analysis highlights the earlier disappearance of the Mousterian in north-central Iberia in comparison with sites such as Romani and L’Arbreda in north-eastern Iberia or Pech de l’Azé in south-western France [3]. No new dates were obtained for the Châtelperronian; however, the Bayesian model confirms a brief overlap between the Châtelperronian and the Aurignacian in the Cantabrian region (despite the fact that this conclusion is highly dependent on a single date from the Proto-Aurignacian Level 16 of El Castillo). With these data, it is difficult to discuss the various hypothesis that have been proposed for the origins of Châtelperronian technocomplex (acculturation, autonomous development, stimulus diffusion), but at the regional scale an apparent gap between the late Mousterian and the Châtelperronian supports the absence of a local development and the potentially intrusive nature of the Châtelperronian. Alternatively, the presently available archaeological record could simply be missing sites dating to the intervening period of time, as yet undiscovered. The Aurignacian appears at ~43 ka cal BP, overlapping with the Châtelperronian at least within the error ranges of the few available dates. The Bayesian model for the Aurignacian shows two clusters of dates: ones falling between GI10 and GI8 and corresponding to the Proto and Early Aurignacian with an overlap, and a second group of dates, immediately before GI7 and extending until GS7, which correspond to the Evolved Aurignacian. The nature and causes of the spread of Aurignacian technologies and presumably modern humans remain critical subjects for ongoing paleoenvironmental and archaeological research.

The appearance of the Gravettian techno-complex was a relatively localized-process in the region and the dates obtained here increase our knowledge about its spatio-temporal development. In Aitzbitarte III a relatively quick cultural replacement of the Aurignacian by the Gravettian with Noailles burins is seen. This reinforces the hypothesis of a local origin for the Noaillian facies of the techno-complex so deeply rooted and long-lasting in the Basque Country [49]. The older Gravettian dates appear during GI7 in Aitzbitarte III, with more recent ones found further to the West in Asturias at Llonín and La Viña during GI6 and GI5, implying an East-West penetration of this culture into the region.

Despite the new evidence obtained here, establishing the end of the Mousterian, the time span of the Châtelperronian and the start of the Aurignacian in the Cantabrian region would still benefit from more radiocarbon dates in order to resolve still existing uncertainty. However, this possibility is dependent on the availability and high-quality excavation of new sites with the required degree of preservation to ensure the presence of collagen. However, while awaiting the appearance of new sites, the reliability of the potential overlap between the Châtelperronian and the Aurignacian could be increased by further sampling in the key levels 16 of El Castillo and 10 of Cueva Morin in the center of the Autonomous Community of Cantabria.

## Supporting information

S1 File Supporting information manuscript with a detailed description of each archaeological site sampled in this study. (DOCX)

S1 Table Radiocarbon AMS dates produced in this study. Collagen was extracted using the ultrafiltration protocol in all the samples. A contextual information of each archaeological level is provided, including a description of the lithic and bone artefacts and the archaeological context. Also, sample reference, animal species and skeletal element sampled including its taphonomic alterations is specified. For a reliable date, % yield should be >1%; %C >30% and C:N between 2.9 and 3.4 [65]. References to the original studies are cited and included below.
(DOCX)

S2 Table Results of the Order function comparing the PDF’s of the boundaries dating the start and the end of the archaeological industries from the Cantabrian region. Cells containing probabilities of >95% are coloured in green, 68–94% in orange and <68 in grey.
(DOCX)

S3 Table Radiocarbon accelerator dates from the Cantabrian region mentioned in this work. Only bone samples with ultrafiltration methods are included. ABA: charcoal fragment treated with a series of acid and base washes; ABOx-SC: charcoal treated with acid and base washes, followed by an oxidation stage and pre-combustion; UF AMS: collagen extracted using the ultrafiltration protocol.
(DOCX)

S4 Table Results of sensitivity test conducted on individual models. (DOCX)

S5 Table Results of sensitivity test conducted on regional models. (DOCX)

S1 Fig Radiocarbon dates from La Viña modelled in OxCal4.2 [41,43] against INTCAL13 [42]. (TIF)

S2 Fig Radiocarbon dates from Covalejos modelled in OxCal4.2 [41,43] against INTCAL13 [42]. (TIF)

S3 Fig Radiocarbon dates from El Cuco modelled in OxCal4.2 [41,43] against INTCAL13 [42]. (TIF)

S4 Fig Radiocarbon dates from Ekain modelled in OxCal4.2 [41,43] against INTCAL13 [42]. (TIF)

S5 Fig Radiocarbon dates from Amalda modelled in OxCal4.2 [41,43] against INTCAL13 [42]. (TIF)

S6 Fig Radiocarbon dates from Aitzbitarte III modelled in OxCal4.2 [41,43] against INTCAL13 [42]. (TIF)

S1 Code CQL individual codes. (DOCX)

S2 Code CQL regional codes. (DOCX)

## References

[1] Hublin J. The modern human colonization of western Eurasia: when and where? Quat Sci Rev. 2015; 118: 194–210. doi: 10.1016/j.quascirev.2014.08.011

[2] Maroto J, Vaquero M, Arrizabalaga A, Baena J, Baquedano E, Jordá J, et al Current issues in late Middle Palaeolithic chronology: New assessments from Northern Iberia. *Quat Internat*. 2012; 247:15–25. doi: 10.1016/j.quaint.2011.07.007

[3] Higham T, Douka K, Wood R, Ramsey CB, Brock F, Basell L, et al The timing and spatiotemporal patterning of Neanderthal disappearance. *Nature* 2014; 512: 306–309. doi: 10.1038/nature13621 25143113 10.1038/nature13621

[4] Wood RE, Arrizabalaga A, Camps M, Fallon S, Iriarte-Chiapusso MJ, Jones R, et al The chronology of the earliest Upper Palaeolithic in northern Iberia: New insights from l’Arbreda, Labeko Koba and La Viña. J Hum Evol. 2014; 69: 91–109. doi: 10.1016/j.jhevol.2013.12.017 24636733 10.1016/j.jhevol.2013.12.017

[5] Wood R, de Quirós FB, Maíllo-Fernández JM, Tejero JM, Neira A, Higham T. El Castillo (Cantabria, northern Iberia) and the Transitional Aurignacian: Using radiocarbon dating to assess site taphonomy. Quat Int. Forthcoming; doi: 10.1016/j.quaint.2016.03.005

[6] Higham T, Jacobi R, Bronk Ramsey C. AMS radiocarbon dating of ancient bone using ultrafiltration. Radiocarbon 2006; 48: 179–195. doi: 10.1017/S0033822200066388

[7] Higham T. European Middle and Upper Palaeolithic radiocarbon dates are often older than they look: problems with previous dates and some remedies. Antiquity 2011; 85 (327): 235–249. doi: 10.1017/S0003598X00067570

[8] Bar-Yosef O, Bordes JG. Who were the makers of the Châtelperronian culture? J Hum Evol 2010; 59, 586–593. http://dx.doi.org/10.1016/j.jhevol.2010.06.009 20692683 10.1016/j.jhevol.2010.06.009

[9] Benazzi S, Douka K, Fornai C, Bauer CC, Kullmer O, Svoboda J, et al Early dispersal of modern humans in Europe and implications for Neanderthal behaviour. Nature 2011; 479, 525–528. doi: 10.1038/nature10617 22048311 10.1038/nature10617

[10] Zilhão J, Banks W, d’Errico F, Gioia P. Analysis of Site Formation and Assemblage Integrity Does Not Support Attribution of the Uluzzian to Modern Humans at Grotta del Cavallo. Plos ONE 2015; 10(7): 131–181. doi: 10.1371/journal.pone.0131181 26154139 10.1371/journal.pone.0131181 PMC4495988

[11] Ruebens K. Regional behaviour among late Neanderthal groups in Western Europe: a comparative assessment of late Middle Palaeolithic bifacial tool variability. J Hum Evol. 2013; 65: 341–362. doi: 10.1016/j.jhevol.2013.06.009 23928352 10.1016/j.jhevol.2013.06.009

[12] Wood RE, Barroso-Ruiz C, Caparros M, Jordá JF, Galván Santos B, Higham TF. Radiocarbon dating casts doubt on the late chronology of the Middle to Upper Palaeolithic transition in southern Iberia. Proc Natl Acad Sci U S A 2013; 110: 2781–2786. doi: 10.1073/pnas.1207656110 23382220 10.1073/pnas.1207656110 PMC3581959

[13] Wood R. The contribution of new radiocarbon dating pre-treatment techniques to understanding the Middle to Upper Palaeolithic transition in Iberia. University of Oxford. PhD Thesis. 2011. https://ora.ox.ac.uk/objects/uuid:075d79c6-edb4-4f19-9e34-50a63e7b7fe0

[14] Altuna J, Baldeón A, Mariezkurrena K. editors. La Cueva de Amalda (Zestoa, País Vasco) Ocupaciones Paleolíticas y Postpaleolíticas. Donostia-San Sebastián: Sociedad de Estudios Vascos; 1990.

[15] González-Urquijo JE, Ibáñez-Estévez JJ. Abrigo de Axlor (Dima). Arkeoikuska: Investigación arqueológica; 2001 pp.90–93.

[16] Fortea J, de la Rasilla M, Rodríguez V. La cueva de Llonín (Llonín, Peñamellera Alta). Campañas de 1991 a 1994 Excavaciones Arqueológicas en Asturias 1987–1990, Oviedo: Servicio de Publicaciones del Principado de Asturias; 1992; 2: pp. 9–18.

[17] Fortea J, de la Rasilla M, Rodríguez V. La cueva de Llonín (Llonín, Peñamellera Alta). Campañas de 1991 a 1994 Excavaciones Arqueológicas en Asturias 1991–1994; 1995; 3: pp. 33–44.

[18] Fortea J, de la Rasilla M, Rodríguez V. La cueva de Llonín (Llonín, Peñamellera Alta). Campañas de 1995 a 1998 Excavaciones Arqueológicas en Asturias 1995–1998; 1999; 4: pp. 59–68.

[19] Fortea J, de la Rasilla M, Rodríguez V. L’art pariétal et la séquence archéologique paléolithique de la grotte de Llonín (Peñamellera Alta, Asturies, Espagne) Préhistoire, Art et Sociétés. BSPA 2004; LIX: 7–29.

[20] Fortea J, de la Rasilla M, Rodríguez V. La cueva de Llonín (Llonín, Peñamellera Alta). Campañas de 1999 a 2002 Excavaciones Arqueológicas en Asturias 1999–2002; 2007; 5: pp.77–86.

[21] Altuna J, Merino J. El Yacimiento Prehistórico de la Cueva de Ekain. San Sebastián: Sociedad de Estudios. Vascos; 1984.

[22] Altuna J, Mariezkurrena K, Rios-Garaizar J. Ocupaciones humanas en la cueva de Aitzbitarte III (Renteria, País Vasco) sector Entrada: 33.000–18.000 BP. Vitoria-Gasteiz: Eusko Jaurlaritzaren Argitalpen Zerbitzu Nagusia; 2011.

[23] Rasines del Río P. Geografía, estratigrafía y cronología de la cueva de Cobrante. Sautuola: Revista del Instituto de Prehistoria y Arqueología. 2009; 15: 43–47.

[24] Muñoz Fernández E, Serna Gancedo A. Los niveles solutrenses de la Cueva del Ruso I (Igollo de Camargo, Cantabria). Sautuola: Revista del Instituto de Prehistoria y Arqueología. 1999; 6: 161–176.

[25] Echegaray JG. Cueva del Otero. Excavaciones Arqueológicas en España, 53. Madrid: Ministerio de Educación Nacional Dirección General de Bellas Artes, Servicio Nacional de Excavaciones Arqueológicas; 1966.

[26] Gonzalez Echegaray J. Freeman LG. Cueva Morín: Excavaciones 1966–1968. Santander: Patronato de Cuevas Prehistóricas; 1971.

[27] Sanguino González J, Montes Barquín R. Nuevos datos para el conocimiento del Paleolítico Medio en el centro de la Región Cantábrica: La Cueva de Covalejos. Santander: Museo de Altamira Monografías. 2005; 20: 10–38.

[28] Barandiarán JM. Bolinkoba y otros yacimientos paleolíticos en la sierra de Amboto (Vizcaya) Cuadernos de historia primitiva 5, 2. Madrid: Seminario de Historia Primitiva del Hombre; 1950.

[29] Iriarte-Chiapusso MJ, Arrizabalaga A. El yacimiento arqueológico de Bolinkoba (Abadiño, Bizkaia). Crónica de las investigaciones en la cavidad. Secuencia estratigráfica y cronología numérica, In: Iriarte-Chiapusso MJ, Arrizabalaga A, editors. Bolinkoba (Abadiño) y su Yacimiento Arqueológico: Arqueología de La Arqueología Para La Puesta En Valor de Su Depósito, a La Luz de Las Excavaciones Antiguas Y Recientes. Kobie Serie BAI 6. Diputación Foral de Bizkaia, Bilbao; 2015: pp. 5–88.

[30] Muñoz Fernández E, Montes Barquín R. editors. Intervenciones arqueológicas en Castro Urdiales, tomo III. Arqueología y arte rupestre paleolítico en las cavidades de El Cuco o Sobera y La Lastrilla. Castro Urdiales: Excmo. Ayuntamiento de Castro Urdiales. Concejalía de Medio Ambiente y Patrimonio Arqueológico; 2007.

[31] Gutierrez-Zugasti I, Ríos-Garaizar J, Marín-Arroyo AB, Rasines P, Maroto J, Jones J, Bailey G, Richards M. Forthcoming. A chrono-cultural reassessment of the levels VI-XIV from El Cuco rock-shelter: a new sequence for the Late Middle Paleolithic in the Cantabrian Region (northern Iberia). Quat Int https://doi.org/10.1016/j.quaint.2017.06.059

[32] Fortea J. Abrigo de La Viña. Informe de las campañas 1980–1986. Excavaciones Arqueológicas en Asturias (1983–1986). 1990; 1: 55–68.

[33] Fortea J. Abrigo de La Viña. Informe de las campañas 1987–1990. Excavaciones Arqueológicas en Asturias (1987–1990). 1992; 2: 19–28.

[34] Fortea J. Abrigo de La Viña. Informe y primera valoración de las campañas 1991 a 1994. Excavaciones Arqueológicas en Asturias (1991–1994). 1995; 3: 19–32.

[35] Fortea J. Le Paléolithique supérieur en Espagne, Galice et Asturies, 1991–1995. En M. Otte (dir.), Le paléolithique supérieur Européen: bilan quinquennal 1991–1996. ERAUL 76. Liège: Université de Liége; 1996. pp. 329–339.

[36] Fortea J. 1999 Abrigo de La Viña. Informe y primera valoración de las campañas de 1995 a 1998. Excavaciones Arqueológicas en Asturias (1995–1998), 4: 31–41.

[37] Bradtmöller M. The Gravettian occupation of Level 4 Cueva Morín and its regional context. Munibe. 2015; 66: 23–52.

[38] Straus LG, González Morales MR. El Mirón Cave (Ramales, Cantabria, Spain) date list V: Middle Paleolithic and Lower Magdalenian. Radiocarbon. 2016; 58(4): 943–945. doi: 10.1017/RDC.2016.84

[39] Brock F, Higham T, Ramsey CB. Pre-screening techniques for identification of samples suitable for radiocarbon dating of poorly preserved bones. J Archaeol Sci. 2010; 37 (4): 855–865. doi: 10.1016/j.jas.2009.11.015

[40] Bronk Ramsey C, Higham T, Bowles A, Hedges R. Improvements to the pre-treatment of bone at Oxford. Radiocarbon. 2004; 46 (1): 155–163. doi: 10.1017/S0033822200039473

[41] Bronk Ramsey C. Bayesian analysis of radiocarbon dates. Radiocarbon. 2009; 51(1): 337–360. doi: 10.1017/S0033822200033865

[42] Reimer PJ, Bard E, Bayliss A, Beck JW, Blackwell PG, Bronk Ramsey C. et al IntCal13 and Marine13 radiocarbon age calibration curves 0–50,000 years cal BP. Radiocarbon. 2013; 55 (4): 1869–1887. doi: 10.2458/azu_js_rc.55.16947

[43] Bronk Ramsey CB. Dealing with outliers and offsets in radiocarbon dating. Radiocarbon. 2009; 51 (3): 1023–1045. doi: 10.1017/S0033822200034093

[44] Andersen KK, Svensson A, Johnsen SJ, Rasmussen SO, Bigler M, Röthlisberger R, et al The Greenland ice core chronology 2005, 15–42 ka. Part 1: constructing the time scale. Quat Sci Rev. 2006; 25: 3246–3257. doi: 10.1016/j.quascirev.2006.08.002

[45] Svensson A, Andersen KK, Bigler M, Clausen HB, Dahl-Jensen D, Davies SM, et al The Greenland ice core chronology 2005, 15–42 ka. Part 2: comparison to other records. Quat Sci Rev. 2006; 25: 3258–3267. doi: 10.1016/j.quascirev.2006.08.003

[46] DeNiro MJ. Postmortem preservation and alteration of in vivo bone collagen isotope ratios in relation to palaeodietary reconstruction. Nature. 1985; 317: 806–809.

[47] Bradtmöller M. Höhlenlager des Gravettien—Muster jungpaläo¬lithischer Höhlennutzung am Beispiel des Gravettien Nordspaniens. Hamburg: Verlag Dr. Kovač; 2014.

[48] Altuna J, Mariezkurrena K, de la Peña P, Rios-Garaizar J. Los niveles gravetienses de la cueva de Aitzbitarte III (Gipuzkoa). Industrias y faunas asociadas In: de las Heras C., Lasheras J.A., Arrizabalaga Á., de la Rasilla M. editors. Pensando El Gravetiense: Nuevos Datos Para La Región Cantábrica En Su Contexto Peninsular Y Pirenaico. Monografías Del Museo Nacional Y Centro de Investigación de Altamira, 23. Madrid: Ministerio de Educación, Cultura; 2013 pp. 184–204.

[49] Rios-Garaizar J, de la Peña P, Maillo-Fernández JM, 2013 El final del Auriñaciense y el comienzo del Gravetiense en la región cantábrica: una visión tecno-tipológica In: de las Heras C, Lasheras JA, Arrizabalaga Á, de la Rasilla M. editors. Pensando El Gravetiense: Nuevos Datos Para La Región Cantábrica En Su Contexto Peninsular Y Pirenaico. Monografías Del Museo Nacional Y Centro de Investigación de Altamira, N.o 23. Madrid; Ministerio de Educación, Cultura, 2013; pp. 369–382.

[50] Bernaldo de Quirós F. Los inicios del Paleolítico Superior Cantábrico. Santander: Monografías del Museo y Centro de estudios de Altamira 8; 1982.

[51] Muñoz Fernández E, Santamaría Santamaría S. Análisis de la industria lítica de la cueva de Cobrante. Sautuola 2009; 15: 145–189.

[52] Muñoz Fernández E. Excavaciones arqueológicas en la Cueva del Ruso I. Avance preliminar. Arquenas. 1991: 61–157.

[53] Barandiarán Maestu I. Paleomesolítico del Pirineo Occidental. Bases para la sistematización tipológica del instrumental óseo paleolítico Monografías Arqueológicas. Zaragoza: Universidad de Zaragoza; 1967.

[54] Altuna J. Situación y descripción de la cueva de Amalda. Historia de las excavaciones. Descripción del relleno. Estructuras en el yacimiento. Dataciones de radiocarbono. Otros yacimientos del valle In: Altuna J, Baldeón A, Mariezkurrena K. editors. La Cueva de Amalda (Zestoa, País Vasco). Ocupaciones Paleolíticas Y Postpaleolíticas. Sociedad de Estudios Vascos, Donostia-San Sebastián, 1990 pp. 9–31.

[55] Rios-Garaizar J. Organización económica de las sociedades Neandertales: el caso del nivel VII de Amalda (Zestoa, Gipuzkoa). Zephyrus. 2010; LXV, 15–37.

[56] Rios-Garaizar J, de la Peña P, San Emeterio A. Estudio de las industrias líticas y óseas de la cueva de Aitzbitarte III (Zona de la entrada) In: Altuna J., Mariezkurrena K., Rios-Garaizar J. editors. Ocupaciones Humanas En La Cueva de Aitzbitarte III (Renteria, País Vasco) Sector Entrada: 33.000–18.000 BP. Vitoria-Gasteiz: Eusko Jaurlaritzaren Argitalpen Zerbitzu Nagusia, 2011 pp. 81–351.

[57] Rios-Garaizar J, Arrizabalaga Á, Villaluenga A. Haltes de chasse du Châtelperronien de la Péninsule Ibérique. Labeko Koba et Ekain (Pays Basque Péninsulaire). L’Anthropologie 2012; 116: 532–549. doi: 10.1016/j.anthro.2012.10.001

[58] Camps M, Higham T. Chronology of the Middle to Upper Palaeolithic transition at Abric Romaní, Catalunya. J Hum Evol. 2012; 62 (1): 89–103. doi: 10.1016/j.jhevol.2011.10.010 22137586 10.1016/j.jhevol.2011.10.010

[59] Zilhão J. Chronostratigraphy of the Middle-to-Upper Paleolithic Transition in the Iberian Peninsula. Pyrenae 2006; 37(1): 7–84.

[60] Lowe J, Barton N, Blockley S, Bronk Ramsey C, Cullen V, Davies W, et al Volcanic ash layers illuminate the resilience of Neanderthals and early modern humans to natural hazards. Proc Natl Acad Sci U S A 2012; 109 (34): 13532–13537. doi: 10.1073/pnas.1204579109 22826222 10.1073/pnas.1204579109 PMC3427068

[61] Fedele FG, Giaccio B, Hajdas I. Timescales and cultural process at 40,000 BP in the light of the Campanian Ignimbrite eruption, Western Eurasia. J Hum Evol 2008; 55: 834–857. doi: 10.1016/j.jhevol.2008.08.012 18922561 10.1016/j.jhevol.2008.08.012

[62] Golovanova LV, Borisovich Doronichev V, Elansia Cleghorn N, Koulkova M, Sapelko T, Shackley MS. Significance of ecological factors in the Middle to Upper Paleolithic Transition. Current Anthropolology 2010; 51: 655–691. doi: 10.1086/656185

[63] Maíllo-Fernández JM, Bernaldo de Quirós F. Archaic Aurignacian in El Castillo cave (Spain): technology and typology composition. Anthropologie. 2010; 114 (1): 1–25. doi: 10.1016/j.anthro.2010.01.001

[64] Yravedra J. New Contributions on Subsistence Practices during the Middle-Upper Paleolithic in Northern Spain In: Clark J, Speth J. (Eds). Zooarchaeology and Modern Human Origins. Vertebrate Paleobiology and Paleoanthropology book series. Springer; 2013 pp 77–95.

[65] van Klinken GJ. Bone Collagen Quality Indicators for Palaeodietary and Radiocarbon Measurements. J Archaeol Sci 1999; 26(6), 687–695. doi: 10.1006/jasc.1998.0385

