# Correction: Chronological reassessment of the Middle to Upper Paleolithic transition and Early Upper Paleolithic cultures in Cantabrian Spain

**DOI:** 10.1371/journal.pone.0199954

**Published:** 2018-06-26

**Authors:** Ana B. Marín-Arroyo, Joseba Rios-Garaizar, Lawrence G. Straus, Jennifer R. Jones, Marco de la Rasilla, Manuel R. González Morales, Michael Richards, Jesús Altuna, Koro Mariezkurrena, David Ocio

There are errors in the headings of the last three columns before the references in S1 Table. The sixteenth column should be d13C (‰ VPDB), the seventeenth column should be d15N (‰ AIR), and the eighteenth column should be % collagen yield. Please see the corrected S1 Table here.

## Supporting information

S1 Table Radiocarbon AMS dates produced in this study. Collagen was extracted using the ultrafiltration protocol in all the samples. A contextual information of each archaeological level is provided, including a description of the lithic and bone artefacts and the archaeological context. Also, sample reference, animal species and skeletal element sampled including its taphonomic alterations is specified. For a reliable date, % yield should be >1%; %C >30% and C:N between 2.9 and 3.4 [65]. References to the original studies are cited and included below.
(DOCX)
