# Supplementary material for: Chronological reassessment of the Middle to Upper Paleolithic transition and Early Upper Paleolithic cultures in Cantabrian Spain
Source: PLoS One. 2018 Apr 18;13(4):e0194708. doi: 10.1371/journal.pone.0194708 (PMC5905894; doi:10.1371/journal.pone.0194708)
Supplement: S1 File — (DOCX) [file pone.0194708.s001.docx]

**SUPPLEMENTARY INFORMATION**

*Archaeological sites*

*Asturias*

**La Viña**, located in La Manzaneda near Oviedo (central Asturias), was excavated between 1980-1996 revealing a stratigraphy ranging from the Mousterian to the Magdalenian [1-5]. Lithic assemblages from Mousterian, Aurignacian and Gravettian levels have recently been studied [6-7]. Recent radiocarbon dating at the site, using ultrafiltration techniques, revealed that Mousterian Level XIII-basal was beyond the radiocarbon limit, with dates of >62,000 (OxA-19196) and >59,300 (OxA-19144) [8]. This was followed by a hiatus in the stratigraphy caused by an erosional event. Level XIII-inferior, originally attributed to the Proto-Aurignacian with a conventional date of 36,500±750 (Ly-6390) on charcoal [5,6]. This level was interpreted as one of the oldest manifestations of the Aurignacian in the region, supporting the idea of a rapid occupation of the Cantabrian coastal strip by modern humans [9]. Level XIII yielded an AMS ^14^C date on charcoal of 31,860±680 (GifA-95463), and the recently obtained ultrafiltration dates have produced similar results, 31,160±360 (OxA-21705) and 30,650±230 (OxA-21845), which are consistent with an attribution to the Early Aurignacian. Level XII is attributed to the Evolved Aurignacian, with radiocarbon assays yielding similar dates to those of Level XIII: 31,600±400 (OxA-21678) and 31,500±400 (OxA-21689). Level XI also attributed to the Evolved Aurignacian, yielded two similar dates of 30,600±370 (OxA-21687) and 30,130±170 (OxA-19195) and another slightly younger one of 27,900±280 (OxA-X-2290-19), which may indicate that not all contaminants had been removed or that there had been some stratigraphic mixing, and a very young (Solutrean) date of 20,820±130 (OxA-21686) which was interpreted as a specimen that fallen from the section between excavation seasons [8]. Up to now, the only available date for the entire group of four Gravettian Levels at the site (X-VII) had been obtained from Level IX, with a result of 24,640±190 (OxA-21688). The faunal collection, curated at the University of Oviedo, is currently under study by one of us (ABMA). In this project, seven dates were attempted: two from Level X, one from Level IX, two from Level VIII and two dates from Level VII.

Dating results from **La Viña** are consistent with the stratigraphic order of cultural periods at the site. Aside from one date that failed due to poor preservation, the seven dates attempted provided accurate results that were consistent with the stratigraphy at the site. All the samples dated were attributed to the Gravettian technocomplex. The two samples dated from Level X yielded similar results: 28,560±300 (OxA-32550) and 28,940±310 (OxA-32551). Level IX provided a date of 28,360±290 (OxA-34929). One date of Level VIII failed due to low collagen yields (P-38643) and another gave a date of 25,000±200 (OxA-32514). Finally, Level VII provided two dates of 23,960±180 (OxA-32515) and 23,930±180 (OxA-32516).

We built a Bayesian model for the complete sequence, including previous ultrafiltered dates and our new dates. We excluded from the model the two “young” dates from Level XI (OxA-21686 and OxA-X-2290-19) suggested to be too young as probable outliers because of the incomplete removal of contaminants [8]. We can confirm that both dates are too young for their stratigraphic position. The new sequence is shown in S1 Fig. Following previous results for the Aurignacian [8], the boundary between it and the start of the Gravettian is 34,420-32,790 cal BP and the Gravettian ending boundary is 29,182-27,872 cal BP (at 95.4% probability). The date range of Level X is 34,034-32,194 cal BP (95,4% probability), for Level IX it is 33,012-29,750, for Level VIII, 31,518-28,142 cal BP and for Level VII, 28,880-26,100 cal BP.

If we compare dated levels with the chronological Bayesian model for the Gravettian in the entire Cantabrian Region (Fig 4; S2 Table), Levels X and IX could represent an early phase of the Gravettian, contemporaneous with Amalda VI, Llonín V, Aitzbitarte III Level IV, but younger than the Early Gravettian of Aitzbitarte III Levels Va and Vb upper. The date from Level VIII of La Viña might correspond with a middle phase of the Gravettian and contemporaneous with Bolinkona Level VI/F. Finally, Level VII dates were very consistent between them and might correspond with a later phase of the Gravettian in turn contemporaneous with Morín Level 4 [10].

The environmental evidence suggests that the Aurignacian deposits were accumulated after GS8, when tentatively compared to the NGRIP GICC05 record, while the Gravettian levels spanned interstadials GI6 and GI3.

The site of **Llonín**, in Peñamellera Alta (eastern Asturias), was excavated in a series of campaigns between 1987-2002. Trenches were dug in four different sectors of the cave called “Galería”, “Vestibulo”, “Cono Anterior”, “Cono Posterior”, revealing occupations from Middle Paleolithic to Azilian. In “Galería”, Level V was attributed to the Gravettian, and Level VI to the Middle Paleolithic [11-16]. Also, excavation of Levels VI-VIII from “Cono Posterior” produced Middle Paleolithic (Level VIII) and Initial Upper Paleolithic (Level VI) materials, but typo-technological studies suggest prehistoric contamination from the other levels mentioned in level VII [16]. The dates obtained from faunal remains yielded overly recent results, suggesting some sort of disturbance [17]. Level VI from this sector was dated to 22,090±290 (Ua-23954) from an ibex tooth, Level VII to 34,100±1240 (Ua-23955) and Level VIII to 29,995±1045 (Ua-23956), the last ones dated from chamois teeth [18]. We attempted two dates, one each from Levels V and VI. The faunal collection, curated at the University of Oviedo, is currently under study. The sample from Mousterian Level VI (Galería) failed due to a high C:N value (P-38649). The two samples dated from Level V in Galería, attributed to the Gravettian, yielded two very different results: one of 28,390±350 (OxA-X-2640-48) which would be coherent with an Early Gravettian attribution, contemporaneous with La Viña Levels X-IX, Amalda VI and Aitzbitarte III Level Va and IV within the chronological model for the Gravettian in the Cantabrian region (Fig 4). Another date for Level V of 20,040±120 (OxA-32517) which could correspond to an early Solutrean or a very late phase of the Gravettian, younger than Morín Level 4 [10] and similar to the AMS dates of Level III from Aitzbitarte III [19]. These results suggest either a much longer formation for Level V (more than 8,000 years for only 9 cm of stratigraphy), although we cannot dismiss the possibility of some disturbance or admixture with overlying Solutrean Level IV which remains undated. No modelling was undertaken for this site.

*Cantabria*

**Covalejos** in Piélagos, Cantabria, has a sequence dating from the Early Middle Paleolithic to the Aurignacian. The site was discovered by E. de la Pedraja in 1872, and excavations at the site continued until 1879. Recent excavations at the site were carried out between 1997-1999 and again in 2002 [20]. Materials recovered during the latter work were dated in this project. The faunal collections are curated at the Museum of Prehistory and Archaeology of Cantabria. Levels B/2 and C/3 are considered to be Early Aurignacian and Archaic or Proto-Aurignacian respectively, while Levels D/4 to M/11 were attributed to the Mousterian, and Level Q/15 potentially belongs to a Late Acheulean or Early Middle Paleolithic. Specifically related to the Middle-Upper Paleolithic transitional levels, AMS radiocarbon dates from Levels B, C and D were undertaken [20]. Early Aurignacian Level B/2 was dated to 30,380±250–250 (GrA-22443), two assays from Archaic Aurignacian Level C/3 provided both a younger date of 32,840+280–250 BP (GrA-24200) and an older one of 37,940+400–350 BP (GrA-33877), all conducted on animal teeth. Mousterian Level D/4 provided two dates of 41,640+650–650 BP and 40,650+2300–2,300 BP. Recently, radiocarbon dates on samples performed on teeth were obtained for Archaic Aurignacian Level C/3 and Mousterian Levels D/4, I/7 and J/8. From the top to the bottom of the Mousterian sequence, the Level J/8 date was beyond the limit of the radiocarbon method, with a result of >45,000 (GrA-33812); Level I/7 gave a date of 30,860±340–300 BP(GrA-33822), which is too young for its cultural attribution and also younger than the dates obtained from Archaic Aurignacian Level C/3. Level (C/3) yielded a date of 37,940±400–350 BP(GrA-33877), and Level D/4 provided an even older date of 43,050+750–550 BP (GrA-33811)[21]. In this project four dates were attempted, two for each Aurignacian Level: B/2 and C/3, respectively.

The four dates are internally consistent. Previous dates supported a stratigraphic and cultural succession of Levels C/3 (Proto-Aurignacian) and Level B/2 (Early Aurignacian). The new ultrafiltered dates suggest that both Levels were almost contemporaneous and older than previously thought, probably because of the removal of contaminants from the latter samples. Level B/2 has dates of 34,350±600 (OxA-32549) and 35,150±650 (OxA- 32513), whilst Level C/3 has dates of 34,850±600 (OxA-32512) and 35,250±700 (OxA-32511). These results are slightly younger than key Proto-Aurignacian levels in the region, namely Labeko Koba Levels VII and VI, dated between 35,100±600 (OxA-21778) and 36,850±800 (OxA-21766) [8] and El Castillo Level 16 dated to 38,600 ± 1,000 (OxA-22200) [22]. Given that the material culture does not include any clearly distinctive Proto-Aurignacian artifacts for Level C/3 and that six split-base points were recovered from Level B/2, both Levels could correspond to initial phases of the Early Aurignacian, contemporaneous with Levels 13 and 14 of Abri Pataud in Dordogne (SW France) [23]. However, the possibility of a quick replacement of the Proto-Aurignacian by the Early Aurignacian, as proposed for Les Cottés [24], should not be dismissed here. Further analyses of the lithics, bone tools and ornaments are needed to clarify this issue.

The Bayesian age model for the Aurignacian sequence in Covalejos is shown in S2 Fig. The date range for Aurignacian Level C/3 is 40,984-38,754 cal BP and for Level B/2 is 40,208-38,000 cal BP (95.4% probability). The boundary between these Levels is 40,288-38,686 cal BP. Both Levels were accumulated between 40,492-38,450 cal BP (68.2% probability) and 41,850-37,084 cal BP (95.4% probability). When compared to the NGRIP GICC05 record, both Aurignacian Levels were accumulated during GS9, predating GI8 and coinciding with Heinrich Event 4.

**El Ruso I**, located in Igollo de Camargo, was discovered in the 1950s by J. Quintana, after which several test pits were dug by Quintana and the Sautuola Seminar of the Santander Museum. The most extensive, salvage excavations at the site were undertaken in 1984 in the “Sala del Yacimiento” sector, where a sequence of seven archaeological levels was defined [25]. Level IVb was initially attributed, with reservations to the “Cantabrian Solutrean”, and later to the Evolved Aurignacian based on a single radiocarbon date of 27,620±180 (Beta-70812) [26]. The authors identified some problems during excavation in separating Level IVb from both the underlying Level V (Mousterian) and the overlying Level IVa (Upper Solutrean) [27]. Also, a test pit excavated from Level IVb into Level Va was identified as a source of possible mixing. The small lithic assemblage from Level IVb is not very diagnostic, but the presence of a flat-nosed end-scraper and a double carinated end-scraper was mentioned. Lithic industry from Level V was attributed to the Mousterian, but after dating it yielded a very recent age of 30,200±1,360 (Beta-70813), which was discarded by the excavators. Some authors have questioned the integrity of this level [28] and others have argued that the presence of some atypical lithic tools such as rabots, burins, an Abri Audi knife, a decorated bone and a perforated *Littorina obtusata* shell indicates more likely an attribution to the Initial Upper Paleolithic [29]. Here we focused on attempting two dates from Level IVb. One of the dates failed due to low collagen yields (P-38631) and the other one gave a result of 28,510±300 (OxA-32507), which is consistent with the previous date for this level. Considering that no clearly diagnostic material was found in this level and that the dates fall into the range of Early Gravettian in the Cantabrian Region, it is difficult to propose any cultural attribution to this Level, but there is no argument to continue considering it as Evolved Aurignacian. No modelling was undertaken for this site and the date was not included within the chronological model either for the Aurignacian or Gravettian in the region.

**Morín** cave has long been a key site for investigating the Middle to Upper Paleolithic transition in the Cantabrian Region. It is located in Villaescusa (Cantabria) and was discovered by Obermaier and Wernert in the early 1900s. It was extensively excavated by González Echegaray and Freeman between 1966 and 1968 [30]. In 2008, a brief, new excavation campaign, directed by González Urquijo and Weniger, was undertaken. Morín contains an archaeological sequence spanning from the Mousterian to the Azilian, including Late Mousterian (Levels 11 and 12), Châtelperronian (10), and Proto-Aurignacian (8,9) levels, although the reliability of the sequence has been contested [30]. Dating of these levels has been attempted by several scholars producing diverse results concerning the chronology of the site [32-34]. New radiocarbon assays on charcoal samples from Levels 8-11 were run to address this problem using ABA pre-treatment and AMS in different laboratories [21]. Late Mousterian Level 11 was initially dated, using AMS, to 42,000±730 (GifA-96264) [34]; two new dates are similar: an ABA date of 41,800±450 (OxA-19083) and 43,600±600 (OxA-19459) using ABOX-SC protocol. Level 10, attributed to the Châtelperronian, was dated using conventional radiocarbon techniques to 27,800±580 BP (SI-951) and 35,875±6780 BP (SI-951A) [32,34]. New dating, using AMS, yielded a result of 29,380+260/-240 (GrA-33823) [21], which is interpreted as being too young for a Châtelperronian assemblage. Level 9, attributed to the Archaic Aurignacian, has a single AMS date of 33,430±250-230 (GrA-33891). Two conventional ^14^C dates were obtained previously for Level 8, also interpreted as Archaic Aurignacian: 27,630±540 (SI-952) and 27,360±740 (SI-952A). AMS dates were more recently produced for Level 8: 36,590±770 (GIFA-96263) and 40,060±350 (OxA-19084). The latter date seems too old for the Proto-Aurignacian and is inconsistent with the dates obtained from Levels 10 and 9 [21]. No new dates were attempted for Levels above Level 8, although there are conventional ^14^C dates for the Early Aurignacian (Level 7): 27,565±865 and 26,105±1535 (SI-955A). These two dates on Level 7 fall into the age range of the Gravettian, and another date of 31,740±865 (SI-954) might be more consistent with the Aurignacian attribution of Level 7. The conventional ^14^C date for Level 5s (Gravettian), gave a younger date of 20,120±350 (SI-953), while the recent AMS dates on bone were 28,700±400 (Poz-36383) and 28,580±240 (Poz-36973) [10]. For Level 4, also interpreted as Gravettian, a conventional ^14^C date on humus of 15,240±1749 (WSU-500) was obtained. More recently AMS dates on two bones from Level 4 gave results of 18,270±130 (Poz-36374) and 22,420±200 (Poz-36375) [35], and ultrafiltration dates on two other bones from the same Level provide older dates and more consistent results of 23,640±190 (Poz-66758) and 23,790±190 (Poz-66759) [35]. Only one date from Level 7c, classified as Early Aurignacian, was attempted here and it failed due to poor collagen preservation (P-38636), and thus, no additional information about site chronology can be provided. For Morín, as commented by various researchers, it is possible that frequent moving of the collections over the years among multiple temporary facilities of the Santander Museum of Prehistory and Archaeology, and a flood at one of them, might have caused provenience and preservation problems with the material.

**Cobrante**, located in Valle de Aras (Cantabria), was discovered in the second half of the 19th century by M. Sanz de Sautuola, the discoverer of Altamira. A few test pits were excavated at the site over time, revealing Paleolithic and post-Paleolithic human occupations. In 1966, several wall-engravings were discovered and later the cave was declared as a National Historical-artistic monument. In 1998, a 2m^2^ test pit was made 30 m inside the cave [36]. Here a 1.2 m-deep sequence was excavated, and seven stratigraphic units were differentiated. Level 5 has been attributed to the Aurignacian, despite its lack of characteristic material culture [37]. This attribution was based solely on a single AMS date of 30,480 ± 250 (GrA-22441). Level 6 was interpreted as Archaic Aurignacian (a.k.a. Proto-Aurignacian) because of the presence of characteristic lithic tools, such as Dufour bladelets, large “Aurignacian” blades and carinated end-scrapers, which appeared alongside Mousterian-looking tools [37]. This level was dated to 33,320±310 (GrA-22442). Level 7 was also interpreted as Proto-Aurignacian, but with no date, and the lithic assemblage yielded few characteristic tools (just a single carinated end-scraper and two atypical Dufour bladelets).

Two dates each from Levels 5 and 6 each were attempted. Level 6, originally classified as Proto-Aurignacian, produced two very distinct dates: 35,150±650 (OxA-32505) and >44,800 (OxA-32506). This incoherence could reflect a problem with curation, as the sample for the latter date, stored with material from Level 6, had no individual marked label. Thus, there might be a problem of admixture or level misidentification, unnoticed during excavation. The relevant presence of typical Mousterian tools in Level 6 [37] would support this hypothesis of admixture, but further analysis needs to be done. What seems clear in Cobrante is that there are elements in Level 6 possibly corresponding with a Proto-Aurignacian occupation, but up to now these results should be taken with caution. Also, if the hypothesis of a Mousterian presence also in Level 6 is correct, then the interpretation of Level 7 as Proto-Aurignacian should also be revisited, especially considering that no clearly diagnostic material was recovered from this level. For Level 5 the new assays offer a different panorama, with two dates clearly clustered around 18,500 BP. These results suggest that the level was contemporaneous with the regional Solutrean from such sites as Las Caldas, La Riera, Altamira and El Mirón caves [38-41] instead of Aurignacian. A closer examination of lithic tools from Level 5 reveals the presence of at least one item with partial invasive (Solutrean) retouch [37]. Further analyses of this level are needed, but to date there is no argument for supporting an Aurignacian attribution. No modelling was undertaken for this site and the date from Level 6 was not included within the chronological model for the Aurignacian in the Cantabrian region.

The site of **El Otero** in Secadura (Voto), eastern Cantabria, was excavated in 1963, focussing on two different sectors (Sala I and Sala II) joined by a trench [42]. The stratigraphic sequence in both sectors were more or less equivalent. In Sala I nine Levels were identified: from top to bottom, Levels I-IX. Level IX yielded few artifacts, including a denticulated flake, attributed (optimistically) to the Mousterian. Level VIII also produced a small lithic collection, with 18 retouched tools, including carinated end-scrapers (bladelet cores) and one Aurignacian blade, alongside some tools on flakes, including a typical Mousterian point. These apparently mixed features determined its attribution to a supposed “Aurignaco-Mousterian” (a combination of Mousterian and Aurignacian features), a general concept later disproved [30,43]. Above these two levels formed a thick speleothem (Level VII), and above it, there were three Aurignacian Levels (Levels VI, V, IV). Level VI was characterized by an Upper Paleolithic industry, with end-scrapers, burins, Aurignacian blades and some tools on flake. Among the end-scrapers, some carinated and nosed examples (functionally probably bladelet cores) were identified. Some bone tools were also recovered, including a flat bone point. These features promoted its attribution to the “Aurignacian III” (Evolved Aurignacian). Level V was recognized in both sectors (Sala I and Sala II) and had a small lithic collection, including some atypical end-scrapers, burins on truncation, borers and raclettes and two perforated red deer teeth. This level was attributed to the “Aurignacian IV” (Evolved Aurignacian). Finally, Level IV was the richest Aurignacian level with numerous end-scrapers on blades and many Aurignacian blades, but without clear carinated elements. Burins, truncations and retouched bladelets are less abundant than in the earlier levels [42,44]. There are also some perforated deer, ibex and fox teeth and some non-characteristic bone tools. This level was originally attributed to the “Aurignacian V” (a Last Glacial Maximum industry that in France supposedly preceded the Solutrean [45] but recently it was assigned to the Evolved Aurignacian [44]. Until now, no radiocarbon dating had been undertaken in El Otero, and doing so was considered important for clarifying its stratigraphic sequence, particularly for Levels VI, V and IV, supposedly corresponding to the Middle to Upper Paleolithic Transition.

Four dates obtained for **El Otero** Levels VI, V and IV, which were originally classified as Aurignacian but never previously dated, yielded very young results for such a cultural attribution and show significant stratigraphic inconsistency. Level VI gave a result of 12,415±55 (OxA-32585), two dates in Level V are 12,340±55 (OxA-32509) and 10,585±50 (OxA-32510) and a date in Level IV is 15,990±80 (OxA-32508). All these results fall into the range of the Late Upper Paleolithic (Magdalenian-Azilian), eliminating attribution of these Levels to the Aurignacian, despite the presence of apparently characteristic artifacts [42,44,46]. This result urges caution in the dating of levels solely using artifacts that are not temporally diagnostic.

**El Cuco,** located in Castro Urdiales (eastern Cantabria), is a small cave with a wide rock-shelter for its the entrance. The site was known for its rock-art manifestations since 1966. In 2005, two test pits of 1m^2^ each were excavated by P. Rasines [47]. The first one (Sector A) situated in the cave entrance revealed a sequence of 13 archaeological levels covering the period from the Gravettian to the Holocene (Levels D-M). The second test pit (Sector B) within the rock shelter, revealed 14 archaeological Levels (I-XIV). In sector B, Levels VII-XIV were initially attributed to the Evolved Aurignacian. This attribution was based on a single date of 30,020 ±160 -150 uncal BP (GrA-32436) from Level XIII obtained on charred ungulate bone bioapatite [21]. Recently this part of the sequence has been reanalysed [48]. A single assay from Level X, made on bone collagen of an ungulate, produced a result of 14,550±80 (UGAMS-9076) unreliable due to the low collagen yield, and another one on a red deer bone failed. Given the problems with bone collagen preservation at the site, several limpets (*Patella vulgata*) from Levels X, XII and XIII were dated. The obtained results were much older than expected: for Level X: 42,350 ± 700 (OxA-27196) and 46,200 ± 650 (OxA-27115); for Level XII: >43,500 (Beta -382681) and for Level XIII: 46,400 ± 800 (OxA-30851). The re-analysis of the lithic assemblages from Levels VII to XIV revealed that there are no typical Aurignacian tools and that these assemblages are typologically and technologically attributable to the Middle Paleolithic, with special incidence of Levallois technology [48]. On the other hand, the upper part of the sequence (Levels V-III) was initially attributed to the Gravettian [47,49] after obtaining a single AMS date on charred bone bioapatite of 23,400 ± 210 uncal BP (GrA-32097). In this project, two bone samples from Levels III and one from Level Vb were submitted for dating. One of the two assays attempted for Level III failed due to low collagen yield (P-38623) and the other gave a result of 35,050±650 (OxA-32502). The date for Level Vb is 49,500±3,900 (OxA-X-2640-11).

A Bayesian age model for the site is shown in S3 Fig. The date range for Level VB is 55,276-42,816 cal BP and for Level III is 45,498-32,544 cal BP (95.4% probability). In the model, a hiatus between these levels was considered, as there are no dates for Level IV. When compared to the NGRIP GICC05 record, we suggest that Level Vb was accumulated during GS13 predating GI12.

The date of Level III falls at the temporal boundary between regional Proto-Aurignacian and Early Aurignacian, and is similar to dates obtained in this project for Covalejos Levels B/2 and C/3, Ekain Level Xa and Cobrante Level 6. A re-analysis of the lithic assemblage recovered from the central part of Level III revealed that it contains diagnostic Aurignacian technological and typological features. The bladelets made from prismatic cores on flakes, with slightly convergent negative scars, are narrow, straight, pointed ones This kind of production is identical to that used in Morín Level 8 [50], Castillo Level 16 [51], Labeko Koba Level VII [52] and Isturitz Levels C4dI C4III [53-54]. Also, this assemblage is characterized by the presence of end-scrapers, some of them on retouched blades, but none typically Aurignacian and a total absence of carinated end-scrapers, thick burin-like bladelet cores and Dufour (subtype Dufour) bladelets. There are also some side-scrapers, denticulates, splintered pieces and becs (Rios, personal obs.). Considering the new date and the characteristics of the lithic assemblage of Level III, there is no argument to maintain an attribution to the Gravettian. On the contrary, there are strong indications for a possible attribution of Level III to the Proto-Aurignacian. Thus, Level III has been included within the Bayesian chronological model for the Aurignacian in the Cantabrian region.

The new assay obtained for Level Vb gave a result of 49,500±3,900 (OxA-X-2640-11), which situates this level at the end of the regional Mousterian, and very close to the underlying, recently re-assessed Mousterian sequence at El Cuco [48]. This older date contradicts some of the cultural elements recovered in the same level, such as the ornaments and the Dufour bladelets. However, this contradiction could be explained if those elements were buried in a hole dug into Level Vb sediments during the occupations of overlying Levels III-IV [49]^50^. If this hypothesis is correct, then the IUP - like ornaments and lithics from Level Vb could be attributed to the Proto-Aurignacian. In fact, these kinds of ornaments, specifically the perforated *Littorina obtusata,* are also present in the Proto-Aurignacian of Isturitz (Level 4d) [55]. Taking this into account, Level Vb and the likely necklace or group of shells that might have been used as a composite ornament, can no longer be attributed necessarily to the Gravettian. Further research is needed to prove the hypothesis of Proto-Aurignacian materials mixed into a Late Middle Paleolithic level by prehistoric pit-digging. The date from Level Vb has not been included in the Bayesian chronological model for the late Mousterian of the Cantabrian region because, despite its age, its cultural attribution is unclear.

*Bizkaia*

**Bolinkoba** is a cave site, located in Abandiño (Bizkaia), excavated by J.M. Barandairan and T. Aranzadi between 1932-33 [56], then by J.M. de Urquijo in the 1940’s, and between 2008-14 by M.J. Iriarte Chiapusso [57]. The original sequence contained levels attributed to the Gravettian, Solutrean, Lower and Upper Magdalenian and Azilian [58]. Recent excavations focused on a small area of the site have revealed a sequence of four archaeological levels (1, 2sup,2inf, 3) [57].

Level 2sup, attributed to the Gravettian with two AMS dates of 29,950±120 (Beta-426854) and 21,020±90 (Beta-302981), yielded few lithic artifacts. This level corresponds “grosso modo” with Level VI/F from the Barandiarán excavations. Level 2inf, attributed to the Evolved Aurignacian, yielded a small and undiagnostic lithic assemblage [59], and three coherent AMS dates: 29,770±140 (Beta-302983), 30,780±180 (Beta-302984), 30,520±190 (Beta-426855) and one clearly anomalous date of 100±30 (Beta-402334). This level, unidentified by J. M. Barandiarán, probably corresponds to the base of Level VI/F from the original excavation or with Level VII/G considered sterile by Barandiarán and Aranzadi. Level 3 contained a Mousterian assemblage, tentatively situated at the end of Middle Paleolithic, although dating attempts produced no results [57,59]. For this project, two samples of bones from Barandiarán’s Level VI/F curated in the Bizkaia Museum of Archaeology (Bilbao) were selected for dating.

One of the samples (OxA-32518) yielded a date of 10,605±50 uncal BP, which clearly contradicts the cultural attribution of Level VI/F, since such an age corresponds to the time range of the regional Azilian. We cannot determine whether the sample was incorporated from Level B (Azilian) into Level VI/F assemblage by human pit-digging, by post-depositional processes, or by problems during excavation or curation. No diagnostic Azilian lithic material has been recovered or identified in Level VI assemblage. The other sample from Level VI/F (OxA-32519) gave a more coherent date of 25,280±210 uncal BP, which would represent an intermediate phase of the Noaillian Gravettian between the older occurrences for the regional Gravettian (Aitzbitarte III Levels Vb sup y Va) and the younger phase found of Morín Level 4 and La Viña Level VII. No modelling was undertaken for this site, nonetheless the date was included within the chronological model for the Gravettian in the Cantabrian region.

**Axlor** in Dima (Bizkaia) is a rock-shelter containing a long Middle Paleolithic sequence. The site was initially excavated between 1967-1974 by J. M. Barandiarán, who identified a series of Mousterian levels (I-VIII) [60]. Subsequent excavations between 1999-2004 were focused on obtaining a detailed stratigraphy and correlating it with Barandiarán’s levels [61,62]. Mousterian Levels B-N were lying beneath a practically sterile layer (A) which capped the underlying stratigraphy. The only available dates come from Level D with a result of 42,010 ± 1,280 BP (Beta-144262) and >43,000 (Beta-224586) [61,63-64]. This level roughly corresponds with Level IV from the Barandiarán’s excavation and has been defined as a Quina Mousterian and in which five Neanderthal human bones had been found [60,65]. Level F, corresponding to Level V of the Barandiarán excavations, is the lowest level of the Quina complex at the site and yielded a date of >47,500 (Beta-225487) and another much younger one of 33,310±360 (Beta-225485). This project attempted dates from Level IV/D of Barandiarán’s excavations. The material is currently curated at the Bizkaia Museum of Archaeology (Bilbao).

Both samples (OxA-32428 and OxA-32429) provided dates that go beyond the radiocarbon limit (>49,300 and >49,900, respectively), and are significantly older than the previous date of 42,010±1,280 BP (Beta-144262) obtained for this level and, coherent with another date of >43,000 (Beta-224586) published later. These new results suggest that the human presence at Level IV/D would not correspond to a late occurrence of regional Mousterian as previously proposed. This would imply that the lower levels in the sequence should be also significantly older than previously proposed and accordingly, therefore a complete reappraisal of the chronology at the site is needed.

*Gipuzkoa*

**Ekain**, located in Deba (Gipuzkoa), has a stratigraphic sequence running from the Châtelperronian through the Upper Magdalenian and Azilian, and it is well-known for its late Upper Paleolithic cave art [66]. The lower part of the sequence contains evidence of human presence during the Initial Upper Paleolithic. Level Xa was interpreted as evidence of a Châtelperronian hunting camp, yielded a small lithic collection, including a single typical Châtelperronian point, and three other backed blades and bladelets. Level IXb was interpreted as an Aurignacian hunting camp with a small lithic assemblage, probably no more than a single occupation episode, with typical carinated “end-scraper” bladelet cores and Aurignacian blades [67]. Level IXb was dated by conventional ^14^C to >30,600 (I-11506) [66]. In this project, both Levels (Xa and IXb) were dated. The material is curated by the provincial government and at the time the sampling was undertaken, it was deposited in its repository in San Sebastián.

The new date for Level Xa is 34,350±550 BP (OxA-34930), which is far too young for the regional Châtelperronian, dated at 37-38k uncal BP in nearby Labeko Koba [8]. The bone sampled was located 25 cm below the Aurignacian of Level IXb. At the same depth where the sampled bone was found, one of the four backed blades from the Châtelperronian level had been recovered [68]. Human presence in Level Xa was occasional, however cave bears were very common at the site (97% of faunal assemblage) and we cannot rule out the possibility of admixture caused either by cave bears or by other post-depositional processes not identified during excavation. Thus, we do not consider this date to be valid and it was not included within the chronological model either for the Châtelperronian or the Aurignacian in the Cantabrian region. The two samples in Level IXb were obtained from the same bone, yielding dates of 31,140±400 (OxA-32423) and 31,110±400 (OxA-32424), corresponding to the Aurignacian. These new dates provided more precision than the ^14^C conventional date and are contemporaneous with the Evolved Aurignacian from Aitzbitarte III Vb center and La Viña Levels XIII and XII. Initial analysis of this level placed it into an indeterminate Aurignacian [66]. A later analysis by one of us (JR), also considering the interpretation limits imposed by the small size of the collection, suggested a possible Early Aurignacian attribution [67] which, now, however, cannot be maintained. Bayesian modelling is shown in S4 Fig. The date range for Level IXb was 36,212-33,720 cal BP and was accumulated during GI7. These dating results were included within the chronological model for the Aurignacian in the Cantabrian region.

The site of **Amalda** in Zestoa (Gipuzkoa) is known for its succession of Mousterian (VII) and Gravettian (VI, V) levels and it contains Late Upper Paleolithic levels too [69]. Mousterian Level VII was excavated over a large surface and the nature of its formation has been debated in recent years because one author [70-71] argued that it was mostly a carnivore den deposit, only occasionally occupied by Neanderthals, while others [72-73] defended the original conclusion that it was a human occupation site with occasional carnivore activity. But it never had been dated. A recent analysis of the lithic assemblage showed that evidence of limited admixture with overlying Level VI is present and that strata in the squares excavated at the cave rear were disturbed [63]. The chronological placement of the Mousterian level was tentatively given at the beginning of the Late Würm glaciation (MIS4) [69] but the techno-typology of the lithic assemblage suggested a Late Middle Paleolithic [64]. Level VI was defined as a Gravettian rich in Noailles burins and was dated by conventional radiocarbon to 27,400±1100 (I-11664) and 27,400±1000 (I-I11665). For Level VI, similar taphonomic issues as in Level VIII suggesting an important role for carnivores in bone accumulation [74]. Level V, also defined as Gravettian, was dated by conventional radiocarbon to 19,000±340 (I-11663) for the base and 17,880 ±390 (I-11732) and the middle part of the level. A recent spatial analysis suggests that Level V did not suffer any severe post-depositional alteration [75]. In this project, one date for Level V, three for Level VI and three more for Level VII were attempted. The material is curated by the provincial government and at the time the sampling was conducted, it was deposited in a repository in San Sebastian.

Three assays were attempted for Amalda Level VII (Mousterian) and the dates obtained confirm this cultural attribution. Two assays on bones, located on the main area of lithic artifact concentration, provided dates of 44,500±2100 (OxA-32500) and 42,600±1600 (OxA-34933). These two dates add new information on the chronology of the late Mousterian in the region as recently published [76]. The third sample, obtained from a square with few lithics, yielded a date of 28,640 (OxA-32425), which is clearly out of the range for the Mousterian, and very close to the new dates obtained for the Gravettian occupations (Level VI) at the site. This could reflect a limited admixture between Levels VI and VII that has been already detected in the lithic assemblage [63]. This admixture of materials was probably due to the difficulties in differentiating the two levels, from a sedimentological point of view, during excavation.

Two of the three samples belonging to Level VI dated in this project were: 28,540±310 (OxA-32426) and 28,710±300 (OxA-34934). Both are clearly consistent with a Gravettian attribution, although these new dates are slightly older than previous conventional ^14^C dates which might be due to removal modern contamination as a result of the ultrafiltration method. This new chronology situates this level in an early phase of the Noaillian facies of the Gravettian, which would be contemporaneous with Aitzbitarte Level IV, La Viña X and Llonín V. The third date obtained from Gravettian Level VI gave an unexpected result of 886±26 (OxA-32501), which corresponds to XI-XIII century. This sample came from the upper part of the level from a square that was close to the cave entrance, where the upper levels were exposed. Then, we cannot dismiss the possibility that materials from historic times were inserted into Last Glacial sediments in this area of the site. Finally, a single sample from Amalda Gravettian Level V was also dated. The result of 14,745±75 (OxA-32427) was completely unexpected as the overlying level (Level IV) is clearly a terminal Solutrean (ca. 18-16.500 uncal BP). This date may reflect from unknown problems during excavation or curation, or an admixture between levels, such as pit-digging.

A Bayesian age model for Amalda is shown in S5 Fig. The date range for Level VII is 53,778-33,308 cal BP and for Level VI is 44,974-26,598 cal BP (95.4% probability). When the valid dates are compared to the NGRIP GICC05 record, we conclude that Level VII was accumulated between stadial GS13 and interstadial GI12 and Level VI between GI6 and GI5.

**Aitzbitarte III** is part of a karstic system with five different caves in Errentería, Gipuzkoa, near San Sebastián. The most recent excavations and analyses of material from Aitzbitarte III were undertaken at the entrance area of the cave between 1994-2002, revealing a cultural sequence of Mousterian, Evolved Aurignacian and Gravettian [77-78]. Level Vb was excavated by artificial spits adapted to the stratigraphy in each square. Level V was originally attributed to the Aurignacian and subdivided into three parts (from bottom to top: Vb base, Vb central and Vb upper) from technological and typological characteristics and the presence of lithic artifact refits. The analysis of Level “Vb base” revealed admixture with underlying Mousterian Level VI, which was excavated over a very limited surface. Level “Vb central” was interpreted as Evolved Aurignacian, with typical tools such as Vachons points, Busqué burins, Dufour bladelets and carinated cores (“burins” and “endscrapers”) [79]. Recent AMS dates [80] situated Level Vb between 28,010±600 (Ua-21160) and 33,605±1165 (Ua-21158), Level “Vb upper” and Levels Va and IV were interpreted as Early Gravettian with Noailles burins. AMS dating for Level Va provided dates between 31,210±860 (Ua-18467) and 26,350±475 (Ua-24967), and for Level IV between 28,320±605 (Ua-18465) and 22,420±365 (Ua-24965). Level III was attributed to a very recent phase of the Gravettian, with Noailles burins and carinated end-scraprers, and dated between 22,580±295 (Ua-24963) and 18,400±215 (Ua-11159).

Here, nine dates were attempted: two each for Levels IV, Va and Vb upper, and three for Level Vb central. The dates for Level Vb central are 31,130±390 (OxA-34932) and 31,600±400 (OxA-32418) which are coherent with previous AMS dates and the cultural attribution. This chronology would represent an evolved phase of the Aurignacian, contemporaneous of Ekain IXb and La Viña Levels XIII and XII. The third date in Level Vb central yielded a result of 34,900±600 (OxA-32417). In the Bayesian model this date is found to have 73% of probability of being an outlier (excluded from the model) appearing older than expected. However, we do not discount the possibility that this date could represent the presence of an Early Aurignacian at the base of the level, although the presence of numerous refits between lithic artifacts across this unit [80] suggests a certain degree of coherence on the formation of the level. Further dating in this level will be necessary to confirm whether the date is an outlier or not.

Level Vb upper was archaeologically separated from Vb central due to certain characteristics of the lithic assemblage that suggested different cultural attributions [79]. The presence of distinctive Noaillian burins indicated an Early Gravettian period in Level Vb upper. However, the dates obtained from this sub-level of 31,950±450 (OxA-32419) and 30,990±390 (OxA-32416) are similar to those obtained in Vb central, indicating either an Evolved Aurignacian that already shows some features transitional to the Gravettian or the first manifestation of an Early Gravettian at the site [44]. If the latter is true, that would indicate a relatively quick cultural change from the Aurignacian to the Gravettian at the site. Indeed, the presence of atypical large Noailles burins in Level Vb central also introduces the possibility of a local origin for the Noaillian [44].

The new dates in Level Va, previously identified as Early Gravettian with Noaillian burins [79], apparently support a quick transition towards the Gravettian, as they are very similar between them: 31,300±400 (OxA-32421) and 31,090±400 (OxA-32420) and with dates obtained in Levels Vb central and Vb upper. Finally, for Level IV, also classified as Early Gravettian with Noailles burins, the new dates of 29,130±310 (OxA-32422) and 29,020±320 (OxA-32499) are consistent with the early Gravettian dates obtained in Amalda VI and La Viña X. In summary, the new dates obtained here for Aitzbitarte III are coherent with previous dates [80], just slighter older due to application of ultrafiltration pre-treatment in this project.

The Bayesian age model for Aitzbitarte III is shown in S6 Fig. The date range for Level Vb central is 36,136-34,968 cal BP; for Level Vb upper it is 35,662-34,808 cal BP; for Level Va it is 35,366-33,748 and for Level IV is 34,824-32,486 (at 95.4% probability). The boundary between the Evolved Aurignacian and the Early Gravettian is 35,810-34,912 cal BP, whereas the boundary for the end of Level IV is 33,834-31,662 cal BP. The span of dates within Level Vb central is 1,168 years, in Level Vb upper 854 years, 1,618 years in Level Va and 2,172 years in Level IV. These dates indicate a rapid accumulation of Levels Vb central and upper. The results of the “order” function indicate that Level Vb central was accumulated 100% before Vb upper, and the latter before Level Va. When compared to the NGRIP GICC05 record, Levels Vb and Va were apparently accumulated during GI7 and Level IV between GI6 and GI5.

*Bayesian approach*

Calibrated and modelled probability distributions of models were done using OxCal [81-82] against IntCal13 [83]. Convergence values are also given. These should be above 95% [84].

The process adopted is the following:

- We have modelled individual sites following the usual procedure when constructing Bayesian models. These models have provided both the PDFs of the boundaries between Phases and the modelled dates incorporating the information derived from the stratigraphy. The command Date included in the code allows obtaining a PDF for the whole duration of each Phase for interpretation purposes only. It is not altering the results in any way, and it is not used later for the general models.
- We have saved the PDFs of each modelled date as Priors. This OxCal operation allows using the modelled date in subsequent modelling. For tracking purposes, we have included the OxA number in the name of the Prior file. This means that Prior La_Vina_XI_OxA_19195 is the modelled PDF of the OxA-19195 radiocarbon date retrieved from Level XI of La Vina. This PDF is a combination of the calibrated date and the stratigraphy at each site, and as such, it is considered a better representation of the date than the calibrated one only.
- We have modelled each cultural period by pooling all dates belonging to that period in a single phase:
  - In the cases where a single site model was not possible, the uncalibrated date was used.
  - However, in the cases where a single site model was possible, the modelled PDF of the date was used (i.e. the prior file saved before)

As we have discussed above, this is including the hypothesis of cultural synchronicity across the region, i.e. cultures would have appeared and disappeared in the region at approximately the same time. This new information serves to constrain the boundaries between cultures further. What OxCal does is:

- - For uncalibrated dates, it calibrates the date and obtain a PDF
  - For Priors, it directly takes the modelled PDF obtained from the single site model and uses it as it were the calibrated PDF. This is actually assuming that the posterior PDF coming from the individual model is used as prior PDF in the general models.
  - It performs a Markov Chain Monte Carlo (MCMC) analysis to do a subsequent Bayesian inference including the new information of regional synchronicity of cultures.
  - It finally obtains new modelled PDFs, both for the only calibrated dates and, the ones previously modelled at individual dates.

This procedure, known as Recursive Bayesian estimation or Sequential Bayesian filtering, is widely used in other disciplines, such as computer sciences, geo-positioning techniques or environmental sciences, to modify the PDFs of states or parameters of models each time, a new observation becomes available. In our case, using all the uncalibrated dates in the general models would mean that the stratigraphy information would be lost, and that is essential information to be considered. Likewise, interpreting only the boundaries coming from individual models would neglect the likely regional cultural synchronicity. However, with the adopted approach, the stratigraphy is first incorporated to obtain a modelled (Posterior) PDF of the dates at each single site, and subsequently, they are used as unmodeled (Prior) PDFs when adding the hypothesis of cultural synchronicity. In this way, more information is applied and the resulting boundaries are more reliable.

*Sensitivity tests*

In order to check the robustness of the modelled results, in particular regarding the Probability Distribution Functions of the boundaries between cultures, which are the relevant ones for the conclusions of this research, the following sensitivity tests were undertaken:

For individual models:

- Using a trapezium prior model to examine the effects of changing the usual uniform boundaries. Trapezium models place initially more probability in the middle than in the extremes.
- Changing the general outlier model to use a wider t-student function with 1 degree of freedom.
- Changing the prior probability of being and outlier from 5% to 10% for all dates.
- Leaving the detected outliers in the model as only if a date is considered an outlier with a probability of 100%, the Bayesian model will completely discard it.

For regional models, the youngest and oldest date in each cultural phase is considered *terminus post quem* or *ante quem*, and thus removed from the analysis.

Variations in individual models (see S4 Table) resulted in marginal changes of the relevant boundaries between cultures, confirming the robustness of the analysis.

In the case of regional models, (see S5 Table), only removing the date from El Castillo Level 16 implies a significant change, pushing forward the start of the Aurignacian around 1,200 years, and reducing drastically the probabilities of overlap with the Châtelperronian.

**SUPPLEMENTARY INFORMATION REFERENCES**

- 1. Fortea J. Abrigo de La Viña. Informe de las campañas 1980-1986. Excavaciones Arqueológicas en Asturias (1983-1986). 1990; 1: 55-68.

1. Fortea J. Abrigo de La Viña. Informe de las campañas 1987-1990. Excavaciones Arqueológicas en Asturias (1987-1990). 1992; 2: 19-28.
2. Fortea J. Abrigo de La Viña. Informe y primera valoración de las campañas 1991 a 1994. Excavaciones Arqueológicas en Asturias (1991-1994). 1995; 3: 19-32.
3. Fortea J. Le Paléolithique supérieur en Espagne, Galice et Asturies, 1991-1995. En M. Otte (dir.), Le paléolithique supérieur Européen: bilan quinquennal 1991-1996. ERAUL 76. Liège: Université de Liége; 1996. pp. 329-339.
4. Fortea J. 1999. Abrigo de La Viña. Informe y primera valoración de las campañas de 1995 a 1998. Excavaciones Arqueológicas en Asturias (1995-1998), 4: 31-41.
5. Santamaría, D., La transición del Paleolítico Medio al Superior en Asturias. El abrigo de La Viña (La Manzaneda, Oviedo) y la Cueva de El Sidrón (Borines, Piloña). PhD thesis. Oviedo: Servicio de Publicaciones de la Universidad de Oviedo. 2012. Available from: <http://hdl.handle.net/10651/19328>.
6. Martínez, L. El Gravetiense en el sector occidental cantábrico y sus conexiones pirenaicas. PhD Thesis. 2015.Oviedo: Servicio de Publicaciones de la Universidad de Oviedo. <http://hdl.handle.net/10651/33635>
7. Wood RE, Arrizabalaga A, Camps M, Fallon S, Iriarte-Chiapusso MJ, Jones R, et al. The chronology of the earliest Upper Palaeolithic in northern Iberia: New insights from l'Arbreda, Labeko Koba and La Viña. J Hum Evol. 2014; 69: 91-109. doi.org/10.1016/j.jhevol.2013.12.017

Zilhão J. Chronostratigraphy of the Middle-to-Upper Paleolithic Transition in the Iberian Peninsula. Pyrenae 2006; 37(1): 7-84.

1. Bradtmöller M. Höhlenlager des Gravettien - Muster jungpaläo¬lithischer Höhlennutzung am Beispiel des Gravettien Nordspaniens. Hamburg: Verlag Dr. Kovač; 2014.
2. Fortea J, de la Rasilla M, Rodríguez V. La cueva de Llonín (Llonín, Peñamellera Alta). Campañas de 1991 a 1994. Excavaciones Arqueológicas en Asturias 1987-1990, Oviedo: Servicio de Publicaciones del Principado de Asturias; 1992; 2: pp. 9-18.
3. Fortea J, de la Rasilla M, Rodríguez V. La cueva de Llonín (Llonín, Peñamellera Alta). Campañas de 1991 a 1994. Excavaciones Arqueológicas en Asturias 1991-1994; 1995; 3: pp. 33-44.
4. Fortea J, de la Rasilla M, Rodríguez V. La cueva de Llonín (Llonín, Peñamellera Alta). Campañas de 1995 a 1998. Excavaciones Arqueológicas en Asturias 1995-1998; 1999; 4: pp. 59-68.
5. Fortea J, de la Rasilla M, Rodríguez V. L’art pariétal et la séquence archéologique paléolithique de la grotte de Llonín (Peñamellera Alta, Asturies, Espagne). Préhistoire, Art et Sociétés. BSPA. 2004; LIX : 7-29.
6. Fortea J, de la Rasilla M, Rodríguez V. La cueva de Llonín (Llonín, Peñamellera Alta). Campañas de 1999 a 2002. Excavaciones Arqueológicas en Asturias 1999-2002; 2007; 5: pp.77-86.
7. Martínez L, de la Rasilla M. El Gravetiense en Asturias: revisión y novedades. En C. de las Heras, J. A. Lasheras, A. Arrizabalaga y M. de la Rasilla (coord.), Pensando el Gravetiense: nuevos datos para la región cantábrica en su contexto peninsular y pirenaico. Madrid: Monografías del Museo Nacional y Centro de Investigación de Altamira, 23. Ministerio de Educación, Cultura y Deporte. 2013; pp. 291-303.
8. Santamaría D, de la Rasilla M. Datando el final del Paleolítico medio en la Península Ibérica. Problemas metodológicos y límites de la interpretación. Trabajos de Prehistoria, 2013; 70 (2): 241-263.
9. de la Rasilla M, Santamaría D. El Paleolítico Medio en Asturias. Mainake 2011-12; XXXIII: 31-62.
10. Altuna J, Mariezkurrena K, de la Peña P, Rios-Garaizar J. Los niveles gravetienses de la cueva de Aitzbitarte III (Gipuzkoa). Industrias y faunas asociadas, in: de las Heras, C., Lasheras, J.A., Arrizabalaga, Á., de la Rasilla, M. editors. Pensando El Gravetiense: Nuevos Datos Para La Región Cantábrica En Su Contexto Peninsular Y Pirenaico. Monografías Del Museo Nacional Y Centro de Investigación de Altamira, 23. Madrid: Ministerio de Educación, Cultura; 2013. pp. 184–204.

Sanguino González J, Montes Barquín R. Nuevos datos para el conocimiento del Paleolítico Medio en el centro de la Región Cantábrica: La Cueva de Covalejos. Santander: Museo de Altamira Monografías. 2005; 20: 10-38.

1. Maroto J, Vaquero M, Arrizabalaga A, Baena J, Baquedano E, Jordá J, Julià R, Montes R, Van Der Plicht J, Rasines P. Wood R. Current issues in late Middle Palaeolithic chronology: New assessments from Northern Iberia. *Quat Internat.* 2012; 247:15-25. doi:10.1016/j.quaint.2011.07.007
2. Wood R, de Quirós FB, Maíllo-Fernández JM, Tejero JM, Neira A, Higham T. El Castillo (Cantabria, northern Iberia) and the Transitional Aurignacian: Using radiocarbon dating to assess site taphonomy. Quat Int. Forthcoming; [doi.org/10.1016/j.quaint.2016.03.005](http://dx.doi.org/10.1016/j.quaint.2016.03.005)
3. Higham T, Jacobi R, Basell L., Ramsey CB, Chiotti L, Nespoulet R. Precision dating of the Palaeolithic: A new radiocarbon chronology for the Abri Pataud (France), a key Aurignacian sequence. J Hum Evol 2011; 61: 549–563. doi:http://dx.doi.org/10.1016/j.jhevol.2011.06.005
4. Talamo S, Soressi M, Roussel M, Richards M, Hublin JJ. A radiocarbon chronology for the complete Middle to Upper Palaeolithic transitional sequence of Les Cottés (France). J ArchaeolSci 2012; 39: 175–183. doi:http://dx.doi.org/10.1016/j.jas.2011.09.019
5. Muñoz Fernández E. Excavaciones arqueológicas en la Cueva del Ruso I. Avance preliminar. Arquenas. 1991: 61-157.
6. Muñoz Fernández E, Serna Gancedo A. Los niveles solutrenses de la Cueva del Ruso I (Igollo de Camargo, Cantabria). Sautuola: Revista del Instituto de Prehistoria y Arqueología. 1999; 6: 161-176.
7. Muñoz Fernández E, San Miguel Llamosas C. La arqueología de Camargo. In: Peña Fernández, A. editor. Camargo. Historia y Patrimonio. Actas de los Encuentros de Historia de Camargo (Herrera, 2-7 Noviembre 1998; Muriedas, 8-12 Noviembre 1999). Santander: Ayuntamiento de Camargo; 2001. pp. 36-56.
8. Álvarez Fernandez E. Atlantic versus Mediterranean: Personal ornaments made from mollusc shells in Europe during the Upper Palaeolithic and Mesolithic (Text), in BoneCommons, Item #463, 2006. Available from: http://alexandriaarchive.org/bonecommons/items/show/463 (accessed January 13, 2017).
9. Castanedo Tapia I. Aproximación a las cadenas operativas líticas del Paleolítico en Cantabria: las cuevas de La Flecha y de El Ruso I. Master Thesis. Universidad de Cantabria. 1997. Available from: <http://catalogo.unican.es/cgi-bin/abnetopac/?TITN=196907>
10. González Echegaray J, Freeman LG. Cueva Morín: Excavaciones 1966-1968. Santander: Patronato de Cuevas Prehistóricas; 1971.
11. Sanguino J, Montes R, Martín Blanco P, El marco cronoestratigráfico y paleoclimático del Pleistoceno superior inicial de la región cantábrica, ¿un gigante con pies de barro? In: Santonja M, Pérez-González A, Machado MJ, editors. Geoarqueología y Patrimonio en la Península Ibérica y el entorno mediterráneo. Soria: ADEMA; 2005.pp. 127-138.
12. Stuckenrath R. Dataciones de Carbono 14. In: González Echegaray J, Freeman LG. editors. Vida y muerte en Cueva Morín. Santander: Institución Cultural de Cantabria; 1978. pp. 215.
13. Maíllo-Fernández JM, Valladas H, Cabrera V, Bernaldo de Quirós F. Nuevas dataciones para el Paleolítico superior de Cueva Morín (Villanueva de Villaescusa, Cantabria). Espacio, Tiempo Y Forma. 2001; 14: 145-150.
14. Maíllo-Fernández JM, Arteaga C, Iriarte-Chiapusso MJ, Fernández A, Wood R, Bernaldo de Quirós F. Cueva Morín (Villanueva de Villaescusa, Cantabria), in: Sala R, Carbonell E, Bermúdez de Castro JM, Arsuaga JL, editors. Los Cazadores Recolectores Del Pleistoceno Y Del Holoceno En Iberia Y El Estrecho de Gibraltar. Burgos: Universidad de Burgos; 2014. pp. 72–78.
15. Bradtmöller M. The Gravettian occupation of Level 4 Cueva Morín and its regional context. Munibe. 2015; 66: 23-52.
16. Rasines del Río P. Geografía, estratigrafía y cronología de la cueva de Cobrante. Sautuola 2009; 15: 43-47.
17. Muñoz Fernández E, Santamaría Santamaría S. Análisis de la industria lítica de la cueva de Cobrante. Sautuola 2009; 15: 145-189.
18. Corchón Mª S. Solutrense y magdaleniense del oeste de la cornisa cantábrica: dataciones C14 (Calibradas) y marco cronológico. Zephyrus 1999; 52: 3-32.
19. Straus L, Clark GA. editors. La Riera Cave: Stone Age hunter-gatherer adaptations in northern Spain. Tempe: Arizona State University; 1986
20. Valladas H, Cachier H, Arnold M, Clottes J, Bernaldo de Quirós F, Cabrera Valdés V, Uzquiano Ollero P. Direct Radiocarbon dates for the prehistoric paintings at the Altamira, El Castillo and Niaux caves. Nature. 1992; 357: 68-70. doi:10.1038/357068a0
21. Straus LG, Gonzalez Morales MR, Marín-Arroyo AB, Iriarte Chiapusso MJ. The human occupations of El Mirón Cave (Ramales de la Victoria, Cantabria, Spain) during the Last Glacial Maximum/Solutrean period. Espacio Tiempo y Forma. Serie I, Prehistoria y Arqueología, 2013; 1(5): 1-26.
22. González Echegaray JG. Cueva del Otero. Excavaciones Arqueológicas en España, 53. Madrid: Ministerio de Educación Nacional Dirección General de Bellas Artes Servicio Nacional de Excavaciones; 1966.
23. Freeman LG. Mousterian Developments in Cantabrian Spain. Ph.D. thesis. Dept. of Anthropology, University of Chicago, Chicago; 1964.
24. Rios-Garaizar J, de la Peña P, Maillo-Fernández JM, 2013. El final del Auriñaciense y el comienzo del Gravetiense en la región cantábrica: una visión tecno-tipológica, in: de las Heras C, Lasheras JA, Arrizabalaga Á, de la Rasilla M. editors. Pensando El Gravetiense: Nuevos Datos Para La Región Cantábrica En Su Contexto Peninsular Y Pirenaico. Monografías Del Museo Nacional Y Centro de Investigación de Altamira, N.o 23. Madrid; Ministerio de Educación, Cultura, 2013; pp. 369–382.
25. Almeida F. Looking for names and missing the point. The case of the Portuguese “Aurignacian V” in the framework of definitions of the Aurignacian. In: Bar-Yosef O, Zilhao J. editors. Towards a definition of the Aurignacian. Trabalhos d Arqueologia 2006; 45: 71-92.
26. Bernaldo de Quirós F. Los inicios del Paleolítico Superior Cantábrico. Santander: Monografías del Museo y Centro de estudios de Altamira 8; 1982.
27. Muñoz Fernández E, Rasines del Río P, Santamaría Santamaría S, Morlote Expósito JM, Estudio arqueológico del abrigo del Cuco. In: Muñoz Fernández E, Montes Barquín R, editors. Intervenciones arqueológicas en Castro Urdiales, tomo III. Arqueología y arte rupestre paleolítico en las cavidades de El Cuco o Sobera y La Lastrilla. Castro Urdiales: Excmo. Ayuntamiento de Castro Urdiales. Concejalía de Medio Ambiente y Patrimonio Arqueológico; 2007. pp. 15-160.
28. Gutierrez-Zugasti I, Rios-Garaizar J. Marín-Arroyo AB, Rasines P, Maroto J, Jones J, Bailey G, Richards M. Forthcoming. A chrono-cultural reassessment of the levels VI-XIV from El Cuco rock-shelter: a new sequence for the Late Middle Paleolithic in the Cantabrian Region (northern Iberia). Quat Int <https://doi.org/10.1016/j.quaint.2017.06.059>
29. Gutiérrez-Zugasti I, Cuenca-Solana D, Rasines del Río P, Muñoz E, Santamaría S, Morlote J. The role of shellfish in hunter-gatherer societies during the early Upper Palaeolithic: a view from El Cuco rockshelter, Northern Spain. J Anthropol Archaeol 2013; 32 (2): 242–256. doi.org/10.1016/j.jaa.2013.03.001
30. Maíllo JM. Tecnología lítica en el Auriñaciense arcaico de Cueva Morín (Villanueva de Villaescusa, Cantabria). In: Bon F, Maíllo-Fernández JM, Ortega D, editors. Autour des concepts de Protoaurignacien, d’Aurignacien archaïque, initial et ancien. Espacio, Tiempo y Forma 2002; 15: 87–116.
31. Maíllo JM, Bernaldo de Quirós F. Archaic Aurignacian in El Castillo cave (Spain): technology and typology composition. Anthropologie 2010; 114(1): 1-25.
32. Arrizabalaga A. Los tecnocomplejos líticos del yacimiento arqueológico de Labeko Koba (Arrasate, País Vasco). In: Arrizabalaga A, Altuna J. editors. Labeko Koba (País Vasco): hienas y humanos en los albores del Paleolítico superior. Munibe 2000; 52: 193–343.
33. Normand Ch. L’Aurignacien de la Salle de Saint-Martin (Grotte d’Isturitz, commune de Saint-Martin d’Arberoue, Pyrénées-Atlantiques): données préliminaires sur l’industrie lithique recueillie lors des campagnes 2000–2002. In: Bon F, Maíllo-Fernández JM, Ortega D, editors. Autour des concepts de Protoaurignacien, d’Aurignacien archaïque, initial et ancien. Espacio, Tiempo y Forma 2002; 15: 145–174.
34. Normand C, O’Farrell M, Rios-Garaizar J. Quelles(s) utilisations(s) pour les productions lamellaires de l’Aurignacien archaïque ? Quelques données et réflexions à partir des exemplaires se la grotte d’Isturitz (Pyrénées Atlantiques; France). Paléthnologie 2008; 1: 7–46.
35. White R. Systems of personal ornamentation in the Early Upper Palaeolithic: methodological challenges and new observations. In: Mellars P, Boyle K, Bar-Yosef O, Stringer C. editors. Rethinking the Human Revolution: New Behavioural and Biological Perspectives on the Origin and Dispersal of Modern Humans. Cambridge: McDonald Institute for Archaeological Research; 2007. pp. 287–302.
36. Barandiarán JM. Bolinkoba y otros yacimientos paleolíticos en la sierra de Amboto (Vizcaya). Cuadernos de historia primitiva 5, 2. Madrid: Seminario de Historia Primitiva del Hombre; 1950.
37. Iriarte-Chiapusso MJ, Arrizabalaga A. El yacimiento arqueológico de Bolinkoba (Abadiño, Bizkaia). Crónica de las investigaciones en la cavidad. Secuencia estratigráfica y cronología numérica, in: Iriarte-Chiapusso MJ, Arrizabalaga A, editors. Bolinkoba (Abadiño) Y Su Yacimiento Arqueológico: Arqueología de La Arqueología Para La Puesta En Valor de Su Depósito, a La Luz de Las Excavaciones Antiguas Y Recientes. Kobie Serie BAI 6. Diputación Foral de Bizkaia, Bilbao, 2015; pp. 5–88.
38. Barandiarán Maestu I. Paleomesolítico del Pirineo Occidental. Bases para la sistematización tipológica del instrumental óseo paleolítico. Zaragoza: Monografías Arqueológicas. Universidad de Zaragoza; 1967.
39. Arrizabalaga A, Iriarte-Chiapusso MJ. Los tecnocomplejos líticos en el yacimiento de Bolinkoba (Abadiño, Bizkaia). La contribución de las excavaciones recientes., in: Iriarte-Chiapusso MJ, Arrizabalaga A, editors. Bolinkoba (Abadiño) Y Su Yacimiento Arqueológico: Arqueología de La Arqueología Para La Puesta En Valor de Su Depósito, a La Luz de Las Excavaciones Antiguas Y Recientes. Kobie Serie BAI 6. Bilbao: Diputación Foral de Bizkaia, 2015; pp. 127–148.
40. Barandiarán JM. Excavaciones en Axlor. 1967- 1974. En: Barandiarán, J. M.: Obras Completas. Tomo XVII; 1980. pp. 127-384.
41. González-Urquijo JE, Ibáñez-Estévez JJ. Abrigo de Axlor (Dima). Arkeoikuska: Investigación arqueológica 2001; 2002: 90–93.
42. González Urquijo JE, Ibáñez Estévez JJ, Rios-Garaizar J, Bourguignon L, Castaños Ugarte P, Tarriño Vinagre A. Excavaciones recientes en Axlor. Movilidad y planificación de actividades en grupos de neandertales. In: Montes Barquín R, Lasheras Corruchaga JA, editors. Actas de La Reunión Científica: Neandertales Cantábricos. Estado de La Cuestión. Monografías Del Museo Nacional Y Centro de Investigación de Altamira No 20. Madrid: Ministerio de Cultura; 2005.pp. 527–539.
43. Rios-Garaizar J. Industria lítica y sociedad en la Transición del Paleolítico Medio al Superior en torno al Golfo de Bizkaia. Santander: PUbliCan - Ediciones de la Universidad de Cantabria; 2012.
44. Rios-Garaizar J. A new chronological and technological synthesis for Late Middle Paleolithic of the Eastern Cantabrian Region. Quat Inter Forthcoming. http://dx.doi.org/10.1016/j.quaint.2016.02.020. online 17 March 2017
45. Basabe JM. Dientes humanos del Musteriense de Axlor (Dima, Vizcaya). Trabajos de Antropología. 1973; 16: 187–207
46. Altuna, J., Merino, J. El Yacimiento Prehistórico de la Cueva de Ekain. San Sebastian: Sociedad de Estudios Vascos; 1984.
47. Rios-Garaizar J. El nivel IXb de Ekain (Deba, Gipuzkoa): Una ocupación efímera del Auriñaciense Antiguo. Munibe. 2011; 62: 87–100.
48. Rios-Garaizar J, Arrizabalaga Á, Villaluenga A. Haltes de chasse du Châtelperronien de la Péninsule Ibérique. Labeko Koba et Ekain (Pays Basque Péninsulaire). L’Anthropologie 2012; 116: 532–549. doi.org/10.1016/j.anthro.2012.10.001
49. Altuna J. Situación y descripción de la cueva de Amalda. Historia de las excavaciones. Descripción del relleno. Estructuras en el yacimiento. Dataciones de radiocarbono. Otros yacimientos del valle. In: Altuna J, Baldeón A, Mariezkurrena K, editors. La Cueva de Amalda (Zestoa, País Vasco). Ocupaciones Paleolíticas Y Postpaleolíticas. Sociedad de Estudios Vascos, Donostia-San Sebastián; 1990. pp. 9–31.
50. Yravedra J. Acumulaciones biológicas en yacimientos arqueológicos: Amalda VII y Esquilleu III-IV. Trabajos de Prehistoria. 2006; 63: 55–78.
51. Yravedra J. Nuevas contribuciones en el comportamiento cinegético de la Cueva de Amalda. Munibe. 2007; 58: 43–88.
52. Altuna J, Mariezkurrena K. Tafocenosis en yacimientos del País Vasco con predominio de grandes carnívoros. Consideraciones sobre el yacimiento de Amalda. In: Baquedano E, Rosell J, editors. Zona Arqueológica, No 13, Reunión de Científicos Sobre Cubiles de Hiena (Y Otros Grandes Carnívoros) En Los Yacimientos Arqueológicos de La Península Ibérica (1. 2009. Alcalá de Henares). Alcalá de Henares: Vicepresidencia, Consejería de Cultura y Deporte y Portavocía del Gobierno. Museo Arqueológico Regional; 2010. pp. 214–228.
53. Rios-Garaizar J. Organización económica de las sociedades Neandertales: el caso del nivel VII de Amalda (Zestoa, Gipuzkoa). Zephyrus 2010; LXV: 15–37.
54. Yravedra J. Interacción de humanos y carnívoros en el Pleistoceno Superior de la Península Ibérica. Novedosas interpretaciones en la cueva de Amalda. Espacio, Tiempo y Forma. Serie I, Prehistoria y arqueología, 2004; 16-17: 79–98.
55. Bradtmöller M. Knapping spots and palimpsests - New aspects from Amalda, Level V. In: de las Heras C, Lasheras JA, Arrizabalaga A, de la Rasilla M, editors. Pensando El Gravetiense: Nuevos Datos Para La Región Cantábrica En Su Contexto. Ministerio de Educación, Cultura, Madrid; 2013. pp. 401–415.
56. Higham T, Douka K, Wood R, Ramsey CB, Brock F, Basell L, et al. The timing and spatiotemporal patterning of Neanderthal disappearance. *Nature* 2014; 512: 306-309. doi: 10.1038/nature13621
57. Altuna J, Mariezkurrena K, Rios-Garaizar J. Ocupaciones humanas en la cueva de Aitzbitarte III (Renteria, País Vasco) sector Entrada: 33.000-18.000 BP. Vitoria-Gasteiz; Eusko Jaurlaritzaren Argitalpen Zerbitzu Nagusia; 2011.
58. Altuna J, Mariezkurrena K, de la Peña P, Rios-Garaizar J. Los niveles gravetienses de la cueva de Aitzbitarte III (Gipuzkoa). Industrias y faunas asociadas, in: de las Heras, C., Lasheras, J.A., Arrizabalaga, Á., de la Rasilla, M. editors. Pensando El Gravetiense: Nuevos Datos Para La Región Cantábrica En Su Contexto Peninsular Y Pirenaico. Monografías Del Museo Nacional Y Centro de Investigación de Altamira, 23. Madrid: Ministerio de Educación, Cultura; 2013. pp. 184–204.
59. Rios-Garaizar J, de la Peña P, San Emeterio, A. Estudio de las industrias líticas y óseas de la cueva de Aitzbitarte III (Zona de la entrada). In: Altuna, J., Mariezkurrena, K., Rios-Garaizar, J. editors. Ocupaciones Humanas En La Cueva de Aitzbitarte III (Renteria, País Vasco) Sector Entrada: 33.000-18.000 BP. Vitoria-Gasteiz: Eusko Jaurlaritzaren Argitalpen Zerbitzu Nagusia, 2011. pp. 81–351.
60. Altuna J. Las cuevas de Aitzbitarte (Landarbaso, Rentería). Situación e historia de las investigaciones, in: Altuna, J., Mariezkurrena, K., Rios-Garaizar, J. (Eds.), Ocupaciones Humanas En La Cueva de Aitzbitarte III (Renteria, País Vasco) Sector Entrada: 33.000-18.000 BP. Eusko Jaurlaritzaren Argitalpen Zerbitzu Nagusia, Vitoria-Gasteiz; 2011. pp. 11–21.

Bronk Ramsey C. Bayesian analysis of radiocarbon dates. Radiocarbon. 2009; 51(1): 337-360. doi.org/10.1017/S0033822200033865

Bronk Ramsey C. Dealing with outliers and offsets in radiocarbon dating. Radiocarbon. 2009; 51 (3): 1023-1045. Doi.org/10.1017/S0033822200034093

Reimer PJ, Bard E, Bayliss A, Beck JW, Blackwell PG, Bronk Ramsey C. et al. IntCal13 and Marine13 radiocarbon age calibration curves 0-50,000 years cal BP. Radiocarbon. 2013; 55 (4): 1869-1887. Doi: 10.2458/azu_js_rc.55.16947

1. Bronk Ramsey C. Radiocarbon calibration and analysis of stratigraphy: The OxCal program. Radiocarbon 1995; 37(2): 425-430.
2. [van Klinken GJ.](http://www.sciencedirect.com/science/article/pii/S0305440398903855?via%3Dihub) Bone Collagen Quality Indicators for Palaeodietary and Radiocarbon Measurements. [J Archaeol Sci](http://www.sciencedirect.com/science/journal/03054403) 1999; 26(6), 687-695. doi.org/10.1006/jasc.1998.0385
