# Supplementary material for: Chronological reassessment of the Middle to Upper Paleolithic transition and Early Upper Paleolithic cultures in Cantabrian Spain
Source: PLoS One. 2018 Apr 18;13(4):e0194708. doi: 10.1371/journal.pone.0194708 (PMC5905894; doi:10.1371/journal.pone.0194708)
Supplement: S2 Table — Cells containing probabilities of >95% are coloured in green, 68–94% in orange and <68 in grey. (DOCX) [file pone.0194708.s003.docx]

S2 Table. Results of the Order function comparing the PDF’s of the boundaries dating the start and the end of the archaeological industries from the Cantabrian Region. Cells containing probabilities of >95% are coloured in green, 68-94% orange and <68 grey.

| **Probability *t*_1_ < *t*_2_** | ***t*_2_** | | | | | |
| --- | --- | --- | --- | --- | --- | --- |
| ***t*_1_** | End Mousterian | Start Châtelperronian | End Châtelperronian | Start Aurignacian | End Aurignacian | Start Gravettian |
| End Mousterian | 0 | 0.9993 | 0.9994 | 0.999 | - | - |
| Start Châtelperronian | 0.000667 | 0 | 0.8278 | 0.6373 | - | - |
| End Châtelperronian | 0.000629 | 0.17225 | 0 | 0.4579 | - | - |
| Start Aurignacian | 0.001006 | 0.3627 | 0.5421 | 0 | 1 | - |
| End Aurignacian | - | - | - | - | 0 | 0.001046 |
| Start Gravettian | - | - | - | - | 0.999 | 0 |
