# Supplementary material for: Chronological reassessment of the Middle to Upper Paleolithic transition and Early Upper Paleolithic cultures in Cantabrian Spain
Source: PLoS One. 2018 Apr 18;13(4):e0194708. doi: 10.1371/journal.pone.0194708 (PMC5905894; doi:10.1371/journal.pone.0194708)
Supplement: S3 Table — Only bone samples with ultrafiltration methods are included. ABA: charcoal fragment treated with a series of acid and base washes; ABOx-SC: charcoal treated with acid and base washes, followed by an oxidation stage and pre-combustion; UF AMS: collagen extracted using the ultrafiltration protocol. (DOCX) [file pone.0194708.s004.docx]

S3 Table. Radiocarbon accelerator dates from the Cantabrian Region mentioned in this work. Only bone samples with ultrafiltration methods are included. ABA: charcoal fragment treated with a series of acid and base washes; ABOx-SC: charcoal treated with acid and base washes, followed by an oxidation stage and pre-combustion; UF AMS: collagen extracted using the ultrafiltration protocol.

| **Site** | **Level** | **Lab Code** | **Method** | **Date(s) uncal 14C** | **+** | **Associated Archaeology** | **References** |
| --- | --- | --- | --- | --- | --- | --- | --- |
| La Viña | XIII | Oxa-21705 | UF, AMS | 31160 | 380 | Aurignacian | Wood et al 2014 |
| La Viña | XIII | Oxa-21845 | UF, AMS | 30650 | 360 | Aurignacian | Wood et al 2014 |
| La Viña | XII | Oxa-21678 | UF, AMS | 31600 | 400 | Evolved Aurignacian | Wood et al 2014 |
| La Viña | XII | Oxa-21689 | UF, AMS | 31500 | 400 | Evolved Aurignacian | Wood et al 2014 |
| La Viña | XI | Oxa-X-2290-19 | UF, AMS | 27900 | 280 | Evolved Aurignacian | Wood et al 2014 |
| La Viña | XI | Oxa-21686 | UF, AMS | 20820 | 130 | Evolved Aurignacian | Wood et al 2014 |
| La Viña | XI | Oxa-21687 | UF, AMS | 30600 | 370 | Evolved Aurignacian | Wood et al 2014 |
| La Viña | XI | Oxa-19195 | UF, AMS | 30130 | 170 | Evolved Aurignacian | Wood et al 2014 |
| La Viña | IX | Oxa-21688 | UF, AMS | 24640 | 190 | Gravettian | Wood et al 2014 |
| La Güelga | 9 | OxA-19244 | UF, AMS | 43700 | 800 | Mousterian | Wood et al 2016 |
| La Güelga | 9 | OxA-19245 | UF, AMS | 44300 | 1200 | Mousterian | Wood et al 2016 |
| Esquilleu | VI | OxA-19965 | UF, AMS | 43700 | 1400 | Mousterian | Wood et al 2016 |
| Esquilleu | VI | OxA-19966 | UF, AMS | 44100 | 1300 | Mousterian | Wood et al 2016 |
| El Castillo | 16 | OxA-22200 | UF, AMS | 38600 | 1000 | Proto-Aurignacian | Wood et al 2016 |
| El Castillo | 16/17 | OxA-22201 | UF, AMS | 39100 | 1000 | sterile | Wood et al 2016 |
| El Castillo | 18B | OxA-21972 | UF, AMS | 45800 | 2300 | Transitional Aurignacian | Wood et al 2016 |
| El Castillo | 18B | OxA-21973 | UF, AMS | 46000 | 2400 | Transitional Aurignacian | Wood et al 2016 |
| El Castillo | 18C | OxA-22403 | UF, AMS | 42700 | 1600 | Transitional Aurignacian | Wood et al 2016 |
| El Castillo | 18C | OxA-22202 | UF, AMS | 43100 | 1700 | Transitional Aurignacian | Wood et al 2016 |
| El Castillo | 18C | OxA-22203 | UF, AMS | 42000 | 1500 | Transitional Aurignacian | Wood et al 2016 |
| El Castillo | 19 | OxA-21974 | UF, AMS | 44900 | 2100 | sterile | Wood et al 2016 |
| El Castillo | Moust.alpha | OxA-10233 | UF, AMS | 42100 | 1500 | Mousterian | Wood et al 2016 |
| El Castillo | Moust.alpha | OxA-10328 | UF, AMS | 45700 | 1700 | Mousterian | Wood et al 2016 |
| El Castillo | Moust.alpha | OxA-10327 | UF, AMS | >45700 | - | Mousterian | Wood et al 2016 |
| El Castillo | Moust.alpha | OxA-10329 | UF, AMS | >43800 | - | Mousterian | Wood et al 2016 |
| El Castillo | Moust.alpha | OxA-10187 | UF, AMS | 42900 | 1400 | Mousterian | Wood et al 2016 |
| El Castillo | Moust.alpha | OxA-10188 | UF, AMS | >47300 | - | Mousterian | Wood et al 2016 |
| El Castillo | 20C | OxA-22204 | UF, AMS | 48700 | 3400 | Mousterian | Wood et al 2016 |
| El Castillo | 20C | OxA-22205 | UF, AMS | 49400 | 3700 | Mousterian | Wood et al 2016 |
| El Castillo | Aurig Delta | OxA-21713 | UF, AMS | 35000 | 600 | Aurignacian | Wood et al 2016 |
| El Castillo | Aurig Delta | OxA-22636 | UF, AMS | 43000 | 1700 | Transitional Aurignacian | Wood et al 2016 |
| El Castillo | Aurig Delta | OxA-22018 | UF, AMS | 42100 | 1500 | Transitional Aurignacian | Wood et al 2016 |
| El Castillo | Aurig Delta | OxA-22637 | UF, AMS | 39900 | 1100 | Transitional Aurignacian | Wood et al 2016 |
| El Mirón | 130 | OxA-33515 | UF, AMS | >45000 | - | Mousterian | Straus & Gonzalez Morales 2016 |
| El Mirón | 130 | OxA-33516 | UF, AMS | 48200 | 3300 | Mousterian | Straus & Gonzalez Morales 2016 |
| Morín | 4 | Poz-66758 | UF, AMS | 23640 | 190 | Gravettian | Bratmöller 2015 |
| Morín | 4 | Poz-66759 | UF, AMS | 23790 | 190 | Gravettian | Bratmöller 2015 |
| Morín | 6 II-1 | Oxa-21877 | UF, AMS | 10380 | 45 | Early Aurignacian | Wood 2011 p.238 |
| Morín | 6 II-1 | Oxa-21878 | UF, AMS | 12435 | 60 | Early Aurignacian | Wood 2011 p.238 |
| Morín | 8 | OxA-19084 | UF, ABA | 40060 | 350 | Archaic-Aurignacian | Maroto et al. 2012 |
| Morín | 9 | GrA-33891 | UF, ABA | 33430 | 250-230 | Archaic-Aurignacian | Maroto et al. 2012 |
| Morín | 10 | GrA-33823 | UF, A only | 29380 | 260-240 | Châtelperronian | Maroto et al. 2012 |
| Morín | 11 | OxA-19083 | UF, ABA | 41800 | 450 | Mousterian | Maroto et al. 2012 |
| Morín | 11 | OxA-19459 | ABOx-SC | 43600 | 600 | Mousterian | Maroto et al. 2012 |
| Arrillor | Lmc | OxA-21986 | UF, AMS | 44900 | 2100 | Mousterian | Wood et al 2016 |
| Arrillor | Lamc | OxA-22654 | UF, AMS | >46800 | - | Mousterian | Wood et al 2016 |
| Arrillor | Lamc | OxA-22655 | UF, AMS | 45600 | 2300 | Mousterian | Wood et al 2016 |
| Labeko Koba | IX | OxA-10104 | UF, AMS | 42200 | 2400 | Sterile | Stuart et al 2004 |
| Labeko Koba | IX | OxA-10102 | UF, AMS | 41500 | 2000 | Sterile | Stuart et al 2004 |
| Labeko Koba | IXinf | OxA-22564 | UF, AMS | 37900 | 900 | Pre-Châtelperronian | Wood et al 2014 |
| Labeko Koba | IXinf | OxA-22560 | UF, AMS | 37400 | 800 | Châtelperronian | Wood et al 2014 |
| Labeko Koba | IXinf | OxA-22561 | UF, AMS | 38000 | 900 | Châtelperronian | Wood et al 2014 |
| Labeko Koba | IXinf | OxA-22562 | UF, AMS | 38100 | 900 | Châtelperronian | Wood et al 2014 |
| Labeko Koba | IXinf | Oxa-22563 | UF, AMS | 37800 | 900 | Châtelperronian | Wood et al 2014 |
| Labeko Koba | IX upper | OxA-21777 | UF, AMS | 37700 | 900 | Sterile | Wood et al 2014 |
| Labeko Koba | IX upper | OxA-21792 | UF, AMS | 36550 | 750 | Sterile | Wood et al 2014 |
| Labeko Koba | IX upper | OxA-23199 | UF, AMS | 38400 | 900 | Sterile | Wood et al 2014 |
| Labeko Koba | IX upper | OxA-22559 | UF, AMS | 36000 | 700 | Sterile | Wood et al 2014 |
| Labeko Koba | IX upper | OxA-22653 | UF, AMS | 36850 | 800 | Sterile | Wood et al 2014 |
| Labeko Koba | VII | OxA-21766 | UF, AMS | 36850 | 800 | Proto-Aurignacian | Wood et al 2014 |
| Labeko Koba | VII | OxA-X-2314-43 | UF, AMS | 36500 | 750 | Proto-Aurignacian | Wood et al 2014 |
| Labeko Koba | VII | OxA-21793 | UF, AMS | 35400 | 650 | Proto-Aurignacian | Wood et al 2014 |
| Labeko Koba | VII | OxA-21840 | UF, AMS | 35250 | 650 | Proto-Aurignacian | Wood et al 2014 |
| Labeko Koba | VI | OxA-21794 | UF, AMS | 32200 | 450 | Early Aurignacian | Wood et al 2014 |
| Labeko Koba | VI | OxA-21841 | UF, AMS | 32150 | 450 | Early Aurignacian | Wood et al 2014 |
| Labeko Koba | VI | OxA-21778 | UF, AMS | 35100 | 600 | Early Aurignacian | Wood et al 2014 |
| Labeko Koba | V | OxA-21767 | UF, AMS | 34750 | 600 | Early Aurignacian | Wood et al 2014 |
| Labeko Koba | V | OxA-21779 | UF, AMS | 34650 | 600 | Early Aurignacian | Wood et al 2014 |
| Labeko Koba | IV | OxA-21780 | UF, AMS | 33550 | 550 | Early Aurignacian | Wood et al 2014 |
| Labeko Koba | IV | OxA-21768 | UF, AMS | 33600 | 500 | Early Aurignacian | Wood et al 2014 |
| Amalda | ? | OxA-10103 | UF, AMS | 21000 | 40 | ? | Stuart et al 2004 |
| Lezetxiki | IIIa | OxA-21715 | UF, AMS | >46500 | - | Transitional Aurignacian? | Maroto et al 2012 |
| Lezetxiki | IIIa | OxA-21837 | UF, AMS | 34550 | 600 (Wood), 190 (Maroto) | Transitional Aurignacian? | Maroto et al 2012 |
| Lezetxiki | IIIa | OxA-22627 | UF, AMS | >46700 | - | Transitional Aurignacian? | Maroto et al 2012 |
| Lezetxiki | III | OxA-22021 | UF, AMS | 29250 | 320 | Transitional Aurignacian? | Maroto et al 2012 |
| Lezetxiki | III | OxA-21838 | UF, AMS | 30830 | 380 | Transitional Aurignacian? | Maroto et al 2012 |
