# Supplementary material for: Chronological reassessment of the Middle to Upper Paleolithic transition and Early Upper Paleolithic cultures in Cantabrian Spain
Source: PLoS One. 2018 Apr 18;13(4):e0194708. doi: 10.1371/journal.pone.0194708 (PMC5905894; doi:10.1371/journal.pone.0194708)
Supplement: S4 Table — (DOCX) [file pone.0194708.s005.docx]

S4 Table. Results of sensitivity test conducted on individual models

| **Site** | **Test** | **Boundary VII(Mousterian)/VI (Gravettian)** | | | |
| --- | --- | --- | --- | --- | --- |
|  |  | **68.2%** | | **95.4%** | |
| **Amalda** | Normal | 46390 | 32328 | 46894 | 32256 |
|  | Trapezium model | 46502 | 32214 | 46984 | 32230 |
|  | Outlier model 1 degree of freedom | 46404 | 32338 | 46838 | 32256 |
|  | 10% Prior probability | 46514 | 32340 | 46884 | 32220 |
| **El Cuco** | **Test** | **Boundary Level Vb (Mousterian)/Hiatus** | | | |
|  |  | **68.2%** | | **95.4%** | |
|  | Normal | 49368 | 45064 | 49890 | 41664 |
|  | Trapezium model | 49392 | 45034 | 49880 | 41624 |
|  | Outlier model 1 degree of freedom | 49374 | 45006 | 49888 | 41600 |
|  | 10% Prior probability | 49310 | 44944 | 49880 | 41584 |
|  | **Test** | **Boundary Hiatus/Level III (Aurignacian)** | | | |
|  |  | 68.2% | | 95.4% | |
|  | Normal | 43166 | 39258 | 46848 | 38710 |
|  | Trapezium model | 43190 | 39248 | 46904 | 38722 |
|  | Outlier model 1 degree of freedom | 43176 | 39262 | 46868 | 38722 |
|  | 10% Prior probability | 43160 | 39274 | 46738 | 38722 |
| **Aizvbitarte III** | **Test** | **Boundary Vb central(Aurignacian)/Vb sup (early Gravettian)** | | | |
|  |  | **68.2%** | | **95.4%** | |
|  | Normal | 35552 | 35098 | 35810 | 34912 |
|  | Trapezium model | 35554 | 35104 | 35816 | 34916 |
|  | Outlier model 1 degree of freedom | 35550 | 35096 | 35810 | 34910 |
|  | 10% Prior probability | 35548 | 35090 | 35804 | 34906 |
|  | With outlier Level Vb central  (OxA-32417) | 35570 | 35110 | 35846 | 34916 |
| **La Viña** | **Test** | **Boundary Level XI (Aurignacian)/X (Gravettian)** | | | |
|  |  | **68.2%** | | **95.4%** | |
|  | Normal | 34176 | 33282 | 34420 | 32790 |
|  | Trapezium model | 34176 | 33282 | 34426 | 32790 |
|  | Outlier model 1 degree of freedom | 34176 | 33280 | 34422 | 32790 |
|  | 10% Prior probability | 34176 | 33280 | 34422 | 32784 |
|  | With outliers of Level XI (OxA-21686 and OxA-X-2290-19) | 34166 | 32982 | 34378 | 32284 |
