# Supplementary material for: Chronological reassessment of the Middle to Upper Paleolithic transition and Early Upper Paleolithic cultures in Cantabrian Spain
Source: PLoS One. 2018 Apr 18;13(4):e0194708. doi: 10.1371/journal.pone.0194708 (PMC5905894; doi:10.1371/journal.pone.0194708)
Supplement: S5 Table — (DOCX) [file pone.0194708.s006.docx]

S5 Table. Results of sensitivity test conducted on regional models

| **Boundary** | **Test** | **68.1% Credibility interval** | | **95.4% Credibility interval** | | |
| --- | --- | --- | --- | --- | --- | --- |
| End Mousterian | All available dates | 47298 | 46048 | 47914 | 45078 |  |
|  | Without Amalda VII- OxA-34933 | 47394 | 46078 | 48036 | 45052 |  |
| Start Aurignacian | All available dates | 42464 | 40932 | 43340 | 40478 |  |
|  | Without Castillo_16_OxA_22200 | 34376 | 33784 | 34604 | 33140 |  |
| End Aurignacian | All available dates | 41458 | 40630 | 42114 | 40300 |  |
|  | Without La_Vina_XI_OxA_19195 | 34730 | 34068 | 34930 | 33368 |  |
| Start Gravettian | All available dates | 35914 | 35206 | 36818 | 35030 |  |
|  | Without Aitz_III_Vb_sup_OxA_32419 | 35712 | 35052 | 36510 | 34872 |  |
