# Supplementary figures and images for: Chronological reassessment of the Middle to Upper Paleolithic transition and Early Upper Paleolithic cultures in Cantabrian Spain
Source: PLoS One. 2018 Apr 18;13(4):e0194708. doi: 10.1371/journal.pone.0194708 (PMC5905894; doi:10.1371/journal.pone.0194708)

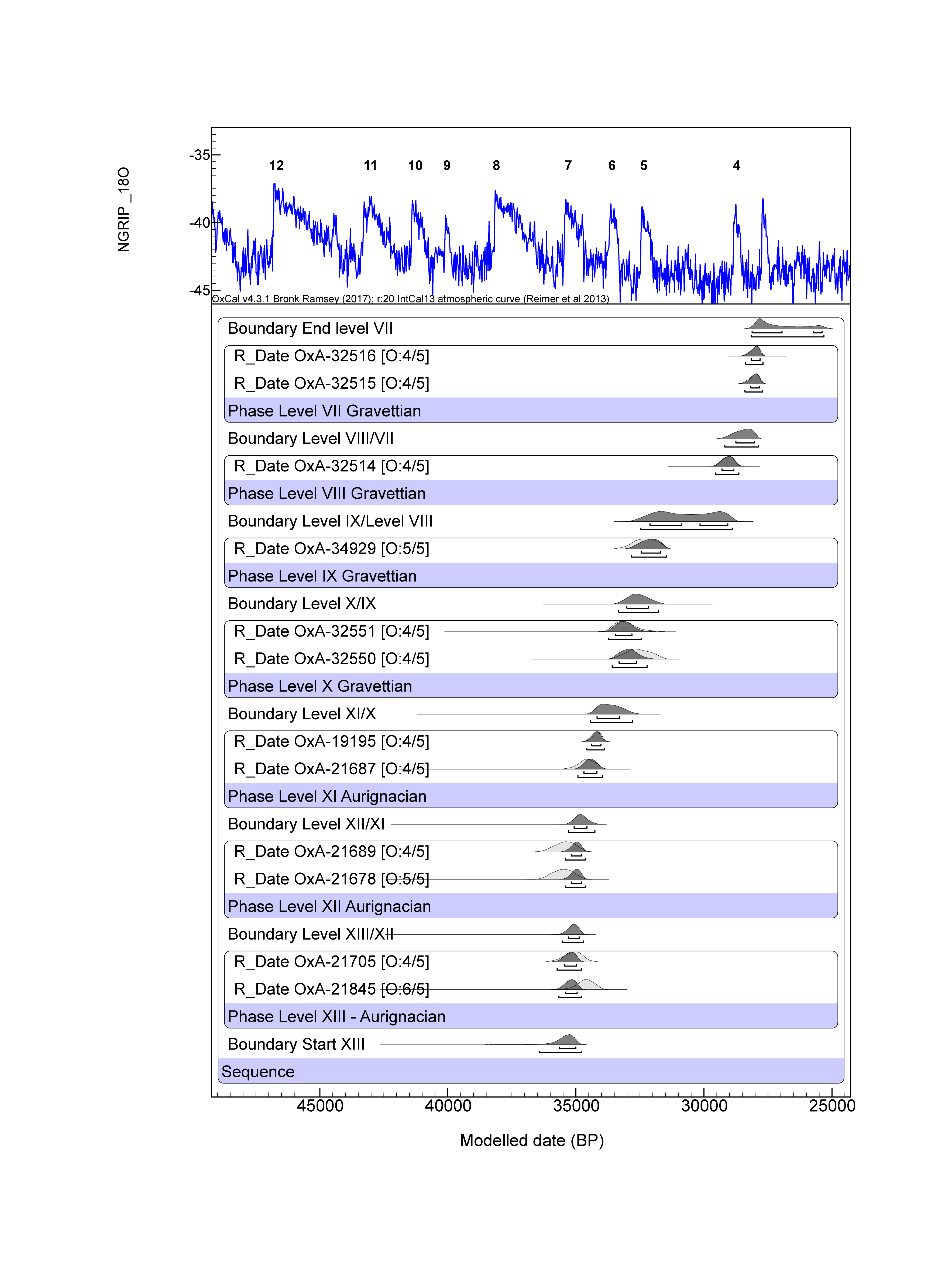

Supplement: S1 Fig — (TIF) [file pone.0194708.s007.tif]

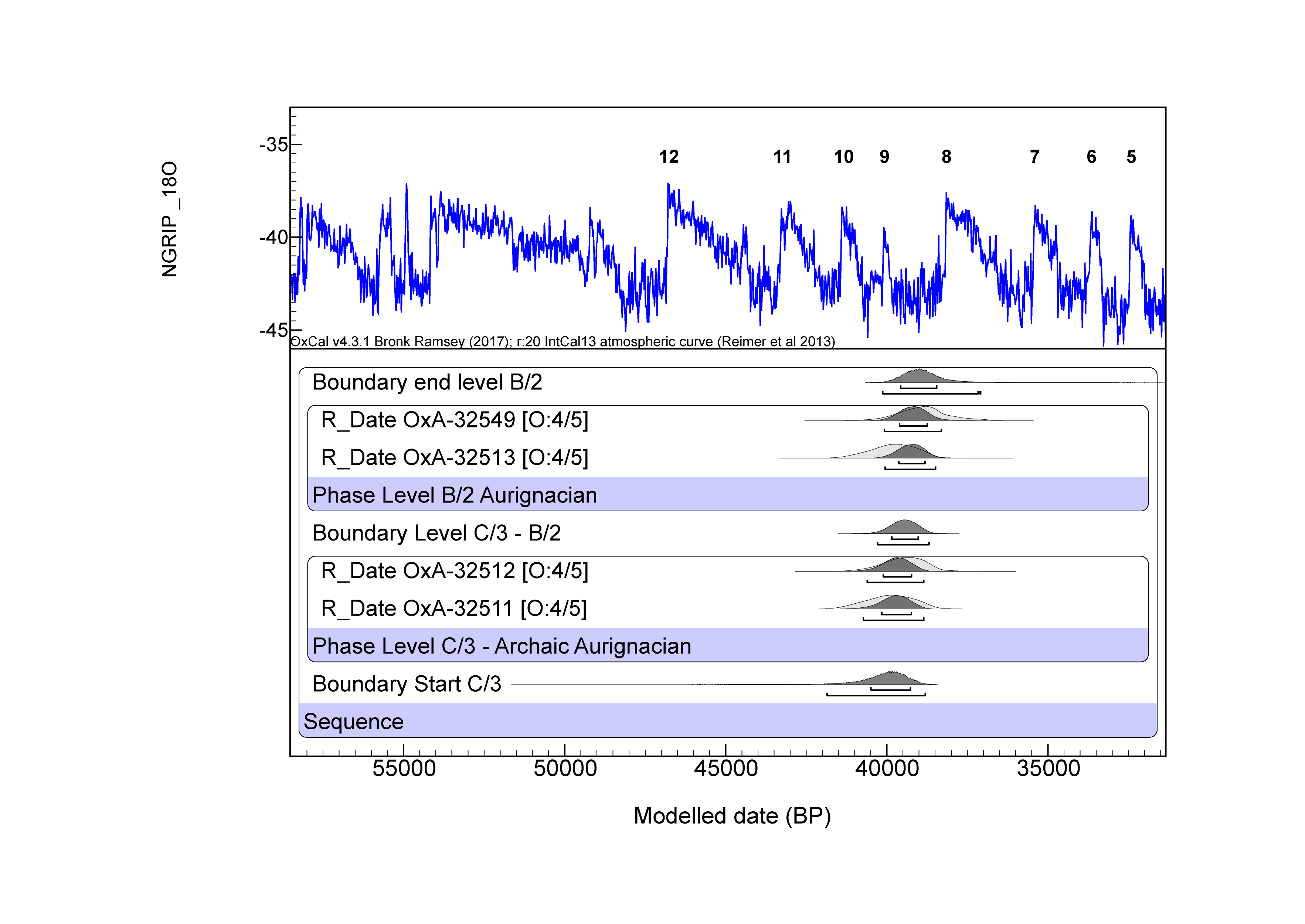

Supplement: S2 Fig — (TIF) [file pone.0194708.s008.tif]

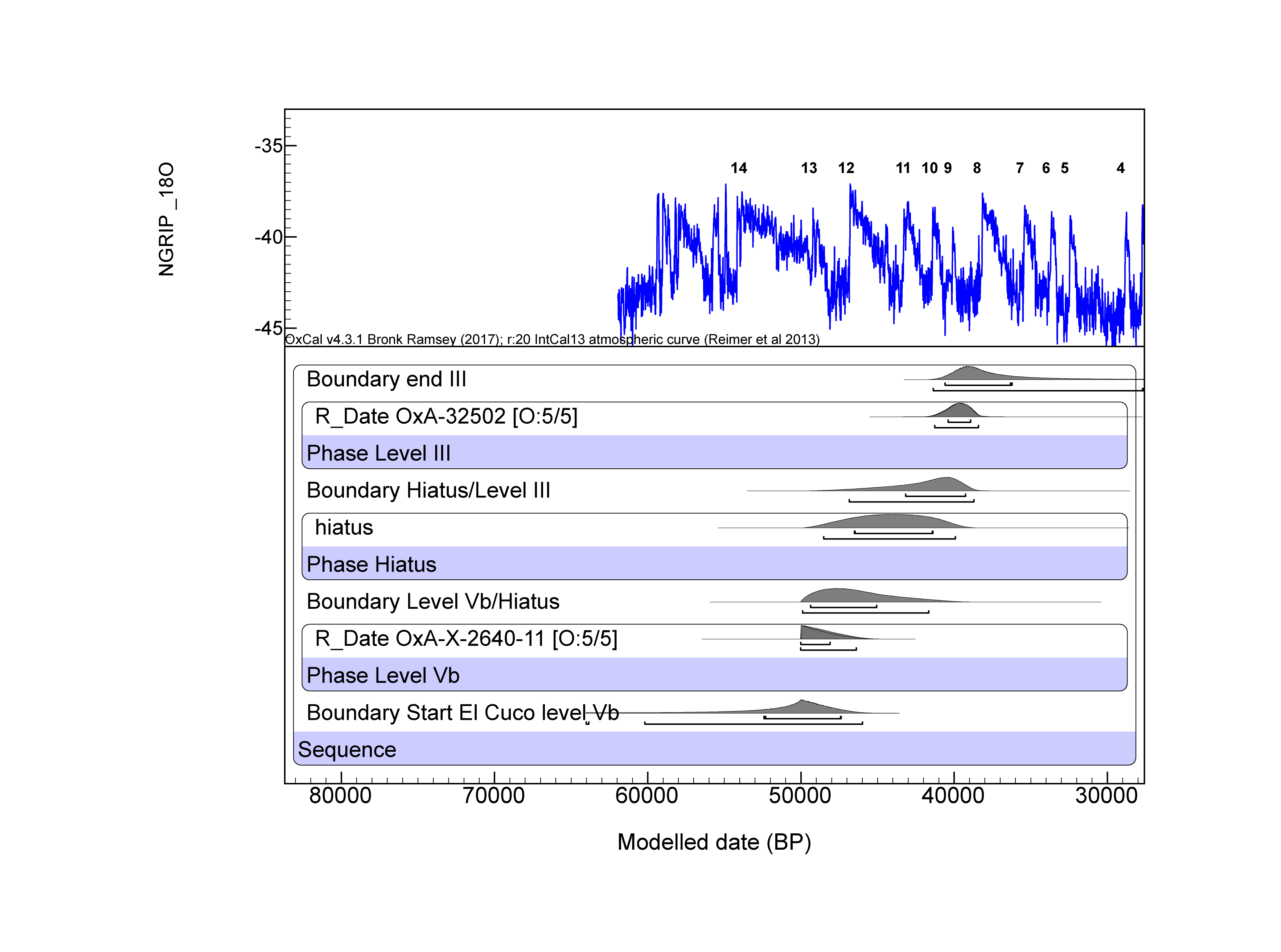

Supplement: S3 Fig — (TIF) [file pone.0194708.s009.tif]

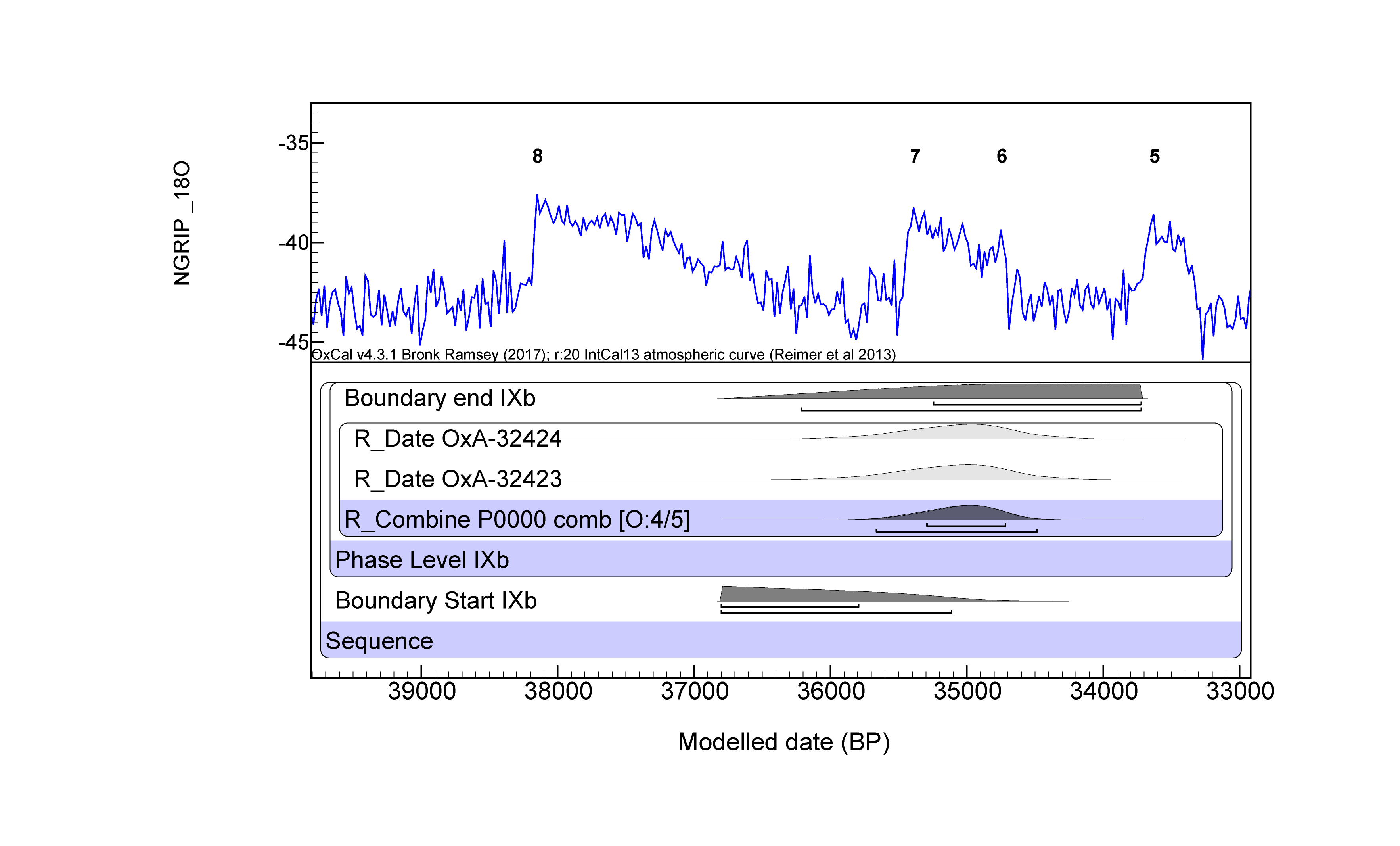

Supplement: S4 Fig — (TIF) [file pone.0194708.s010.tif]

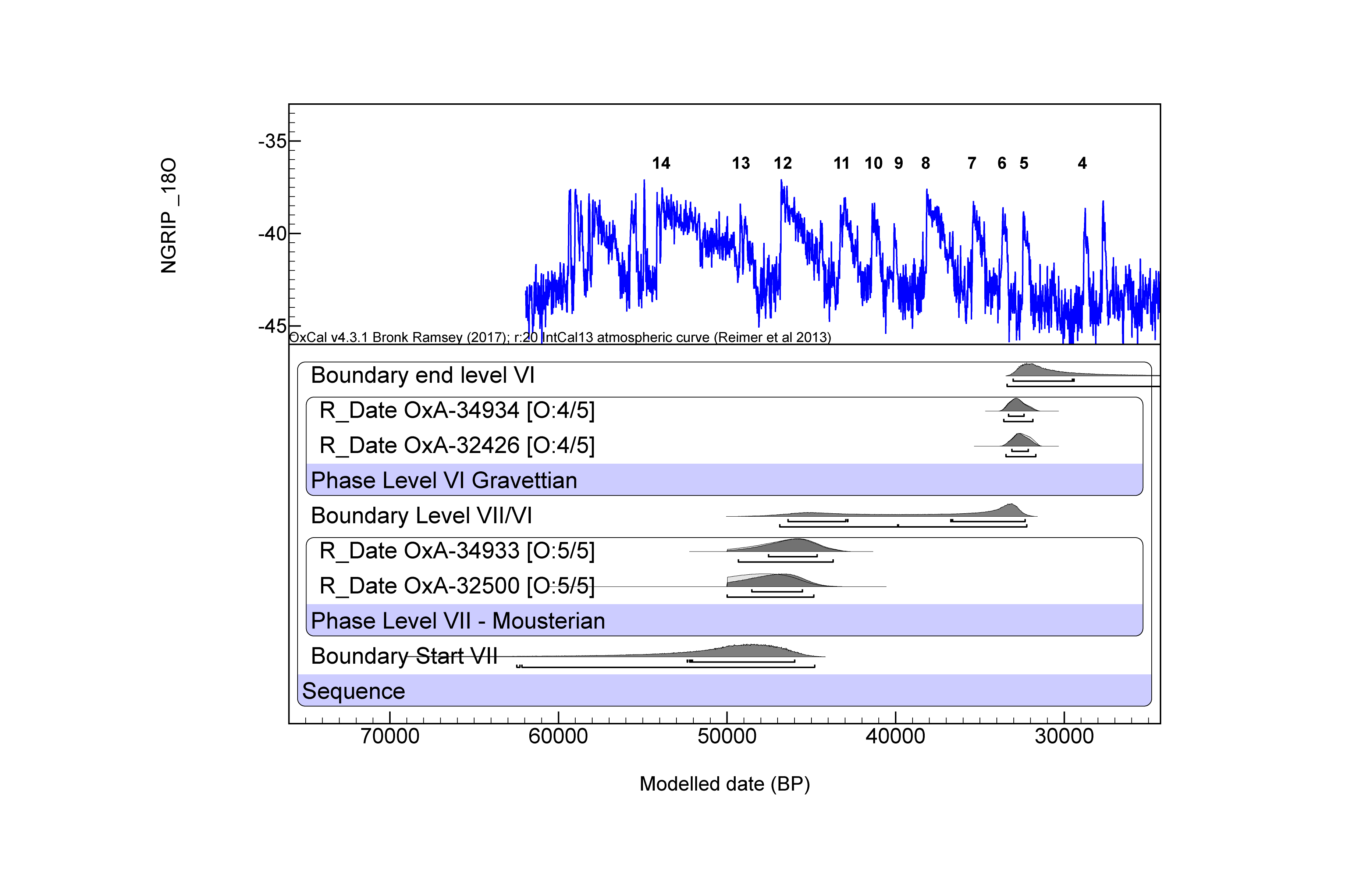

Supplement: S5 Fig — (TIF) [file pone.0194708.s011.tif]

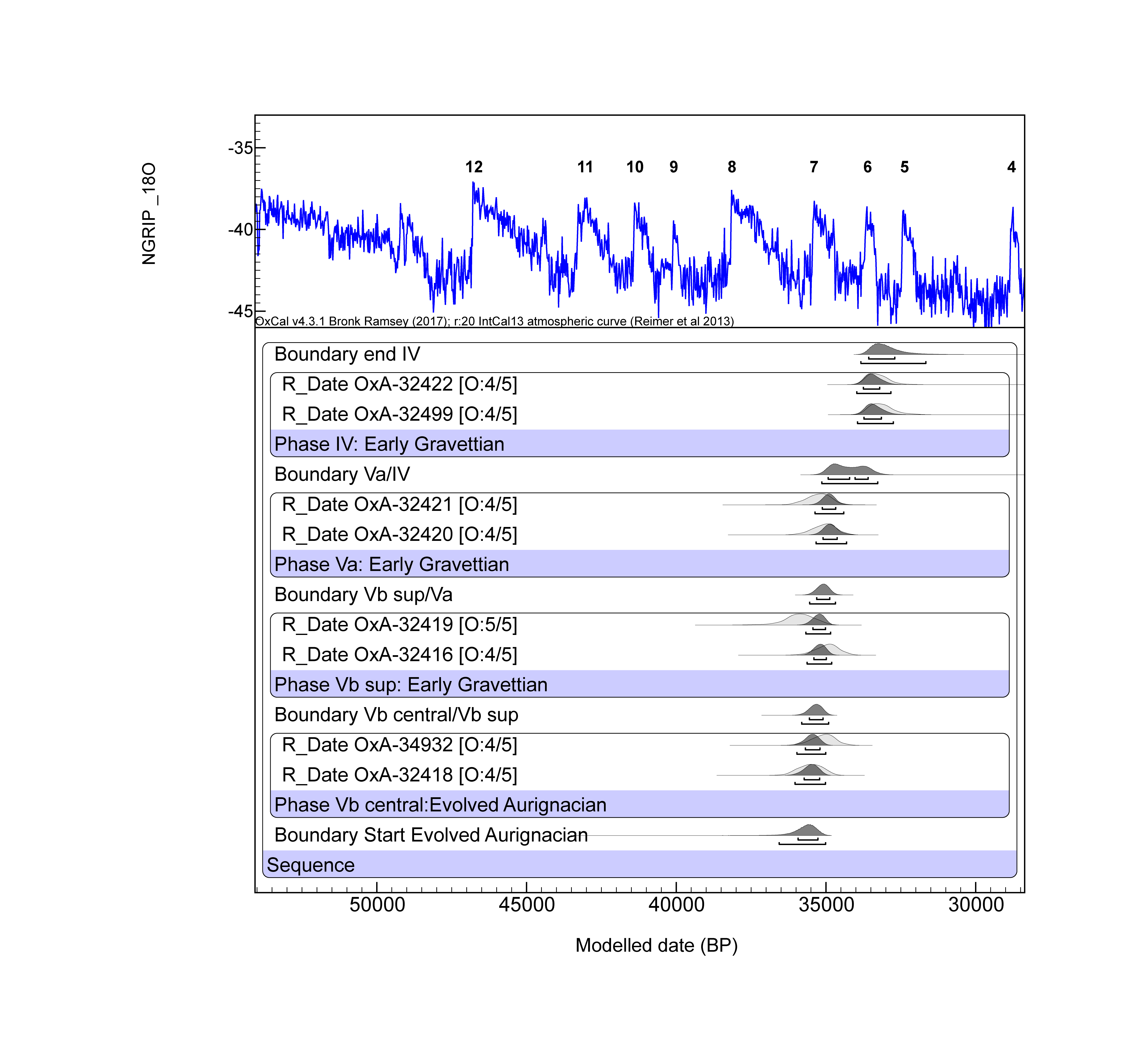

Supplement: S6 Fig — (TIF) [file pone.0194708.s012.tif]
