# Supplementary material for: Chronological reassessment of the Middle to Upper Paleolithic transition and Early Upper Paleolithic cultures in Cantabrian Spain
Source: PLoS One. 2018 Apr 18;13(4):e0194708. doi: 10.1371/journal.pone.0194708 (PMC5905894; doi:10.1371/journal.pone.0194708)
Supplement: S1 Code — (DOCX) [file pone.0194708.s013.docx]

**S1 Codes. CQL Individual Codes**

**CQL Code of model presented in S1 Figure**

Options()

{

Resolution=20;

};

{

Plot()

{

Outlier_Model("SSimple",N(0,2),0,"s");

Outlier_Model("General",T(5),U(0,4),"t");

Sequence()

{

Boundary("Start XIII");

Phase("Level XIII - Aurignacian")

{

R_Date("OxA-21845", 30650, 360)

{

Outlier("General", 0.05);

};

R_Date("OxA-21705", 31160, 380)

{

Outlier("General", 0.05);

};

Date("Level XIII");

};

Boundary("Level XIII/XII");

Phase("Level XII Aurignacian")

{

R_Date("OxA-21678", 31600, 400)

{

Outlier("General", 0.05);

};

R_Date("OxA-21689", 31500, 400)

{

Outlier("General", 0.05);

};

Date("Level XII");

};

Boundary("Level XII/XI");

Phase("Level XI Aurignacian")

{

R_Date("OxA-21687", 30600, 370)

{

Outlier("General", 0.05);

};

R_Date("OxA-19195", 30130, 170)

{

Outlier("General", 0.05);

};

Date("Level XI");

};

Boundary("Level XI/X");

Phase("Level X Gravettian")

{

R_Date("OxA-32550", 28560, 300)

{

Outlier("General", 0.05);

};

R_Date("OxA-32551", 28940, 310)

{

Outlier("General", 0.05);

};

Date("Level X");

};

Boundary("Level X/IX");

Phase("Level IX Gravettian")

{

R_Date("OxA-34929", 28360, 290)

{

Outlier("General", 0.05);

};

Date("Level IX");

};

Boundary("Level IX/Level VIII");

Phase("Level VIII Gravettian")

{

R_Date("OxA-32514", 25000, 200)

{

Outlier("General", 0.05);

};

Date("Level VIII");

};

Boundary("Level VIII/VII");

Phase("Level VII Gravettian")

{

R_Date("OxA-32515", 23960, 180)

{

Outlier("General", 0.05);

};

R_Date("OxA-32516", 23930, 180)

{

Outlier("General", 0.05);

};

Date("Level VII");

};

Boundary("end Level VII");

};

};

**CQL Code of model presented in S2 Figure**

Options()

{

Resolution=20;

};

{

Plot()

{

Outlier_Model("SSimple",N(0,2),0,"s");

Outlier_Model("General",T(5),U(0,4),"t");

Sequence()

{

Boundary("Start C/3");

Phase("Level C/3 - Archaic Aurignacian")

{

R_Date("OxA-32511", 35250, 700)

{

Outlier("General", 0.05);

};

R_Date("OxA-32512", 34850, 600)

{

Outlier("General", 0.05);

};

Date("Level C/3 - Archaic Aurignacian");

};

Boundary("Level C/3 - B/2");

Phase("Level B/2 Aurignacian")

{

R_Date("OxA-32513", 35150, 650)

{

Outlier("General", 0.05);

};

R_Date("OxA-32549", 34350, 600)

{

Outlier("General", 0.05);

};

Date("Level B/2 Aurignacian");

};

Boundary("end level B/2");

Order("Order")

{

};

};

**CQL Code of model presented in S3 Figure**

Options()

{

Resolution=20;

};

{

Plot()

{

Outlier_Model("SSimple",N(0,2),0,"s");

Outlier_Model("General",T(5),U(0,4),"t");

Sequence()

{

Boundary("Start El Cuco level Vb");

Phase("Level Vb")

{

R_Date("OxA-X-2640-11", 49500, 3900)

{

Outlier("General", 0.05);

};

Date("Level Vb");

};

Boundary("Level Vb/Hiatus");

Phase("Hiatus")

{

Date("hiatus");

};

Boundary("Hiatus/Level III");

Phase("Level III")

{

R_Date("OxA-32502", 35050, 650)

{

Outlier("General", 0.05);

};

Date("Level III");

};

Boundary("end III");

};

};

**CQL Code of model presented in S4 Figure**

Options()

{

Resolution=20;

};

{

Plot()

{

Outlier_Model("SSimple",N(0,2),0,"s");

Outlier_Model("General",T(5),U(0,4),"t");

Sequence()

{

Boundary("Start IXb");

Phase("Level IXb")

{

R_Combine("P0000 comb")

{

Outlier("General", 0.05);

R_Date("OxA-32423", 31140, 400)

{

Outlier("SSimple", 0.05);

};

R_Date("OxA-32424", 31110, 400)

{

Outlier("SSimple", 0.05);

};

Date("Level IXb");

};

Boundary("end IXb");

};

};

**CQL Code of model presented in S5 Figure**

Options()

{

Resolution=20;

};

{

Plot()

{

Outlier_Model("SSimple",N(0,2),0,"s");

Outlier_Model("General",T(5),U(0,4),"t");

Sequence()

{

Boundary("Start VII");

Phase("Level VII - Mousterian")

{

R_Date("OxA-32500", 44500, 2100)

{

Outlier("General", 0.05);

};

R_Date("OxA-34933", 42600, 1600)

{

Outlier("General", 0.05);

};

Date("Level VII Mousterian");

};

Boundary("Level VII/VI");

Phase("Level VI Gravettian")

{

R_Date("OxA-32426", 28540, 310)

{

Outlier("General", 0.05);

};

R_Date("OxA-34934", 28710, 300)

{

Outlier("General", 0.05);

};

Date("Level VI Gravettian");

};

Boundary("end level VI");

};

};

**CQL Code of model presented in S6 Figure**

Options()

{

Resolution=20;

};

{

Plot()

{

Outlier_Model("SSimple",N(0,2),0,"s");

Outlier_Model("General",T(5),U(0,4),"t");

Sequence()

{

Boundary("Start Evolved Aurignacian");

Phase("Vb central:Evolved Aurignacian")

{

R_Date("OxA-32418", 31600, 400)

{

Outlier("General", 0.05);

};

R_Date("OxA-34932", 31130, 390)

{

Outlier("General", 0.05);

};

Date("Level Vb central");

};

Boundary("Vb central/Vb sup");

Phase("Vb sup: Early Gravettian")

{

R_Date("OxA-32416", 30990, 390)

{

Outlier("General", 0.05);

};

R_Date("OxA-32419", 31950, 450)

{

Outlier("General", 0.05);

};

Date("Level Vb sup");

};

Boundary("Vb sup/Va");

Phase("Va: Early Gravettian")

{

R_Date("OxA-32420", 31090, 400)

{

Outlier("General", 0.05);

};

R_Date("OxA-32421", 31300, 400)

{

Outlier("General", 0.05);

};

Date("Level Va");

};

Boundary("Va/IV");

Phase("IV: Early Gravettian")

{

R_Date("OxA-32499", 29020, 320)

{

Outlier("General", 0.05);

};

R_Date("OxA-32422", 29130, 310)

{

Outlier("General", 0.05);

};

Date("Level IV");

};

Boundary("end IV");

Order()

{

};

};
