# Supplementary material for: Chronological reassessment of the Middle to Upper Paleolithic transition and Early Upper Paleolithic cultures in Cantabrian Spain
Source: PLoS One. 2018 Apr 18;13(4):e0194708. doi: 10.1371/journal.pone.0194708 (PMC5905894; doi:10.1371/journal.pone.0194708)
Supplement: S2 Code — (DOCX) [file pone.0194708.s014.docx]

**Start End -Final End Mousterian Cantabrian Region**

Options()

{

Resolution=20;

};

{

Plot()

{

Outlier_Model("SSimple",N(0,2),0,"s");

Outlier_Model("General",T(5),U(0,4),"t");

Sequence()

{

Boundary("Start End Mousterian, Cantabrian Region");

Phase("End Mousterian, Cantabrian Region")

{

R_Combine("P00002 comb:Esquilleu VI" )

{

R_Date("OxA-19965 Esquilleu VI", 43700, 1400)

{

Outlier("SSimple", 0.05);

};

R_Date("OxA-19966 Esquilleu VI", 44100, 1300)

{

Outlier("SSimple", 0.05);

};

};

R_Date("OxA-21986 Arrillor Lmc", 44900, 2100)

{

Outlier("General", 0.05);

};

R_Date("OxA-22655 Arrillor Lamc", 45600, 2300)

{

Outlier("General", 0.05);

};

R_Date("OxA-33516 Miron130", 48200, 3300)

{

Outlier("General", 0.05);

};

R_Combine("P00003 comb:La G�elga D,Lv9")

{

R_Date("OxA-19244La G�elga D,Lv9", 43700, 800)

{

Outlier("SSimple", 0.05);

};

R_Date("OxA-19245La G�elga D,Lv9", 44300, 1200)

{

Outlier("SSimple", 0.05);

};

};

Prior("Amalda_VII_OxA_32500", Amalda_VII_OxA_32500)

{

Outlier("General", 0.05);

};

Prior("Amalda_VII_OxA_34933", Amalda_VII_OxA_34933)

{

Outlier("General", 0.05);

};

};

Boundary("Final End Mousterian, Cantabrian Region");

};

};

};

**Start-End Aurignacian, Cantabrian Region**

Options()

{

Resolution=20;

};

{

Plot()

{

Outlier_Model("SSimple",N(0,2),0,"s");

Outlier_Model("General",T(5),U(0,4),"t");

Sequence()

{

Boundary("Aurignacian, Cantabrian Region");

Phase("Start Aurignacian, Cantabrian Region")

{

Prior("Castillo_16_OxA_22200", Castillo_16_OxA_22200)

{

Outlier("General", 0.05);

};

Prior("Labeko_VII_OxA_21766", Labeko_VII_OxA_21766)

{

Outlier("General", 0.05);

};

Prior("Labeko_VII_OxA_X_2314_43", Labeko_VII_OxA_X_2314_43)

{

Outlier("General", 0.05);

};

R_Date("OxA-32505 Cobrante VI", 35150, 650)

{

Outlier("General", 0.05);

};

Prior ("El_Cuco_III_OxA_32502", El_Cuco_III_OxA_32502)

{

Outlier("General", 0.05);

};

R_Combine("Labeko_VII:P23688 comb")

{

R_Date("OxA-21793 Labeko_VII", 35400, 650)

{

Outlier("SSimple", 0.05);

};

R_Date("OxA-21840 Labeko_VII", 35250, 650)

{

Outlier("SSimple", 0.05);

};

};

Prior("Labeko_VI_OxA_21778", Labeko_VI_OxA_21778)

{

Outlier("General", 0.05);

};

Prior("Covalejos_C_3_OxA_32511", Covalejos_C_3_OxA_32511)

{

Outlier("General", 0.05);

};

Prior("Covalejos_C_3_OxA_32512", Covalejos_C_3_OxA_32512)

{

Outlier("General", 0.05);

};

Prior("Covalejos_B_2_OxA_32513", Covalejos_B_2_OxA_32513)

{

Outlier("General", 0.05);

};

Prior("Covalejos_B_2_OxA_32549", Covalejos_B_2_OxA_32549)

{

Outlier("General", 0.05);

};

Prior("Labeko_V_OxA_21779", Labeko_V_OxA_21779)

{

Outlier("General", 0.05);

};

Prior("Labeko_V_OxA_21767", Labeko_V_OxA_21767)

{

Outlier("General", 0.05);

};

Prior("Labeko_IV_OxA_21768", Labeko_IV_OxA_21768)

{

Outlier("General", 0.05);

};

Prior("Labeko_IV_OxA_21780", Labeko_IV_OxA_21780)

{

Outlier("General", 0.05);

};

Prior("Aitz_III_Vb_central_OxA_34932", Aitz_III_Vb_central_OxA_34932)

{

Outlier("General", 0.05);

};

Prior("Aitz_III_Vb_central_OxA_32418", Aitz_III_Vb_central_OxA_32418)

{

Outlier("General", 0.05);

};

R_Combine("Ekain IXb:P00000 comb")

{

R_Date("OxA-32423 Ekain IXb", 31140, 400)

{

Outlier("SSimple", 0.05);

};

R_Date("OxA-32424 Ekain IXb", 31100, 400)

{

Outlier("SSimple", 0.05);

};

};

Prior("La_Vina_XIII_OxA_21705", La_Vina_XIII_OxA_21705)

{

Outlier("General", 0.05);

};

Prior("La_Vina_XIII_OxA_21845", La_Vina_XIII_OxA_21845)

{

Outlier("General", 0.05);

};

Prior("La_Vina_XII_OxA_21678", La_Vina_XII_OxA_21678)

{

Outlier("General", 0.05);

};

Prior("La_Vina_XII_OxA_21689", La_Vina_XII_OxA_21689)

{

Outlier("General", 0.05);

};

Prior("La_Vina_XI_OxA_21687", La_Vina_XI_OxA_21687)

{

Outlier("General", 0.05);

};

Prior("La_Vina_XI_OxA_19195", La_Vina_XI_OxA_19195)

{

Outlier("General", 0.05);

};

};

Boundary("End Aurignacian, Cantabrian Region");

Order("Order")

{

};

};

};

};

**Start-End Gravettian, Cantabrian Region**

Options()

{

Resolution=20;

};

{

Plot()

{

Outlier_Model("SSimple",N(0,2),0,"s");

Outlier_Model("General",T(5),U(0,4),"t");

Sequence()

{

Boundary("Gravetian, Cantabrian Region");

Phase("Start Gravettian, Cantabrian Region")

{

Prior("Aitz_III_Vb_sup_OxA_32419", Aitz_III_Vb_sup_OxA_32419)

{

Outlier("General", 0.05);

};

Prior("Aitz_III_Vb_sup_OxA_32416", Aitz_III_Vb_sup_OxA_32416)

{

Outlier("General", 0.05);

};

Prior("Aitz_III_Va_OxA_32420", Aitz_III_Va_OxA_32420)

{

Outlier("General", 0.05);

};

Prior("Aitz_III_Va_OxA_32421", Aitz_III_Va_OxA_32421)

{

Outlier("General", 0.05);

};

Prior("Aitz_III_IV_OxA_32499", Aitz_III_IV_OxA_32499)

{

Outlier("General", 0.05);

};

Prior("Aitz_III_IV_OxA_32422", Aitz_III_IV_OxA_32422)

{

Outlier("General", 0.05);

};

Prior("La_Vina_X_OxA_32551", La_Vina_X_OxA_32551)

{

Outlier("General", 0.05);

};

Prior("La_Vina_X_OxA_32550", La_Vina_X_OxA_32550)

{

Outlier("General", 0.05);

};

Prior("Amalda_VI_OxA_34934", Amalda_VI_OxA_34934)

{

Outlier("General", 0.05);

};

Prior("Amalda_VI_OxA_32426", Amalda_VI_OxA_32426)

{

Outlier("General", 0.05);

};

R_Date("OxA-32517 Llonin V", 28390, 350)

{

Outlier("General", 0.05);

};

Prior("La_Vina_IX_OxA_34929", La_Vina_IX_OxA_34929)

{

Outlier("General", 0.05);

};

Prior("La_Vina_X_OxA_21688", La_Vina_X_OxA_21688)

{

Outlier("General", 0.05);

};

R_Date("OxA-32519 Bolinkoba VI/F", 25280, 210)

{

Outlier("General", 0.05);

};

Prior("La_Vina_VIII_OxA_32514", La_Vina_VIII_OxA_32514)

{

Outlier("General", 0.05);

};

Prior("La_Vina_VII_OxA_32516", La_Vina_VII_OxA_32516)

{

Outlier("General", 0.05);

};

Prior("La_Vina_VII_OxA_32515", La_Vina_VII_OxA_32515)

{

Outlier("General", 0.05);

};

R_Date("Poz-66758 Morin 4", 23640, 190)

{

Outlier("General", 0.05);

};

R_Date("Poz-66759 Morin 4", 23790, 190)

{

Outlier("General", 0.05);

};

};

Boundary("End Gravettian, Cantabrian Region");

Order()

{

};

};

};

};
