# Supplementary material for: Correction: Chronological reassessment of the Middle to Upper Paleolithic transition and Early Upper Paleolithic cultures in Cantabrian Spain
Source: PLoS One. 2018 Jun 26;13(6):e0199954. doi: 10.1371/journal.pone.0199954 (PMC6019123; doi:10.1371/journal.pone.0199954)
Supplement: S1 Table — Collagen was extracted using the ultrafiltration protocol in all the samples. A contextual information of each archaeological level is provided, including a description of the lithic and bone artefacts and the archaeological context. Also, sample reference, animal species and skeletal element sampled including its taphonomic alterations is specified. For a reliable date, % yield should be >1%; %C >30% and C:N between 2.9 and 3.4 [65]. References to the original studies are cited and included below. (DOCX) [file pone.0199954.s001.docx]

S1Table. Radiocarbon AMS dates produced in this study. Collagen was extracted using the ultrafiltration protocol in all the samples. A contextual information of each archaeological level is provided, including a description of the lithic and bone artefacts and the archaeological context. Also, sample reference, animal species and skeletal element sampled including its taphonomic alterations is specified. For a reliable date, % yield should be >1%; %C >30% and C:N between 2.9 and 3.4 [Van Klinken 1999].References to the original studies are cited and included below.

| **Site** | **Level** | **Industry** | **Lithic and bone artefacts** | **Context** | **Species** | **Bone** | **Modification** | **Sample reference** | **Lab Code** | **Date (BP)** | **Error** | **%C** | **%N** | **C:N** | **d^13^C (‰ VPDB)** | **d^15^N (‰ AIR)** | **% collagen yield** | **References** |
| --- | --- | --- | --- | --- | --- | --- | --- | --- | --- | --- | --- | --- | --- | --- | --- | --- | --- | --- |
| Aitzbitarte III | IV | early Gravettian | 1,077 lithic artifacts, including 97 retouched tools. Burins and splintered pieces are the best represented tools. Among burins, there are Noailles and Busqué ones. Prismatic cores and cores on flakes for blade and bladelet production. Several refittings. Non-local raw materials. Bone tools are not characteristic. | 35I 139 | *Cervus elaphus* | Rib | Indeterminate breakage | AIT26 | OxA-32422 | 29130 | 310 | 44.6 | 7.6 | 3.2 | -19.9 | 7.6 | 9 | Rios-Garaizar et al. 2011 |
|  |  |  |  | 35J 139 | *Cervus elaphus* | Humerus | Indeterminate breakage | AIT12 | OxA-32499 | 29020 | 320 | 42.2 | 5.3 | 3.4 | -19.8 | 5.1 | 9.6 |  |
|  | Va | early Gravettian | 920 lithic artifacts, including 99 retouched tools. Burins and splintered pieces are the best represented tools. Among burins, there are Noailles, angle and dihedral burins. Prismatic cores and cores on flakes for blade bladelet production. Several refittings. Non-local raw materials. There are few, non-characteristic bone points. | 37k 148 51 | *Cervus elaphus* | Humerus | Anthropogenic breakage | AIT10 | OxA-32421 | 31300 | 400 | 44.6 | 3.3 | 3.2 | -20.0 | 3.3 | 7.1 |  |
|  |  |  |  | 37k 152 72 | *Cervus elaphus* | Tarsal | Anthropogenic breakage | AIT07 | OxA-32420 | 31090 | 400 | 45.8 | 3.2 | 3.2 | -20.1 | 3.2 | 8.7 |  |
|  | Vb sup | early Gravettian | 403 lithic artifacts, including 73 retouched tools. Burins and splintered pieces are the best represented tools. Among burins there are Noailles ones. Prismatic cores and cores on flakes for blade and bladelet production. Several refittings. Non-local raw materials. There is a losange shape bone point and several awls. | 37K 167 | *Equus sp* | Tibia | Anthropogenic breakage | AIT21 | OxA-32419 | 31950 | 450 | 42.6 | 1.7 | 3.2 | -20.3 | 1.7 | 6.3 |  |
|  |  |  |  | 36K 172 24 x.54 y.62 | *Equus sp* | Radius | Anthropogenic breakage | AIT19 | OxA-32418 | 31600 | 400 | 43.3 | 2.1 | 3.2 | -20.6 | 2.1 | 7.9 |  |
|  | Vb central | Evolved Aurignacian | 1,704 lithic artifacts, including 234 retouched tools. Splintered pieces, burins, end-scrapers, retouched bladelets and Aurignacian blades are the best represented tools. Among end-scrapers, there are several typical carinates. Among burins there are several atypically big Noailles, and nucleiform burins including Vachons and Busqué types. Retouched bladelets include typical Dufours. Prismatic cores are used for blade production, while carinated end-scrapers and nucleiform burins are dedicated to bladelet production. There are many refits, including long-production sequences. Local and distant raw materials are used. There is also an engraved pebble. Bone tools include awls and polishers. | 37J.166.78 | *Bos/Bison* sp. | Femur | Gnawing marks over cut marks | AIT28 | OxA-34932 | 31130 | 390 | 44 | 16.1 | 3.2 | -21.9 | 3.0 | 3 | Rios-Garaizar et al. 2011; Garate, Rios- Garaizar 2011 |
|  |  |  |  | 36J 163 277 X2Y41 | *Cervus elaphus* | Tibia | Anthropogenic breakage | AIT05 | OxA-32417 | 34900 | 600 | 44.4 | 9.5 | 3.2 | -19.6 | 9.5 | 5.5 |  |
|  |  |  |  | 37k 155 n17 | *Cervus elaphus* | Tibia | Anthropogenic breakage | AIT02 | OxA-32416 | 30990 | 390 | 42.9 | 2.9 | 3.2 | -19.7 | 2.9 | 4.5 |  |
| Ekain | IXb | Early Aurignacian | 111 lithic artifacts, including 3 end-scrapers, two of them typical carinates; three blades with Aurignacian retouch, one of them strangled, 1 denticulate and 3 retouched bladelets with no Dufour. Raw materials are distant. There are many reffitings, including bladelets refitted onto carinated end-scrapers. | Ek 3A 325 | *Cervus elaphus* | Mandible | Anthropogenic breakage | EKA02(Y) | OxA-32423 | 31140 | 400 | 43.7 | 4.4 | 3.2 | -19.9 | 4.4 | 5 | Rios-Garaizar 2011 |
|  |  |  |  | Ek 3A 325 | *Cervus elaphus* | Mandible | Anthropogenic breakage | EKA05 | OxA-32424 | 31110 | 400 | 42.7 | 4.1 | 3.3 | -19.7 | 4.1 | 7.6 |  |
|  | Xa | Châtelperronian | 12 lithic artifacts including 6 retouched tools. There are 2 Châtelperron points, a fragment of micro-gravette, a backed blade, a retouched blade and a Dufour bladelet. Lithic raw materials are from distant sources. There is evidence of blade production using bidirectional ‘decalé’ method. No bone tools | 3B.375 | *Rupicapra sp* | Radius | Gnawing marks/Anthropogenic breakage | EKA08 | OxA-34930 | 34350 | 550 | 45 | 16.3 | 3.2 | -20.0 | 2.8 | 5.2 | Rios-Garaizar et al. 2012 |
| Amalda | V | Gravettian | 797 lithic artifacts, including 102 retouched tools, Burins (with few Noailles) and backed bladelets are the best represented types. Raw material used is flint. There are few heavily exploited cores, including carinated bladelet cores. There are two bone point fragments. | A 12C 83 | *Cervus elaphus* | Radius | Anthropogenic breakage | AMA32 | OxA-32427 | 14745 | 75 | 43.3 | 4 | 3.3 | -20.0 | 4.0 | 2.4 | Baldeón 1990 |
|  | VI | Gravettian | 1,962 lithic remains, including 280 retouched tools. Burins are the best represented tools, specially the Noailles type. There is a single atypical Gravette Point. Tools on flakes are relatively abundant, and retouched bladelets scarce. Flint is the preferred raw material. There are few cores, including typical prismatic and carinated cores for bladelet production. There are poorly elaborated bone tools and four beads, two on *Littorina obtusata*, one on fox canine and the another one on a red deer atrophic canine. | A 7C 167 20 | *Cervus elaphus* | Tibia | Anthropogenic breakage | AMA26 | OxA-32426 | 28540 | 310 | 43.9 | 3.7 | 3.3 | -20.2 | 3.7 | 4.2 | Baldeón 1990 |
|  |  |  |  | A9E 112 1 | *Cervus elaphus* | Tibia | Cut marks | AMA06 | OxA-32501 | 886 | 26 | 42.1 | 4.5 | 3.3 | -22.2 | 4.2 | 13.8 |  |
|  |  |  |  | 8D 149 | *Cervus elaphus* | Metatarsal | Impact notch | AMA25 | OxA-34934 | 28710 | 300 | 43.9 | 16.1 | 3.2 | -20.6 | 3.6 | 4.6 |  |
|  | VII | Mousterian | 1,084 lithic artifacts, including 267 tools. Side-scrapers are the best represented raw material, followed by retouched flakes, denticulates and naturally backed knifes. There is a single Abri Audi knife and a few Mousterian points. Cleavers and bifaces are also present. Raw materials are variated, including distant flint and local rocks (Tobaceous lutite, ophite…). There is discoid production on local raw materials and Levallois production on flint. It is noteworthy the use of ramified productions for the procurement of small flakes. There are few retouchers made on bone diaphysis. | A 12D 124 | *Equus sp* | Metacarpal | Impact notch | AMA13 | OxA-32425 | 28640 | 310 | 42.4 | 4.1 | 3.2 | -20.8 | 4.1 | 2.3 | Rios-Garaizar 2010 |
|  |  |  |  | A8G 204 13 | *Cervus elaphus* | Metatarsal | Anthropogenic breakage | AMA02 | OxA-32500 | 44500 | 2100 | 42.1 | 3.8 | 3.4 | -20.2 | 3.8 | 2.5 |  |
|  |  |  |  | 7G 221 16 X62 Y82 | *Cervus elaphus* | Tibia | Anthropogenic breakage | AMA05 | OxA-34933 | 42600 | 1600 | 43.7 | 15.9 | 3.2 | -21.2 | 2.9 | 6.7 |  |
| Axlor | IV | Mousterian | 13,086 lithic artifacts, including 2,570 retouched tools. Side-scrapers are two-thirds of the total (with many Quina side-scrapers), followed by retouched flakes, denticulates and points. Distant flint represents the 80% of the total of rocks, followed by lutite, quartz and quartzite. The technology is typically Quina, and the ramified production and use of re-sharpening tools is found extensively. Bone retouchers are very abundant. | AX.11C.290.149 | *Cervus elaphus* | Phalanx 2 | Anthropogenic breakage | AXL39 | OxA-32428 | >49300 | - | 42.3 | 3.5 | 3.3 | -19.8 | 3.5 | 3.1 | Rios-Garaizar 2012 |
|  |  |  |  | AX.11C.300. 178 | *Cervus elaphus* | Carpal | Complete | AXL42 | OxA-32429 | >49900 | - | 43 | 6.3 | 3.3 | -19.2 | 6.3 | 6 |  |
| Bolinkoba | VI/F | Gravettian | 2,700 lithic artifacts, including 760 retouched tools. Burins are the best represented tool type, including many typical Noailles burins; followed by retouched blades, truncations and end-scrapers. There are few typical Gravette points, and some Vachons ones. Bladelet tools are not so well-represented. Raw materials are dominated by distant flint. Among bone tools, there is a typical Isturitz point and many bone point fragments. There are 15 *Littorina* and 4 *Nassa reticulata* beads. | B.1G.Z=30. | *Cervus elaphus* | Phalanx 1 | Anthropogenic breakage | BOL02 | OxA-32518 | 10605 | 50 | 43.3 | 15 | 3.4 | -20.8 | 4.2 | 8.9 | Garate Maidagan 2012, Rios-Garaizar and Garate Maidagan 2014 |
|  |  |  |  | B.1E.Z=85. | *Cervus elaphus* | Phalanx 2 | Complete | BOL03 | OxA-32519 | 25280 | 210 | 43.7 | 15.1 | 3.4 | -19.1 | 6.8 | 7.2 |  |
| El Cuco | III | Gravettian | 20,532 lithic artifacts including 456 formal tools (*sensu* Muñoz et al. 2007). Retouched blades and flakes are the best represented type, followed by end-scrapers including nosed and carinated types. Burins are scarce and among retouched bladelets there are many typical Dufour. Raw materials are dominated by flint, including many distant flints. In this study, we noted the presence of typical prismatic bladelet cores. There are few bone tools, including a bipoint and a *Dentalium vulgae* bead. | Box:20, Spit:5, Sondeage:1, No: 460 | *Cervus elaphus* | Tibia | Impact notch | CUC03 | P-38623 | Failed | Failed | - | - | - | - | - | - | Muñoz Fernádez et al. 2007, This study |
|  |  |  |  | Box:20, Spit:4, Sondeage:1, No:289 | *Cervus elaphus* | Metatarsal | Indeterminate breakage | CUC08 | OxA-32502 | 35050 | 650 | 42.9 | 14.8 | 3.4 | -20.1 | 4.2 | 1.1 |  |
|  | Vb | Gravettian | 4,474 lithic artifacts, including 107 retouched tools. Among them, many end-scrapers were found, but most of them were simple flakes with facetted platforms. There is a single atypical burin and many pieces classifiable as side-scrapers and denticulates. No bone tools found. | Box:20, No:392 | *Cervus elaphus* | Metatarsal | Indeterminate breakage | CUC10 | OxA-X-2640-11 | 49500 | 3900 | 41.7 | 14.4 | 3.4 | -20.6 | 4.2 | 0.6 | Muñoz Fernádez et al. 2007 |
| Cobrante | V | Aurignacian | 178 lithic artifacts, including 18 retouched tools. Among them, there are two pieces with flat parallelretouch, one of them classified by Muñoz Fernandez and Santamaría (2009) as an Aurignacian blade. Raw materials are dominated by flint with a significant component of quartz. There is a bipoint and a bone awl. | Box:15, Square:H18, No:559 | *Cervus elaphus* | Phalanx 1 | Anthropogenic breakage | COB08 | OxA-32503 | 18550 | 100 | 44.4 | 15.4 | 3.4 | -19.8 | 2.0 | 5.1 | Muñoz Fernandez and Santamaría 2009, Tejero Cáceres 2009 |
|  |  |  |  | Box:15, Square:H18, No:562 | *Cervus elaphus* | Femur | Anthropogenic breakage | COB12 | OxA-32504 | 18310 | 100 | 43.3 | 15.1 | 3.4 | -20.1 | 2.0 | 6.2 |  |
|  | VI | ProtoAurignacian | 1,287 lithic artifacts, including 60 retouched tools. Among them few end-scrapers including one on an Aurignacian blade and a few retouched bladelets, including typical Dufour. “Archaic” looking tools like side-scrapers on flake and denticulates are abundant. Raw materials include flint, quartzite and marl. Bone tools include three point fragments. | Box:15, Square:H18, Sector:W, Z:267-303, No:1426 | *Cervus elaphus* | Phalanx 2 | Anthropogenic breakage | COB15 | OxA-32505 | 35150 | 650 | 41.9 | 14.6 | 3.4 | -19.9 | 3.0 | 2.7 | Muñoz Fernández and Santamaría 2009, Tejero Cáceres 2009 |
|  |  |  |  | Box:15, no number | *Cervus elaphus* | Femur | Anthropogenic breakage | COB18 | OxA-32506 | >44800 | - | 42.7 | 14.8 | 3.4 | -20.2 | 3.6 | 2.6 |  |
| El Ruso | IVb | Evolved Aurignacian | 266 lithic remains including 45 retouched tools. There are several core-like end-scrapers, few atypical burins and 1 Dufour bladelet. Flint is the most used raw material. There are 5 bone points, 1 awl, 1 spatula, two perforated Littorina and one perforated fox canine. There are also few engraved bone fragments with lineal traits. | Box: 483, No:2901, RU84, Square:J-4, Sc:1, Z:100 | *Cervus elaphus* | Tibia | Anthropogenic breakage | RUS06 | OxA-32507 | 28510 | 300 | 42.4 | 14.6 | 3.4 | -20.1 | 4.1 | 2.8 | Muñoz Fernández 1991 |
|  |  |  |  | Box: 483, No:2943, RU84, Square:K-2, Sc:4, Z:100-106 | *Cervus elaphus* | Humerus | Anthropogenic breakage | RUS07 | P-38631 | Failed | Failed | - | - | - | - | - | - |  |
| El Otero | IV | Aurignacian V (Late Aurignacian) | 307 lithic artifacts, 96 of them retouched. There are carinated and nosed end-scrapers, Aurignacian blades and a single atypical Dufour bladelet. Burins are also present, with some examples of burins on truncation and core-like burins. Flint is the most used raw material with some distal flint present Blade technology. There are several bone point fragments with circular and oval sections and three perforated teeth. | Box:1715 | *Cervus elaphus* | Mandible | Anthropogenic breakage | OTE06 | OxA-32508 | 15990 | 80 | 41.2 | 14.2 | 3.4 | -20.0 | 3.3 | 1.9 | González Echegaray 1966, Bernaldo de Quirós 1982, Rios-Garaizar et al. 2013 |
|  | V | Aurignac IV | 57 lithic remains, 37 of them retouched. End-scrapers are well-represented, one of them a thumbnail. There are simple burins and one flat burin, also some raclettes. There are two perforated teeth and no bone points. | Box:1708, Bag: 3379 | *Cervus elaphus* | Metacarpal | Anthropogenic breakage | OTE022 | OxA-32509 | 12340 | 55 | 43.5 | 14.9 | 3.4 | -19.6 | 2.0 | 2.9 | González Echegaray 1966, Bernaldo de Quirós 1982 |
|  |  |  |  | Box:1708, Bag: 3379 | *Cervus elaphus* | Phalanx 1 | Anthropogenic breakage | OTE023 | OxA-32510 | 10585 | 50 | 42.6 | 14.8 | 3.4 | -20.3 | 3.9 | 10.8 |  |
|  | VI | Aurignacian III | 54 lithic remains, 34 of them retouched. There are carinated and nosed end-scrapers and pieces classified as Aurignacian blades. Two bone point fragments (flat and round section). | Box:1717, Bag: 3473 | *Cervus elaphus* | Metatarsal | Impact notch | OTE025 | OxA-32585 | 12415 | 55 | 46.1 | 15.9 | 3.4 | -21.0 | 4.8 | 3.1 | Arrizabalaga 1995, Bernaldo de Quirós 1982, González Echegaray 1966 |
| Morín | 7c | Early Aurignacian | 344 retouched tools. Many end-scrapers, including carinated forms. Burins are also well-represented (dihedral and angle burins). There are few Aurignacian blades and several Dufour bladelets. Several bone point fragments (circular section) and a perforated red deer canine were found. | Corte:1, Square:4 | *Cervus elaphus* | Phalanx 2 | Complete | MOR07 | P-38636 | Failed | Failed |  |  |  |  |  |  | Bernaldo de Quirós 1982 |
| Covalejos | B (2) | Early Aurignacian | Rich lithic collection attributed without doubts to the Aurignacian. Rich bone industry, with 4 split based points, 1 polisher, 1 chisel and 4 retouchers. There are 11 perforated teeth and 12 marine gastropod perforated shells. | 2002.H5B1.C.5664 | *Cervus elaphus* | Mandible | Anthropogenic breakage | COV14 | OxA-32549 | 34350 | 600 | 42.3 | 15.3 | 3.2 | -20.0 | 8.2 | 2.4 | Sanguino González and Montes Barquín 2005, Tejero Cáceres 2013 |
|  |  |  |  | 2002.H5D2.C.5695 | *Cervus elaphus* | Tibia | Anthropogenic breakage | COV21 | OxA-32513 | 35150 | 650 | 41.5 | 14.4 | 3.4 | -20.1 | 5.9 | 9.8 |  |
|  | C (3) | ProtoAurignacian | Less rich lithic assemblage in comparison to Level C (3) with several typical Dufour bladelets. Several worked bone fragments and two perforated teeth found. | 1999.I4.B.4480 | *Cervus elaphus* | Femur | Anthropogenic breakage | COV27 | OxA-32512 | 34850 | 600 | 44.8 | 15.6 | 3.4 | -20.5 | 3.9 | 10.7 | Sanguino González and Montes Barquín 2005, Tejero Cáceres 2013 |
|  |  |  |  | 2002.H5D4.B.505 | *Cervus elaphus* | Long bone shaft | Indeterminate breakage | COV23 | OxA-32511 | 35250 | 700 | 41.5 | 14.6 | 3.3 | -19.9 | 2.9 | 5.6 |  |
| La Viña | VII | Gravettian | High representation of backed bladelets and burins, with presence of (micro) Gravette points and a few Noailles burins, thick burins and endscrapers aimed at bladelet production | G25-capa10-Sub4-283 | *Cervus elaphus* | Tibia | Indeterminate breakage | VIN75 | OxA-32515 | 23960 | 180 | 43.3 | 15 | 3.4 | -20.4 | 4.4 | 4.8 | Fortea, 1992; Martínez, 2015 |
|  |  |  |  | G25-capa9-Sub4-254 | *Cervus elaphus* | Metatarsal | Anthropogenic breakage | VIN84 | OxA-32516 | 23930 | 180 | 40.9 | 14.2 | 3.4 | -20.7 | 6.2 | 1.8 |  |
|  | VIII | Gravettian | High representation of backed bladelets, with more (micro) Gravette points and a few Noailles burins in comparison to Levels IX and X; a Font-Robert point fragment, thick burins and endscrapers aimed at bladelet production were found. | F25-capa19- sub6-516 | *Cervus elaphus* | Metatarsal | Impact notch | VIN69 | P-38643 | Failed | Failed | - | - | - | - | - | - | Fortea, 1992; Martínez, 2015 |
|  |  |  |  | G26-capa5-sub1-190 | *Cervus elaphus* | Femur | Impact notch | VIN73 | OxA-32514 | 25000 | 200 | 41.8 | 14.4 | 3.4 | -20.3 | 4.6 | 3 |  |
|  | IX | Gravettian | High representation of backed bladelets and burins, with presence of (micro) Gravette points and Noailles burins, thick burins and endscrapers aimed at bladelet production and a *Calamites* deliberately modified. | F27-capa17-sub1-1244 | *Cervus elaphus* | Phalanx 3 | Indeterminate breakage | VIN55 | OxA-34929 | 28360 | 290 | 42.8 | 15.5 | 3.2 | -20.5 | 5.6 | 2.2 | Fortea, 1992; Martínez, 2015 |
|  | X | Gravettian | Burins and retouched bladelets dominate the assemblage, with presence of Noailles burins and backed bladelets, thick burins and endscrapers aimed at bladelet production | G26-capa11-sub5-671 | *Cervus elaphus* | Humerus | Anthropogenic breakage | VIN43 | OxA-32550 | 28560 | 300 | 42 | 15.1 | 3.2 | -20.7 | 5.7 | 2.7 | Fortea, 1992; Martínez, 2015 |
|  |  |  |  | G26-capa11-sub7-685 | *Cervus elaphus* | Humerus | Anthropogenic breakage | VIN47 | OxA-32551 | 28940 | 310 | 42.6 | 15.1 | 3.3 | -20.0 | 5.1 | 1.3 |  |
| Llonín | V | Gravettian | Backed bladelets dominate the assemblage, presence of Vachon points and a possible shouldered point. Other tools and cores are scarce. | L-97 - B6 - 300. Galeria | *Cervus elaphus* | Metatarsal | Anthropogenic breakage-cut marks | LLO80 | OxA-32517 | 20040 | 120 | 42.1 | 14.6 | 3.4 | -20.6 | 2.8 | 3.8 | Fortea *et al* 1995, 1999; Martínez, 2015 |
|  |  |  |  | L-97 - B6 - 309. Galeria | *Cervus elaphus* | Femur | Anthropogenic breakage | LLO81 | OxA-X-2640-48 | 28390 | 350 | 42.6 | 14.9 | 3.3 | -20.2 | 4.5 | 1.8 |  |
|  | VI | Mousterian | Quartzite substrate tools and two endscrapers. Flakes dominate and blades are scarce. No cores found. | L-95 - C4 - 1082. galeria | *Cervus elaphus* | Ulnae | Complete | LLO83 | P-38649 | Failed | Failed |  |  |  |  |  |  | Fortea *et al,* 1999; Rasilla and Santamaría, 2011-2012 |
